# Synthesis and resolution of a 1,1′-biazulene analogue of BINOL†

**DOI:** 10.1039/d5ra02520f

**Published:** 2025-05-16

**Authors:** Anthony P. Gee, Tiberiu-M. Gianga, Gabriele Kociok-Köhn, G. Dan Pantoş, Simon E. Lewis

**Affiliations:** a Department of Chemistry, University of Bath Bath BA2 7AY UK S.E.Lewis@bath.ac.uk; b Physical Structure Characterization Facility, University of Bath Bath BA2 7AY UK; c Institute of Sustainability and Climate Change, University of Bath Bath BA2 7AY UK

## Abstract

Biaryls exhibiting axial chirality have been extensively exploited in fields such as asymmetric catalysis, but the biaryl linkage typically consists of benzenoid aromatic rings, with non-benzenoid biaryls being scarce. Here we report the first preparation of a (non-benzenoid) 1,1′-biazulene-2,2′-diol (“1,1′-BAzOL”) in enantiopure form and determine its barrier to racemisation. Furthermore we transformed a 1,1′-biazulene-2,2′-diol into the corresponding 2,2′-bis(phosphonate), thereby demonstrating functional group interconversion through cross coupling and highlighting the potential for diversification.

## Abstract 2

A 1,1′-biazulenyl-2,2′-diol (“1,1′-BAzOL”) has been prepared in enantiopure form. The barrier to racemisation of this 1,1′-BAzOL has been determined and synthesis of an analogous 2,2′-bis(phosphonate) has also been achieved.

## Graphical abstract

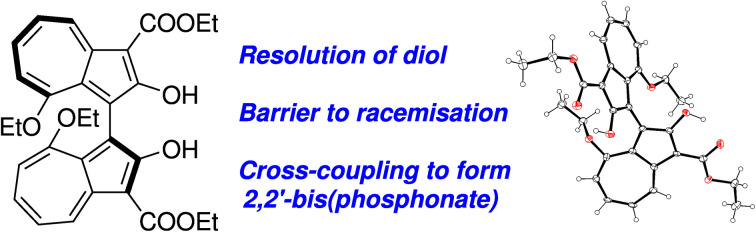

## Introduction

**Fig. 1:**
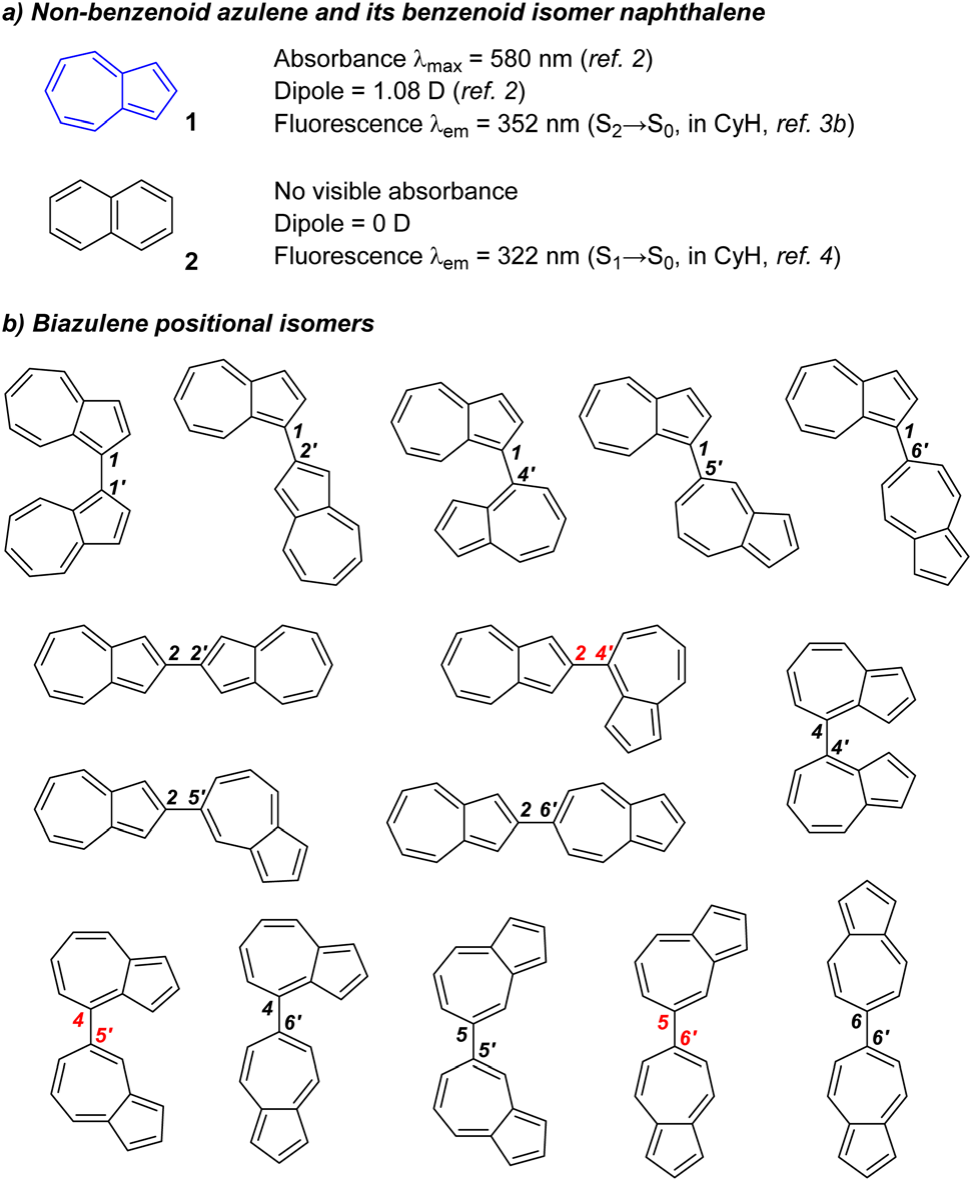
Naphthalene, azulene and biazulene isomers.

Azulene 1 is a non-benzenoid 10π bicyclic aromatic compound, known for its blue colour,^1^ large dipole^2^ and anomalous fluorescence.^3^ Each of these properties differ from those of the corresponding benzenoid isomer naphthalene 2 (Fig. 1a).^4^ Azulene derivatives have been used in multiple applications, including in fluorescence imaging,^5^ colorimetric sensing,^6^ solar cells,^7^ photothermal therapy,^8^ dyestuffs,^9^ organic field-effect transistors (OFETs),^10^ and other optoelectronics.^11^

Biazulenes are a group of biaryls for which 15 different positional isomers can be envisaged (Fig. 1b) whose structural and electronic properties can vary significantly depending on the position of the biaryl linkage as well as the substituents.^12^ The first examples of biazulene synthesis, reported in 1968,^13^ were of 1,1′- and 2,2′-biazulenes formed by (multistep) dimerisation of the natural product guaiazulene (this can also undergo direct oxidative dimerisation to give 1,1′-,^14,15^ 1,2′-^15,16^ and 1,5′-^16^ biazulenes). Another early report describes the synthesis of 1,1′- and 2,2′-biazulenes by Ullmann coupling of the corresponding haloazulene monomers^17^ (1,2′- and 2,6′-biazulenes were also isolated from mixtures arising from coupling of two different monomers). As of now, 2,4-, 4,5′- and 5,6′-biazulenes remain unknown to the best of our knowledge, but examples of all other positional isomers have been reported. Most extensively studied are the 1,1′-biazulenes, which have been synthesised by approaches including reductive coupling,^18^ oxidative dimerisation either electrochemically^19^ or using FeCl_3_,^20,21^ MnO_2_,^22^ (NH_4_)_2_S_2_O_8_,^23^ CuBr/O_2_,^24^ DDQ^25^ or electrophilic halide sources,^26^ as well as by photochemical methods,^27^ sulfide/sulfoxide activation,^28^ C–H activation,^29^ Suzuki coupling^30^ and aromatisation of a partially saturated precursor.^31^ Less common are the 1,2′-,^32^ 1,4′-,^33^ 1,5′-^16^ and 1,6′-^34,35^ biazulenes. The “linear” biazulenes (*i.e.* the 2,2′-,^36^ 2,6′-^34,37^ and 6,6′-^38^ isomers) have found diverse applications in organic functional materials, *e.g.* in surface modifiers,^39^ OFETs,^40,41^ supercapacitors,^42^ molecular rectifiers,^43^ hole-transport materials for perovskite solar cells,^44^ enhancers of π-π stacking^45^ and memristors.^46^ The remaining known biazulene isomers (2,5′-,^47^ 4,4′-,^12*c*,33,48^ 4,6′-,^12*c*,49^ and 5,5-^50^) have been reported only rarely.

**Fig. 2:**
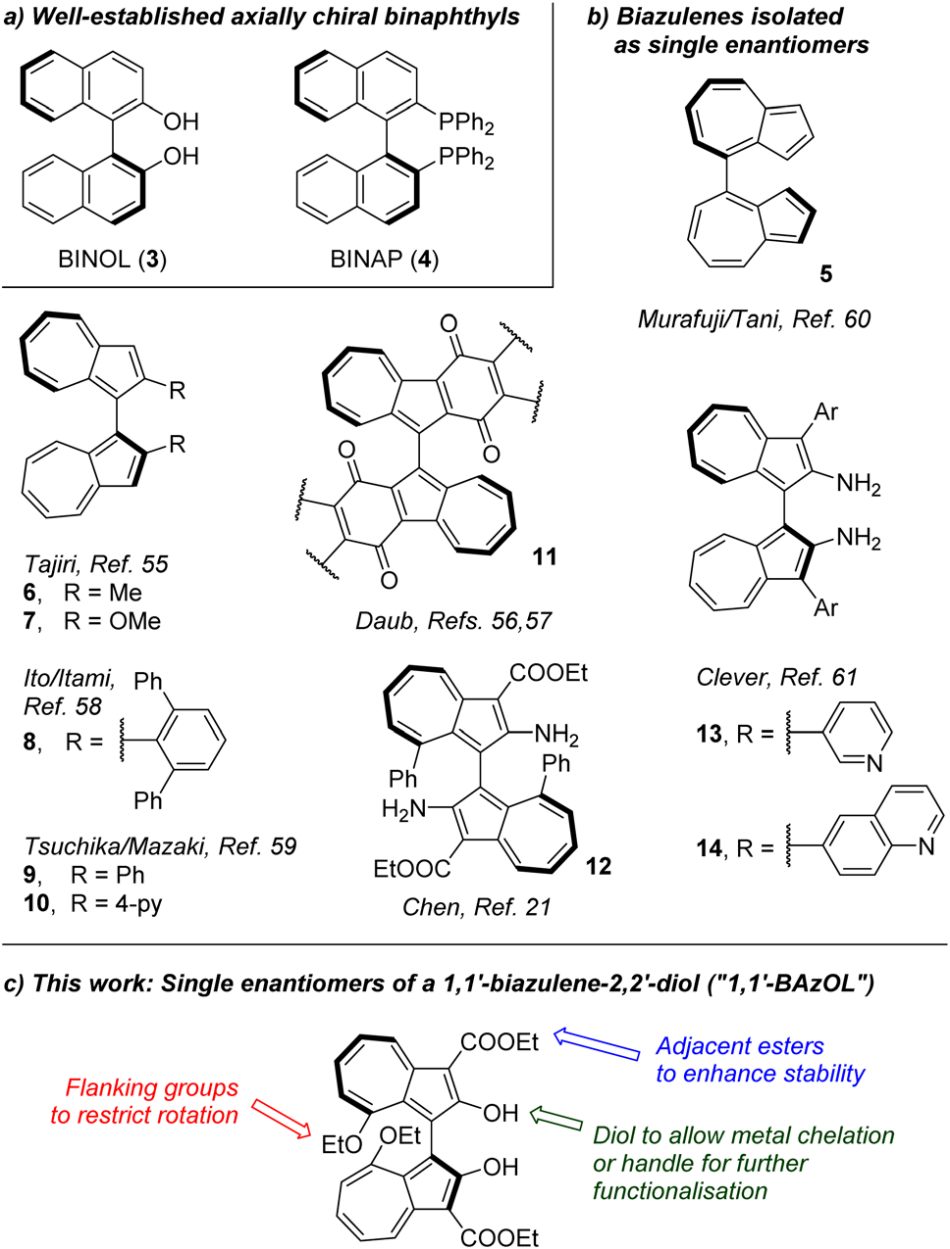
Known binaphthyls (a) and biazulenes (b); design for 1,1′-BAzOL (c).

Axial chirality is a form of stereoisomerism which arises in molecules comprising two pairs of (inequivalent) substituents oriented in a non-planar manner about a chiral axis. Atropisomers exhibit axial chirality arising from restricted rotation around a σ-bond, with the most well-known examples being biaryl systems where the presence of substituents *ortho* to the biaryl bond imposes a steric barrier to racemisation. In particular binaphthyl is a privileged motif in asymmetric catalysis, with the archetypal BINOL (3)^51^ and BINAP (4)^52^ chiral ligands and their derivatives^53^ imparting high levels of enantioselectivity in diverse transition metal-catalysed reactions (Fig. 2a). Chiral Brønsted acid organocatalysts based on the BINOL scaffold are also well developed.^54^

In contrast to binaphthyl, axial chirality in biazulenyl systems has been much less studied. Whereas any biazulene positional isomer could potentially exhibit atropisomerism if appropriately substituted, the few published reports mostly concern 1,1′-biazulenes. In 1983 Tajiri was the first to disclose the resolution of a biazulene, using preparative chiral stationary phase HPLC to separate the enantiomers of 2,2′-dimethyl-1,1′-biazulene 6 and 2,2′-dimethoxy-1,1′-biazulene 7 (Fig. 2b);^55^6 was reported have greater configurational stability than 7. Subsequently Daub studied chiral annulated 1,1′-biazulene quinones 11 as electron-transfer mediators, resolving their enantiomers by HPLC^56^ as well as using a chiral auxiliary to attempt diastereoselective azulene dimerisation, giving the 1,1′-biazulene product in moderate diastereoisomeric excess.^57^ Chen described the synthesis of a 2,2′-diamino-1,1′-biazulene 12, resolution of the racemate by HPLC and attempted enantioselective oxidative dimerisation of the 2-aminoazulene precursor, employing various chiral ligands and achieving modest enantiomeric excess.^21^ Ito, Itami and co-workers reported π-extended 1,1′-biazulenes (8 and its cyclised derivative) which they resolved by HPLC.^58^ Tsuchiya, Mazaki and co-workers reported 2,2′-diphenyl-1,1′-biazulene 9 and 2,2′-bis(4-pyridyl)-1,1′-biazulene 10 and their resolution through crystal picking.^59^ Tani, Murafuji and co-workers reported 4,4′-biazulene 5 and its resolution by HPLC.^60^ Most recently the Clever group reported 2,2′-diamino-3,3′-bis(3-pyridyl)-1,1′-biazulene 13 and 2,2′-diamino-3,3′-bis(6-quinolinyl)-1,1′-biazulene 14, their resolution by HPLC and their chiral self-sorting phenomena in Pd_2_L_4_ coordination cages.^61^ Biazulenes exhibiting helical chirality have also been reported.^62^

Here we report the design, synthesis, resolution and characterisation of a biazulene analogue of BINOL, *i.e.* a 1,1′-biazulene-2,2′-diol, which we have termed “1,1′-BAzOL” (Fig. 2c). Whereas Tajiri had reported 2,2′-dimethoxy-1,1′-biazulene 7, we specifically targeted the free hydroxyl groups to facilitate potential applications of 1,1′-BAzOL, *e.g.* as a chiral ligand or in chiral Brønsted acid catalysis. Our design incorporated two further motifs with specific functions. Firstly, we introduced “flanking” groups at the 8- and 8′-positions, intended to increase the barrier to racemisation. Secondly, we appended ester groups at the 3- and 3′-positions, anticipating that these would enhance the chemical stability of 1,1′-BAzOL. 2-Hydroxyazulene is only moderately stable in solution since the substituent renders the azulene core sufficiently electron-rich that it may undergo oxidative degradation. Furthermore, depending on the solvent, 2-hydroxyazulene can tautomerise to the corresponding keto-form to an appreciable degree.^63^ This may then undergo aldol-type self-condensation reactions, ultimately leading to decomposition, and we were mindful that this decomposition pathway might also be operative for a 1,1′-biazulene-2,2′-diol. However, 2-hydroxyazulenes bearing an electron-withdrawing ester group at the adjacent position are less electron-rich and generally stable, with the tautomeric equilibrium seemingly favouring the enol form to a much greater degree. We therefore sought to introduce ester groups at the BAzOL 3- and 3′-positions, in the hope this would suppress decomposition *via* the keto tautomer. The realisation of our BAzOL design concept is reported in this paper. Of note, a 1,1′-biazulene-2,2′-diol has never been isolated in enantiopure form. Chen and co-workers prepared a 1,1′-biazulene-2,2′-diol by electrochemical oxidative dimerisation, but chirality was not considered.^64^ Yang, Nozoe and co-workers prepared a 1,1′-biazulene crown ether, in which the chirality of the system was recognised, but resolution was not attempted.^65^

## Results and discussion

**Scheme 1:**
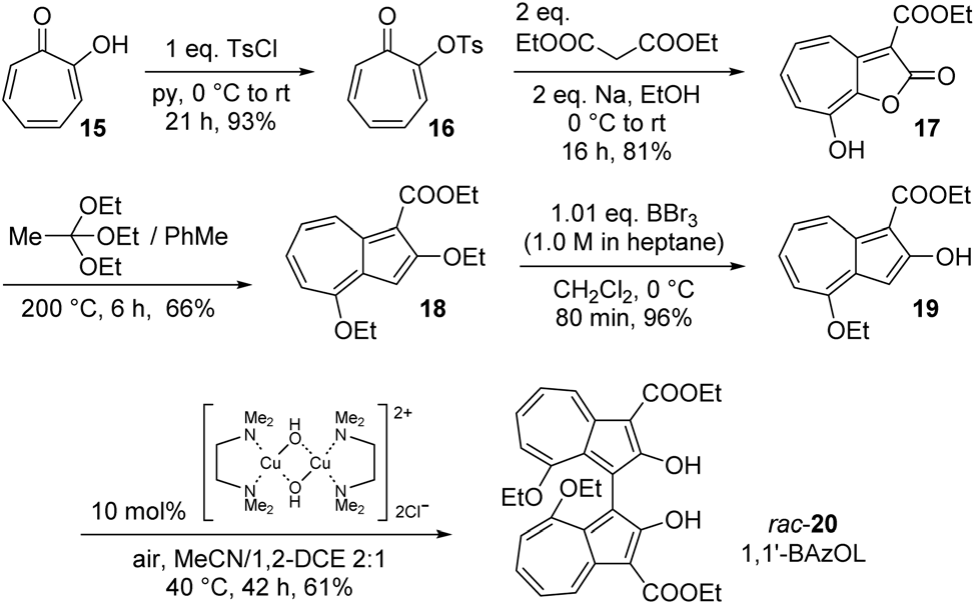
Synthesis of *rac*-1,1′-BAzOL 20.

The synthesis of 1,1′-BAzOL began with commercially available tropolone 15, which was tosylated to give 16, then reacted with ethyl cyanoacetate to give bicyclic hydroxylactone 17, as described previously (Scheme 1).^66^ Heating of 17 with triethyl orthoacetate in a sealed tube gave bis(ethoxy)azulene 18. The reaction proceeds by *in situ* generation of a ketene acetal which undergoes an [8 + 2] cycloaddition with 17, followed by extrusion of CO_2_ to give 18.^67^ Deethylation with boron tribromide proceeded entirely regioselectively to give 19, which was of sufficient purity to be used in the next step without purification. The 8-ethoxy group was inert under these reaction conditions since this ether oxygen is less Lewis basic, being attached to the more electron-poor position on azulene 18. Then, oxidative dimerisation of 19 was effected using [Cu(OH)(TMEDA)]_2_Cl_2_ under air, which has previously been reported to be an effective catalyst system for dimerisation of 2-naphthols to BINOLs.^68^ In this case, the reaction gave *rac*-1,1′-BAzOL 20 in 62% yield (5 step synthesis from tropolone, 29% overall yield).

**Scheme 2:**
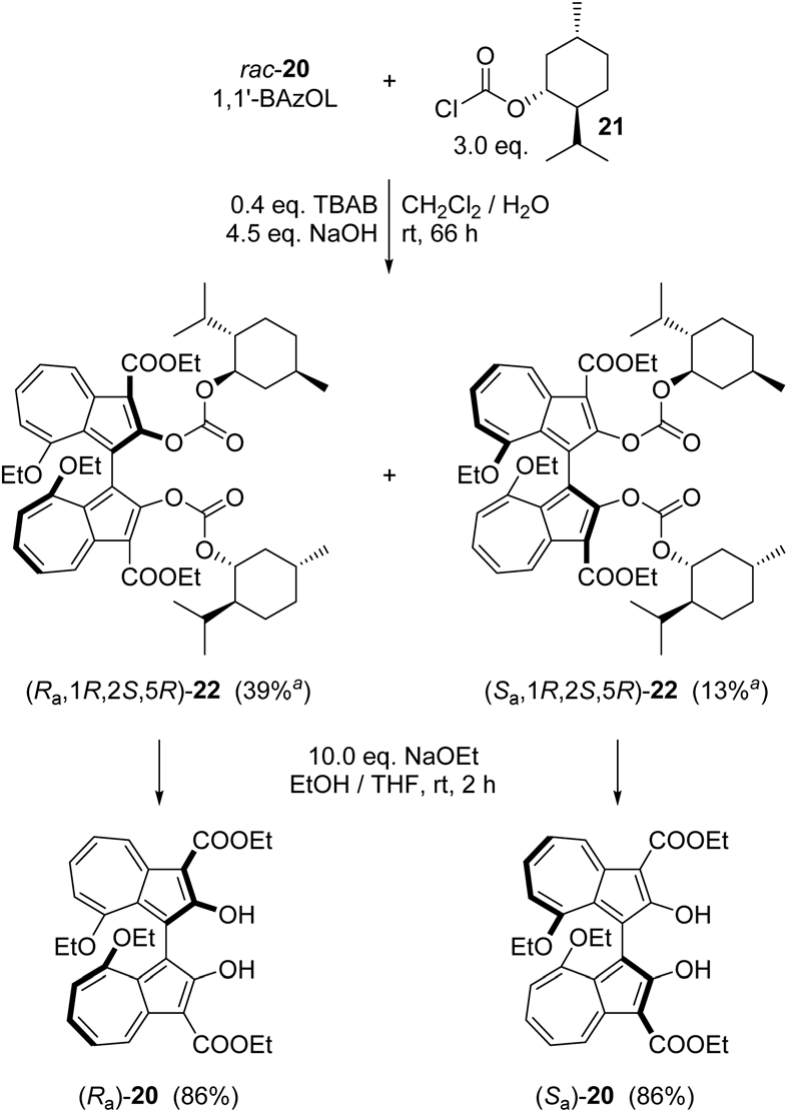
Resolution of 1,1′-BAzOL 20 by formation of bis(menthyl carbonate) derivatives, separation and ethanolysis. ^a^Isolated yield of pure material with respect to the theoretical maximum of that diastereoisomer.

To isolate 1,1′-BAzOL 20 in enantiopure form, we attempted to develop an enantioselective variant of the dimerisation of 19. A procedure reported for enantioselective dimerisation of 2-naphthols using Cu-BINAM complexes^69^ was adapted for reaction of 19, but 1,1′-BAzOL 20 was obtained in only low *e.e.*, and in low yield, with various copper sources. We therefore sought to resolve *rac*-1,1′-BAzOL 20 instead, through derivatisation with a chiral pool-derived auxiliary and separation of the resultant diastereoisomers. Commercially-available (−)-menthyl chloroformate 21 has previously been used successfully for the derivatisation and separation of enantiomers of BINOL and related chiral diols,^70^ and we applied this approach to 1,1′-BAzOL (Scheme 2). Thus, reaction of an excess of 21 with *rac*-1,1′-BAzOL 20 in a biphasic dichloromethane–water medium, in the presence of TBAB (tetra-*n*-butylammonium bromide) as phase-transfer catalyst and NaOH as base gave bis(menthyl carbonate) 22 as a 1 : 1 mixture of diastereoisomers. In the original reports on the resolution of BINOL by this method, fractional crystallisation of the diastereoisomeric mixture afforded one diastereoisomer (100% *d.e.*) in pure crystalline form, whereas the motherliquor contained the other diastereoisomer in ≈90% *d.e.*, that could be further purified to higher *d.e.* through subsequent operations. In the case of 1,1′-BAzOL, the bis(carbonate) 22 derived from (*R*_a_)-1,1′-BAzOL 20 could indeed be isolated as a single diastereoisomer through careful crystallisation, albeit in more moderate yield. The motherliquor was concentrated and then underwent further recrystallisations from a different solvent, giving the bis(carbonate) 22 derived from (*S*_a_)-1,1′-BAzOL 20 as a single diastereoisomer, in low yield. (Subsequent chromatography and recrystallisation provided additional material; see ESI† for details) Both of the diastereoisomers of 22 isolated in this way were then separately subjected to ethanolysis to cleave the menthol auxiliary and regenerate 1,1-BAzOL 20. As shown in Scheme 2, this was achieved in the same high yield for both diastereoisomers of 22, thus allowing the isolation of both enantiomers of 1,1′-BAzOL 20 in enantiopure form.

**Fig. 3:**
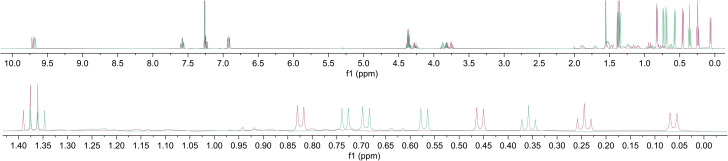
Overlaid ^1^H-NMR Spectra (in CDCl_3_) of the separated diastereoisomers of BAzOL-bis(menthyl carbonate) 22: (*R*_a_) isomer (purple) and (*S*_a_) isomer (green).

**Fig. 4:**
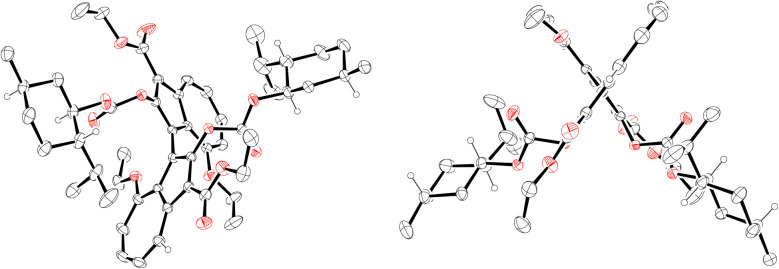
ORTEP representations of the X-ray structure of (*R*_a_,1*R*,2*S*,5*R*)-22. Ellipsoids are shown at 30% probability. A molecule of ethanol has been omitted for clarity. Only hydrogens on stereogenic centres are shown (as spheres of arbitrary radius). CCDC #2421193.

**Fig. 5:**
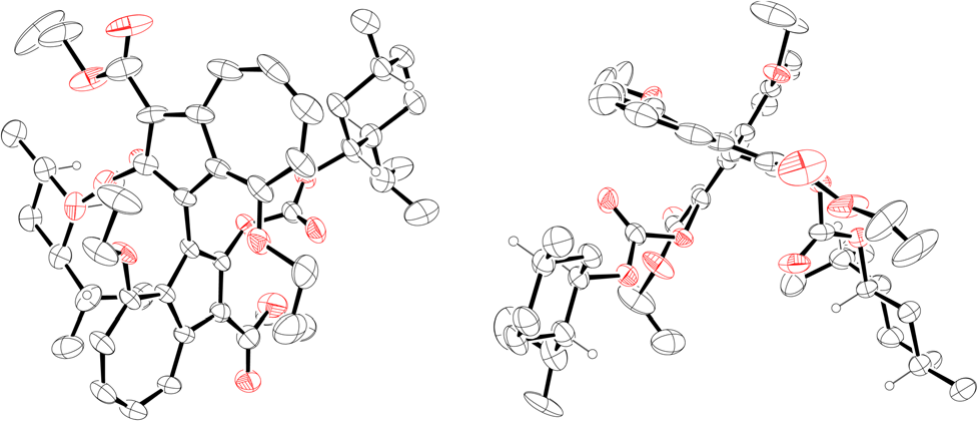
ORTEP representations of the X-ray structure of (*S*_a_,1*R*,2*S*,5*R*)-22. Ellipsoids are shown at 30% probability. Disorder in the ethyl esters and menthyl isopropyl group has been omitted for clarity. Only hydrogens on stereogenic centres are shown (as spheres of arbitrary radius). CCDC #2421194.

**Fig. 6:**
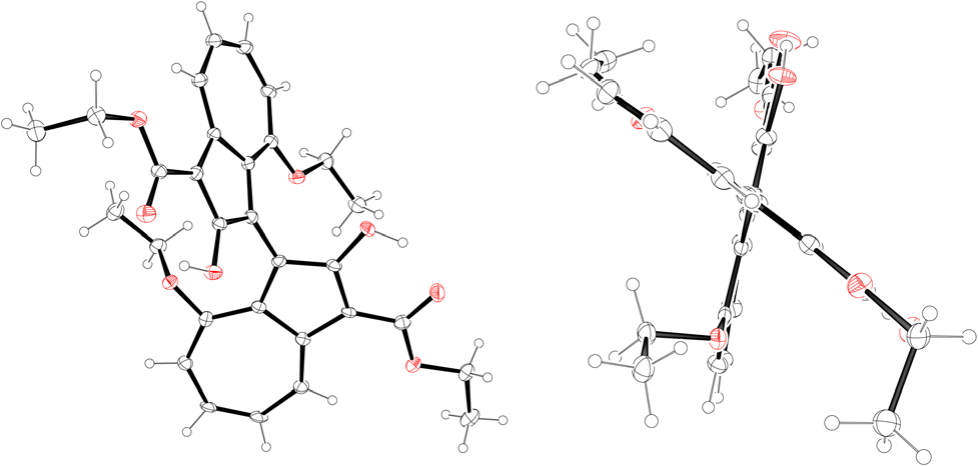
ORTEP representations of the X-ray structure of 1,1′-BAzOL (*R*_a_)-20. Ellipsoids are shown at 50% probability. Hydrogens are shown as spheres of arbitrary radius. CCDC #2421195.

The ^1^H-NMR spectra for the two diastereoisomers of 22 are very similar in the aromatic region, but exhibit significant chemical shift differences in the upfield region (Fig. 3). Thus, the methyl groups of the menthyl auxiliary are clearly discernible as three doublets between 0 and 1 ppm (since each isopropyl group comprises two inequivalent methyl groups). We ascribe the chemical shift differences between the two isomers for these signals to differing degrees of anisotropic shielding by the azulene seven-membered rings. Further structural information for the diastereoisomers of 22 was obtained through X-ray crystallography, with the structures so acquired shown in Fig. 4 (for the (*R*_a_) diastereoisomer) and Fig. 5 (for the (*S*_a_) diastereoisomer). Additionally, an X-ray crystal structure for (*R*_a_)-1,1′-BAzOL 20 itself was also acquired (Fig. 6). Selected crystallographic parameters are shown in Table 1.

**Table 1:** Selected bond lengths and angles

Structure	C1-C1′ biaryl bond length (Å)	C2-C1-C1′-C2′ dihedral angle (°)
(*R*_a_,1*R*,2*S*,5*R*)-22	1.476(2)	71.4(2)
(*S*_a_,1*R*,2*S*,5*R*)-22	1.450(8)	99.0(8)
1,1′-BAzOL (*R*_a_)-20	1.460(7)	111.8(6)

**Fig. 7:**
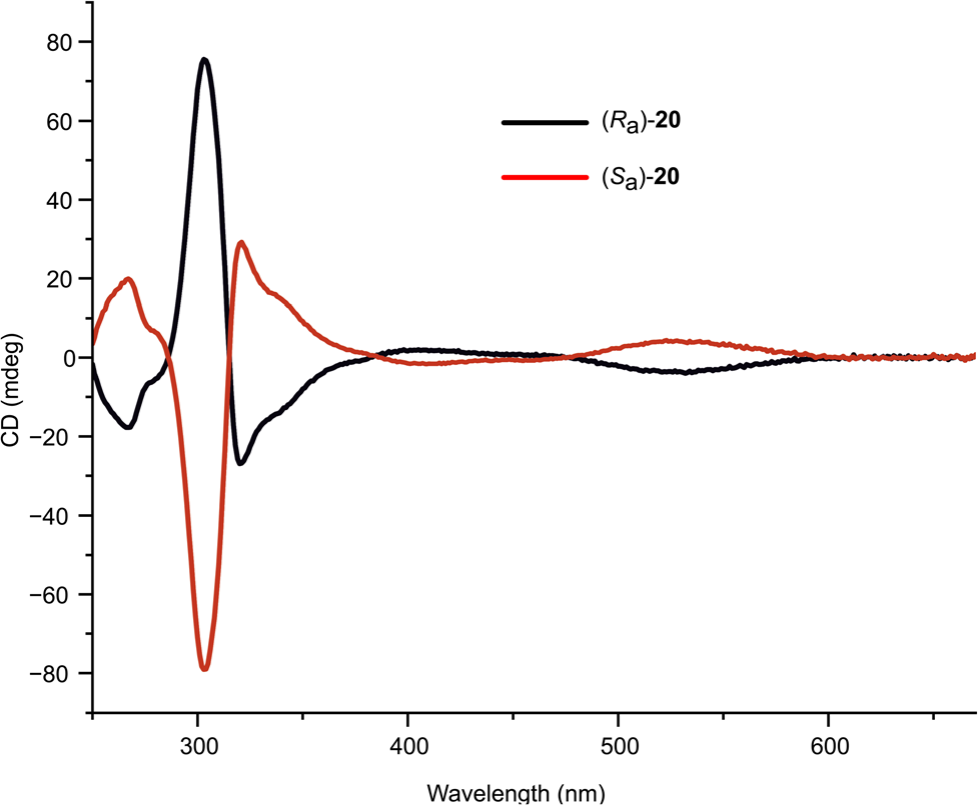
Circular dichroism plots of (*R*_a_)-20 (black) and (*S*_a_)-20 (red), recorded as 0.01 mM solutions in CHCl_3._

**Fig. 8:**
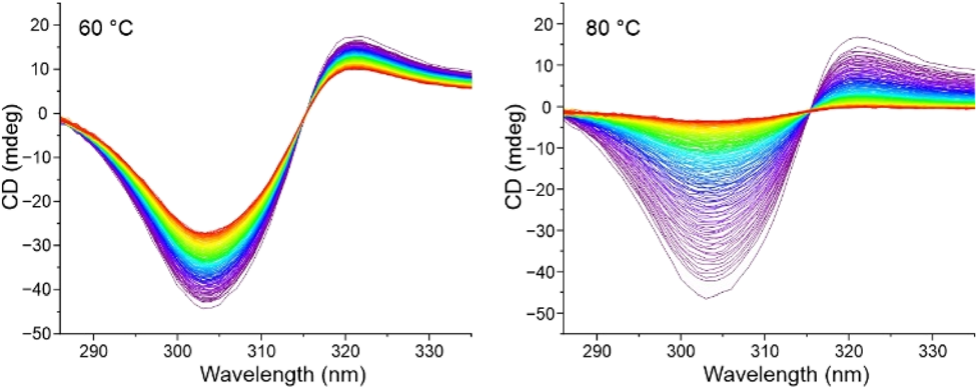
Circular dichroism plots of (*S*_a_)-20, recorded as 0.01 mM solutions in 1,1,2,2-tetrachloroethane. Spectra recorded at 5 minutes intervals (left) at 60 °C; (right) at 80 °C.

Circular dichroism spectra for the enantiomers of 1,1′-BAzOL 20 were acquired and are shown in Fig. 7. The superimposable mirror image spectra indicate that 1,1′-BAzOL 20 is configurationally stable at room temperature and confirm the enantiopurity. The configurational stability was then investigated at elevated temperatures. As shown in Fig. 8, partial racemisation was observed when a solution of (*S*_a_)-1,1′-BAzOL 20 was maintained at 60 °C for 14 h, whereas near-complete racemisation was observed at 80 °C for the same period. Using data acquired at these and other temperatures, the barrier to racemisation was calculated (see ESI† for details). The key parameters are summarised in Table 2.

**Table 2:** Key thermodynamic parameters for racemisation of 1,1′-BAzOL 20

*E*_a_/kJ mol^−1^	Δ*H*^‡^/kJ mol^−1^	Δ*S*^‡^/J mol^−1^ K^−1^	Δ*G*^‡^_293.15 K_/kJ mol^−1^	*t*_1/2 293.15 K_/h
84.90	82.06	−96.06	110.2	1389

Table 3 presents a comparison of the barrier to racemisation determined for 1,1′-BAzOL 20 with all other biazulenes for which data on racemisation have been reported. The measured *E*_a_ value for 1,1′-BAzOL 20 is similar to that for 4,4′-biazulene 5. The value for 2,2′-dimethoxy-1,1′-biazulene 7 is appreciably lower than for 20, which may be due to the fact that 7 lacks the flanking groups in the 8,8′-positions. On the other hand, the values for 9 and 10 are appreciably higher than the value we have measured for 20, implying that a sufficiently bulky group at C2 can hinder rotation around the biaryl axis regardless of the presence or absence of flanking groups on the seven-membered rings. The *E*_a_ value for the racemisation of 1,1′-binaphthyl 23 has also been included for comparison; it is higher than for 20 but lower than for 9 and 10.

**Table 3:** Barriers to racemisation for various biaryls

Biaryl	*E*_a_/kJ mol^−1^	Biaryl	*E*_a_/kJ mol^−1^
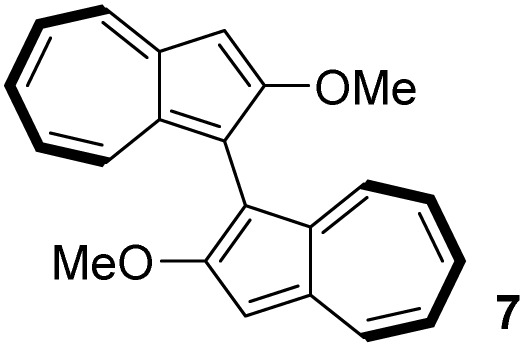	71 (ref. 55)	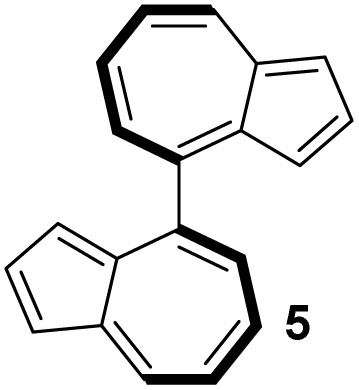	87.9^a^ (ref. 60)
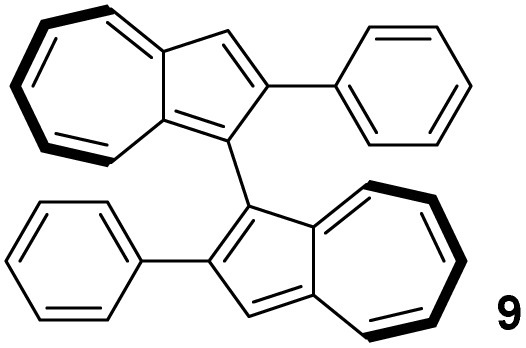	108.9^a^ (ref. 59)	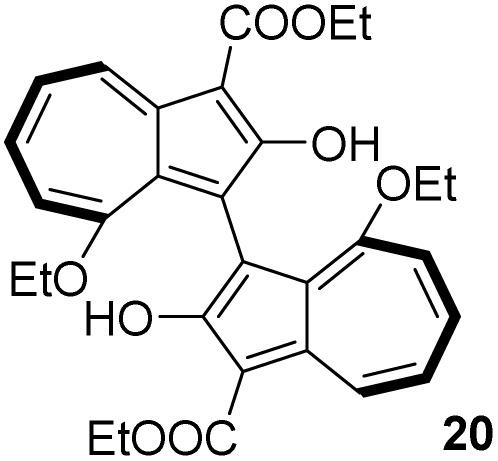	84.9 (this work)
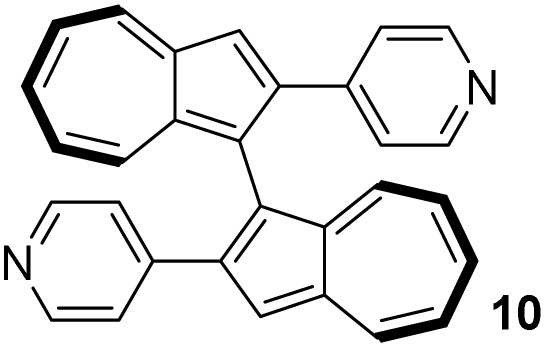	106.6^a^ (ref. 59)	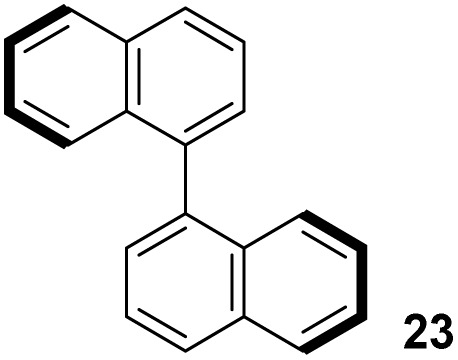	94.1 (ref. 71)

a These values are not reported directly in the references cited. Rather, we have calculated these values using the data presented in these literature sources.

**Scheme 3:**
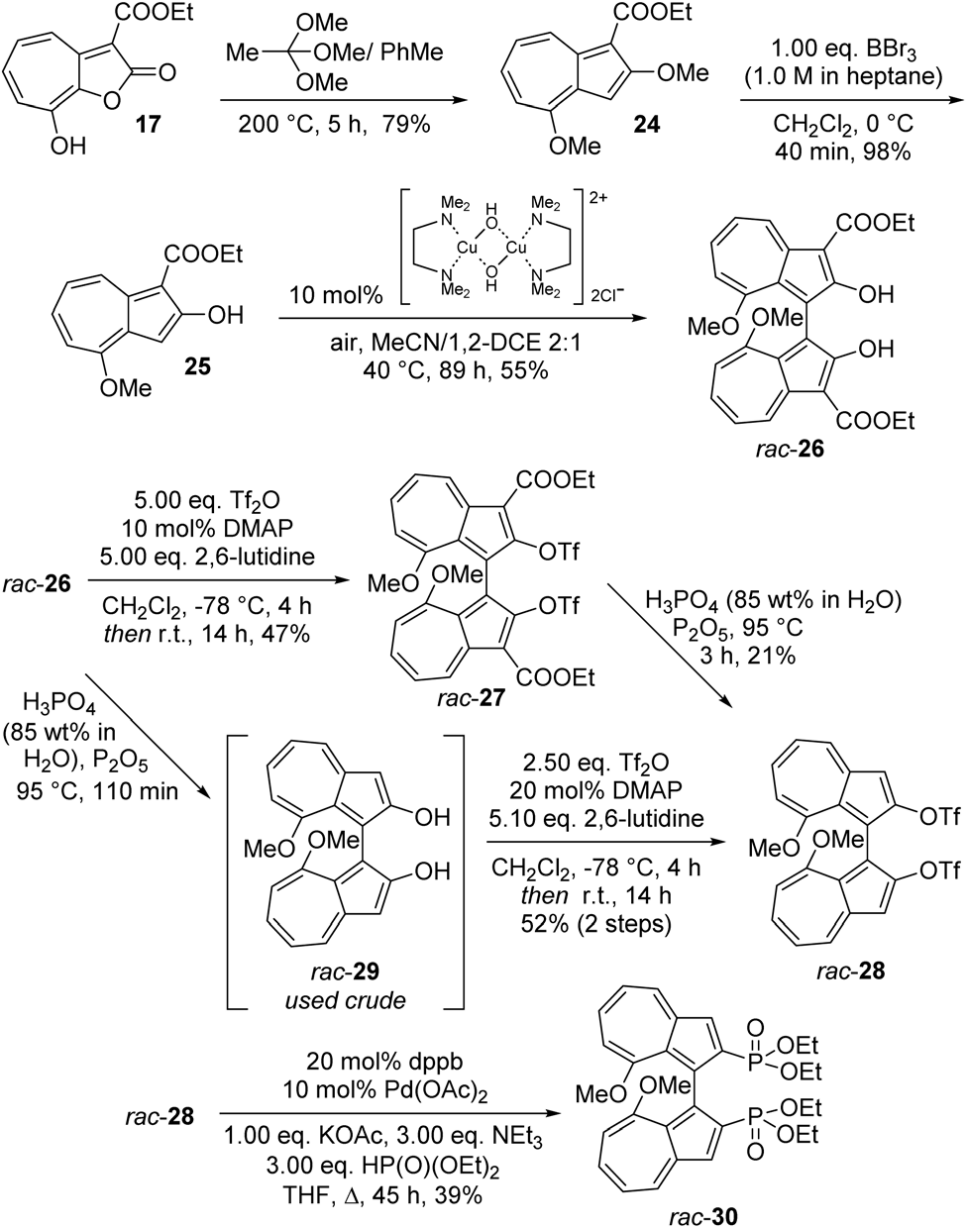
Synthesis of bis(phosphonate) *rac*-30.

We next sought to apply the biazulene synthesis we had developed to produce a 1,1′-biazulene bearing different functional groups, through derivatisation of the diol. To this end, lactone 17 was reacted with trimethyl orthoacetate to give dimethoxyazulene 24 (Scheme 3), this reaction proceeding in higher yield (79%) than for the diethoxy homologue 18 (66%, see Scheme 1). Dealkylation was again selective for the 2-position, giving hydroxyazulene 25, which underwent oxidative dimerisation to give *rac*-26. At this point, we sought to convert this biazulene diol into the corresponding bis(triflate) in order to be able to derivatise it by cross-coupling approaches. In the event, the potential cross-coupling partner 27 was formed in moderate yield upon use of excess triflic anhydride. At this point we considered the necessity of 3,3′-diester substituents in the present synthesis. As explained above, they were considered essential in the 1,1′-BAzOL design strategy in order to impart stability by suppressing keto–enol tautomerism and also to block over-oxidation/oligomerisation in the dimerisation of 19 to 1,1′-BAzOL 20. However, in 27 the triflate groups are non-enolisable (and less electron-rich), so we reasoned the ester functionalities could be considered to have served their purpose at this point in the synthetic sequence. As such, we aimed to demonstrate their removal upon treatment with phosphoric acid, which is often employed for hydrolysis/decarboxylation of esters at the azulene 1- and 3-positions.^23,72^ In this case, treatment of 27 with H_3_PO_4_/P_2_O_5_ gave expected bis(triflate) 28 only in low yield. Much more satisfactory was reversing the order of events, with acid-mediated ester removal from 26 giving 29, which if used immediately (and without purification) could be doubly sulfonylated to give 28 in a greatly improved 52% yield over two steps. Then a representative twofold cross-coupling was demonstrated for 28, with the synthesis of 2,2′-bis(phosphonate)-1,1′-biazulene 30 according to the method of Stawinski *et al.*^73^ (Subsequently, an attempt to couple 27 under the same conditions gave only the mono-coupled product).

## Conclusions

We have prepared an axially chiral 1,1′-biazulenyl-2,2′-diol in enantiopure form and determined the barrier to its racemisation. The synthetic access to 1,1′-BAzOL 20 is concise (5 steps from commercial materials to the racemate; 7 steps to the single enantiomers) and there is scope for diversification of the substituents. We have demonstrated this by carrying out an exemplary cross-coupling using a variant of 20 – transformation to bis(triflate) 28 gave a suitable electrophilic coupling partner, which underwent a double cross-coupling to give 2,2′-bis(phosphonate) 30. Analogous cross-couplings to introduce many other substituents or functional groups at the 2-positions may be envisaged. In addition, functionalisation at the 3-positions should be possible either by functional group interconversions of the esters, or by their removal (as per the transformation of 27 to 28) followed by electrophilic aromatic substitution (since the unsubstituted 3-position may be anticipated to be the most reactive for S_E_Ar). For these reasons we anticipate that the BAzOL synthesis described here may find varied applications in synthesis and catalysis.

## Data availability

The data supporting this article have been included as part of the ESI.† Crystallographic data for both diastereoisomers of 22 and for 1,1′-BAzOL (*R*_a_)-20 have been deposited at the CCDC under #2421193–2421195 and can be obtained free of charge at https://www.ccdc.cam.ac.uk/structures.

## Author contributions

S. E. L. conceived the project. A. P. G. carried out all synthetic work. T. M. G. and G. D. P. carried out circular dichroism analysis. G. K. K. carried out X-ray crystallography. S. E. L. wrote the manuscript with input from all authors.

## Conflicts of interest

There are no conflicts to declare.

## Supplementary Material

RA-015-D5RA02520F-s001

RA-015-D5RA02520F-s002
