# Supplementary material for: Synthesis and resolution of a 1,1′-biazulene analogue of BINOL
Source: RSC Adv. 2025 May 16;15(19):14881–92. doi: 10.1039/d5ra02520f (PMC12082637; doi:10.1039/d5ra02520f)
Supplement: RA-015-D5RA02520F-s001 [file RA-015-D5RA02520F-s001.pdf]

Supplementary Information (SI) for RSC Advances.
This journal is © The Royal Society of Chemistry 2025

Synthesis and resolution of a 1,1'-biazulene analogue of BINOL.

Anthony P. Gee,[a,b] Tiberiu-M. Gianga,[a,c] Gabriele Kociok-Köhn,[d] G. Dan Pantoş,[a] and Simon E. Lewis*[a,e]

a. Department of Chemistry, University of Bath, Bath, BA2 7AY, U.K. E mail: S.E.Lewis@bath.ac.uk
b. Present address: Evotec (UK) Ltd., 95 Park Drive, Abingdon, OX14 4RY, U.K.
c. Present address: B23 Beamline, Diamond Light Source, Didcot OX11 0DE, U.K.
d. Physical Structure Characterization Facility, University of Bath, Bath, BA2 7AY, U.K.
e. Institute of Sustainability and Climate Change, University of Bath, Bath, BA2 7AY, U.K.

ELECTRONIC SUPPORTING INFORMATION

General Experimental Details — Page S2

Synthetic procedures — Page S3

Calculation of barrier to racemisation of 1,1'-BazOL **20** — Page S21

NMR spectra — Page S28

Crystallographic Data — Page S58

References — Page S61

General Experimental Details

All reactions were carried out under an atmosphere of N_2 or argon, through the use of a Schlenk line, unless otherwise stated. Anhydrous solvents were either purchased from Fisher Scientific or Sigma-Aldrich, or purified through anhydrous alumina columns using an Innovative Technology Inc. PS-400-7 solvent purification system, or purified by distillation (over sodium/benzophenone ketyl or calcium hydride). Solvents were deoxygenated either by channelling a stream of N_2 through the liquid (sparging), or by the freeze-thaw-pump method. Thin layer chromatography (TLC) was carried out on aluminium plates coated with silica gel (Alugram®SIL G/UV $_{254\ nm}$), and visualisation was achieved with UV light or $KMnO_4$, ceric ammonium molybdate and iodine dips, followed by gentle heating. Solvents were removed using Büchi rotary evaporators and with high vacuum on a Schlenk line. Flash column chromatography was carried out using Davisil LC 60 Å silica gel (35-70 micron) purchased from Sigma-Aldrich.

NMR spectra were run in $CDCl_3$ unless otherwise stated, on Bruker Avance 250, Bruker Avance 300, Bruker Avance 400, Bruker Avance 500 II+ or Agilent A500a instruments. IR spectra were recorded on a Perkin-Elmer 1600 FT-IR instrument. Capillary melting points were recorded on a Büchi 535 melting point apparatus, and are uncorrected. High resolution mass spectrometry (HRMS) was carried out using a micrOTOF ESI-TOF spectrometer coupled to an Agilent 1200 LC system for auto-sampling. For SCXRD the selected crystal was mounted onto a goniometer head and cooled to 150K with an Oxford Cryosystem. Intensity data for compounds (R_a,1*R*,2*S*,5*R*)-**22,** (S_a,1*R*,2*S*,5*R*)-**22** and (R_a)-**20** were collected on a SuperNova, Dual, Cu at zero, EosS2 using a Cu microfocus source (λ = 1.54184) Å. Unit cell determination, data collection, data reduction and a symmetry-related (multi-scan) absorption correction were performed using the CrysAlisPro software.[1] The structure was solved with SHELXT and refined by a full-matrix least-squares procedure based on F^2 (Shelxl-2019/2).[2] All non-hydrogen atoms were refined anisotropically. Hydrogen atoms were placed onto calculated positions and refined using a riding model. Additional programmes used for analysing data and their graphical manipulation included: SHELXle[3] and Mercury.[4]

7-Oxocyclohepta-1,3,5-trien-1-yl 4-methylbenzene-1-sulfonate 16

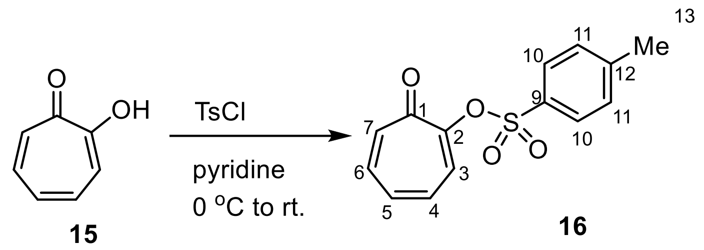

The preparation of this compound was based on the procedure of Hiskey and von Eggers Doering.[5] At 0 °C, to a stirred mixture of tropolone **15** (12.2 g, 100 mmol, 1.00 eq.) and *p*-toluenesulfonyl chloride (19.1 g, 100 mmol, 1.00 eq.) was slowly added pyridine (30 mL), and then was allowed to warm to r.t. The mixture was stirred for 3 h, forming a viscous consistency after 1 h. To the mixture was added water (120 mL), stirred for 15 min and the precipitate was collected by filtration to give *7-oxocyclohepta-1,3,5-trien-1-yl 4-methylbenzene-1-sulfonate* **16** (25.7 g, 93.1 mmol, 93%) as an off-white solid, used without further purification; R_f 0.60 (EtOAc); δ_H (250 MHz, $CDCl_3$) 7.92 (2H, d, *J* 8.5 Hz, 10-CH), 7.46 (1H, dd, *J* 9.0 Hz, 1.0 Hz, 7-CH), 7.37-7.33 (2H, dd, *J* 8.5 Hz, 1.0 Hz, 11-CH), 7.24-6.95 (4H, m, 3,4,5,6-CH), 2.45 (3H, s, 15-CH_3).

Data in agreement with those previously reported.[6]

Ethyl 8-hydroxy-2-oxo-*2H*-cyclohepta[*b*]furan-3-carboxylate 17

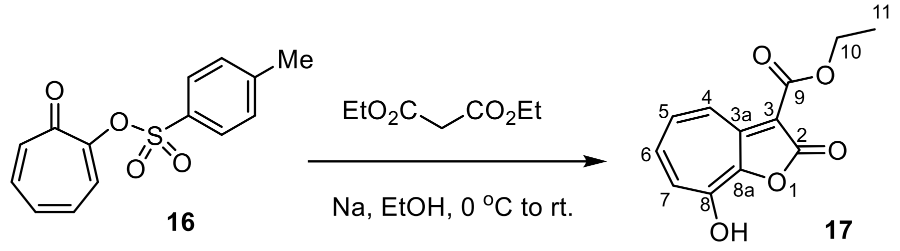

The preparation of this compound was based on method by Nozoe.[7] At 0 °C, to a stirred suspension of 7-oxocyclohepta-1,3,5-trien-1-yl 4-methylbenzene-1-sulfonate **16** (13.8 g, 50.0 mmol, 1.00 eq.) and diethyl malonate (14.0 mL, 100.0 mmol, 2.00 eq.) in ethanol (100 mL) was added by cannula NaOEt solution (freshly prepared from Na metal (2.30 g, 100.0 mmol, 2.00 eq.) and ethanol (100 mL)). The mixture was allowed to warm to r.t. in the ice bath, while the ice melted, forming a gel-like consistency after 10 min. After 16 h, to the mixture was added water (250 mL), and the solution was extracted with DCM (3 × 100 mL). The organic extracts were discarded, and to the aqueous layer was added $HCl_{(aq)}$ (5 M, 150 mL), which changed from an orange/brown solution to a bright yellow suspension. After chilling at 2 °C for 2 h, the mixture was filtered to give *ethyl 8-hydroxy-2-oxo-*2H*-cyclohepta[*b*]furan-3-carboxylate* **17** (9.48 g, 40.5 mmol, 81%) as a bright yellow fluffy solid; R_f 0.00 (EtOAc); δ_H (250 MHz, DMSO-d_6) 8.76 (1H, d, *J* 10.5 Hz, 4-CH), 7.70-7.47 (3H, m, 5,6,7-CH), 4.23 (2H, q, *J* 7.0 Hz, 10-CH_2), 1.28 (3H, t, *J* 7.0 Hz, 11-CH_3).

Data in agreement with those previously reported.[7]

Ethyl 2,4-diethoxyazulene-1-carboxylate 18

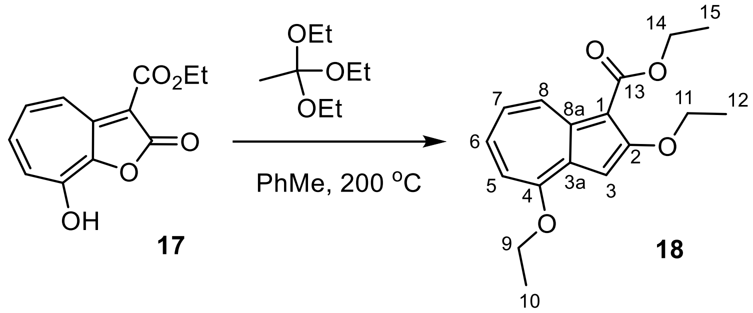

The preparation of this compound was based on a method by Pham.[8] Under atmosphere of air, a high-pressure round-bottom flask (capacity 50 mL) was charged with 8-hydroxy-2-oxo-*2H*-cyclohepta[*b*]furan-3-carboxylate **17** (3.00 g, 12.8 mmol), triethyl orthoacetate (9.0 mL) and toluene (6.0 mL) and sealed, then heated under air at 200 °C, stirring for 6 h (CAUTION: the reaction was run behind a blast shield). After cooling to r.t., the resultant deep red solution was loaded onto a silica column, and purified by column chromatography (5→25% EtOAc in petroleum ether) to give *ethyl 2,4-diethoxyazulene-1-carboxylate* **18** (2.43 g, 8.42 mmol, 66%) as a red crystalline solid; R_f 0.45 (1:1 petroleum ether/EtOAc); δ_H (250 MHz, $CDCl_3$) 9.38 (1H, d, *J* 10.0 Hz, 8-CH), 7.40 (1H, ddd, *J* 11.0 Hz, 10.0 Hz, 1.0 Hz, 6-CH), 7.22 (1H, td, *J* 10.0 Hz, 1.0 Hz, 7-CH), 6.98 (1H, d, *J* 10.5 Hz, 5-CH), 6.93 (1H, s, 3-CH), 4.42 (2H, q, *J* 7.0 Hz, CH_2), 4.33 (2H, q, *J* 7.0 Hz, CH_2), 4.21 (2H, q, *J* 7.0 Hz, CH_2), 1.54 (3H, t, *J* 7.0 Hz, CH_3), 1.51 (3H, t, *J* 7.0 Hz, CH_3), 1.44 (3H, t, *J* 7.0 Hz, CH_3).

Data in agreement with those previously reported.[9]

Ethyl 4-ethoxy-2-hydroxyazulene-1-carboxylate 19

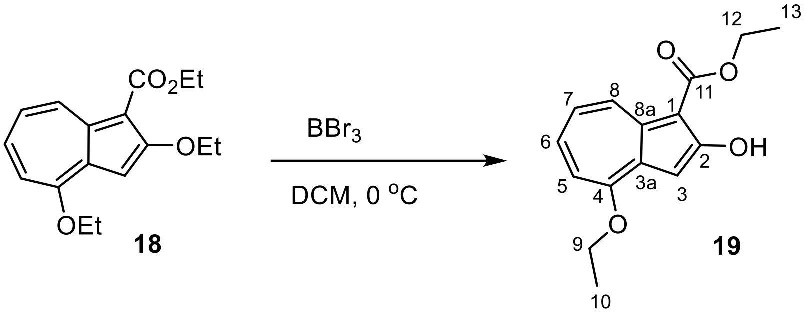

To a stirred solution of ethyl 2,4-diethoxyazulene-1-carboxylate **18** (2.43 g, 8.43 mmol, 1.00 eq.) in DCM (80 mL), at 0 °C, was slowly added BBr_3 (1.0 M in heptane, 8.50 mL, 1.01 eq.), instantly forming a yellow/brown solution. The mixture was allowed to stir for 80 min, then to it was added MeOH (10 mL), forming an orange solution. The solution was diluted with DCM (80 mL) and washed with water (2 × 60 mL). After drying with anhydrous $MgSO_4$, the organic phase was filtered. The filtrate was concentrated under reduced pressure to give pure *ethyl 4-ethoxy-2-hydroxyazulene-1-carboxylate* **19** (2.10 g, 8.08 mmol, 96%) as an orange/brown solid, which was used without further purification (m.p. 107-110 °C); R_f 0.45 (3:1 petroleum ether/EtOAc); δ_H (300 MHz, $CDCl_3$) 10.70 (1H, br s, 2-OH), 8.90 (1H, d, *J* 9.5 Hz, 8-CH), 7.53 (1H, td, *J* 10.5 Hz, 1.0 Hz, 6-CH), 7.22 (1H, t, *J* 10.0 Hz, 7-CH), 7.02 (1H, d, *J* 10.6 Hz, 5-CH), 6.98 (1H, s, 3-CH), 4.51 (2H, q, *J* 7.0 Hz, 12-CH_2), 4.29 (2H, q, *J* 7.0 Hz, 9-CH_2), 1.56 (3H, t, *J* 7.0 Hz, 10-CH_3), 1.50 (3H, t, *J* 7.0 Hz, 13-CH_3); δ_C (75 MHz, $CDCl_3$) 169.6 (2-C), 168.8 (11-C), 160.4 (4-C), 137.0 (8a-C), 134.1 (3a-C), 133.0 (8-C), 132.3 (6-C), 123.7 (7-C), 111.7 (5-C), 101.6 (3-C), 98.8 (1-C), 64.8 (9-C), 60.2 (12-C), 14.8 (10-C), 14.6 (13-C); ν_{max} (film) 2986, 2933, 2894, 1628, 1596, 1532, 1478, 1454, 1441, 1421, 1385, 1356, 1316, 1264, 1221, 1200, 1135, 1082, 1067, 1004, 993, 959, 925, 894, 836, 827, 785, 741, 729, 667 cm^{-1}; HRMS (ESI+) *m/z* calc for $[C_{15}H_{16}O_4 + Na]^+$, 283.0946; found, 283.0946.

(±)-3,3'-Diethyl 2,2'-dihydroxy-8,8'-diethoxy-[1,1'-biazulene]-3,3'-dicarboxylate 20

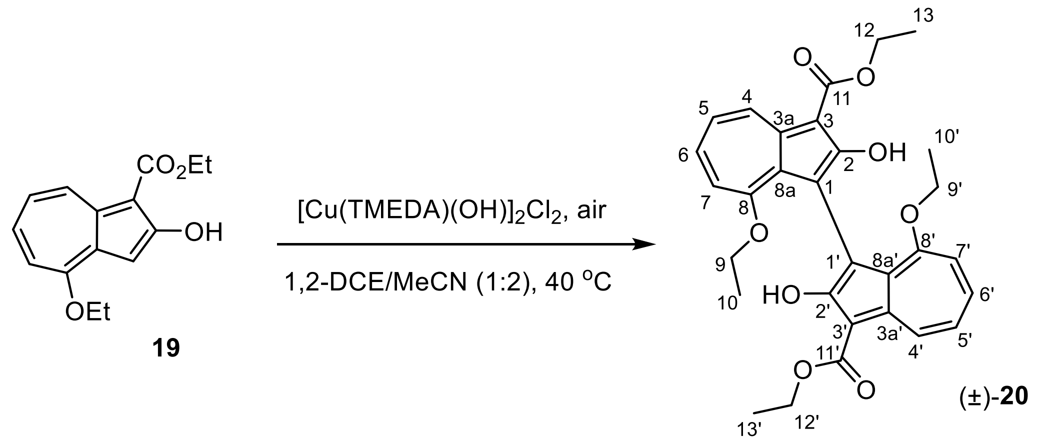

Under atmosphere of air, to a stirred solution of ethyl 4-ethoxy-2-hydroxyazulene-1-carboxylate **19** (2.01 g, 8.06 mmol, 1.00 eq.) in acetonitrile (40 mL) and 1,2-dichloroethane (20 mL) was added di-µ-hydroxo-bis[(*N*,*N*,*N′*,*N′*-tetramethylethylenediamine)copper(II)] chloride (187 mg, 0.806 mmol (by mol. weight of monomer), 0.10 eq.). The mixture was stirred at 40 °C for 42 h, allowed to cool to r.t., and then diluted with DCM (100 mL). The solution was washed with $HCl_{(aq)}$ (0.5 M, 70 mL) and with water (2 × 60 mL). The organic layer was then dried over anhydrous $MgSO_4$, and filtered. The filtrate was concentrated under reduced pressure to give the crude product, which was then purified by column chromatography (20→100% EtOAc in petroleum ether, then 5% MeOH in EtOAc) to give *(±)-3,3'-diethyl 2,2'-dihydroxy-8,8'-diethoxy-[1,1'-biazulene]-3,3'-dicarboxylate* **20** (1.27 g, 2.44 mmol, 61%) as a red solid (m.p. 210-212 °C); R_f 0.54 (1:1 petroleum ether/EtOAc); δ_H (500 MHz, $CDCl_3$) 10.74 (2H, br s, 2,2'-OH), 9.02 (2H, dd, *J* 9.7 Hz, 1.0 Hz, 4,4'-CH), 7.32 (2H, td, *J* 10.2 Hz, 1.1 Hz, 6,6'-CH), 7.14 (2H, t, *J* 9.8 Hz, 5,5'-CH), 6.82 (2H, d, *J* 10.3 Hz, 7,7'-CH), 4.52 (4H, m, 12,12'-CH_2), 3.85-3.70 (4H, m, 9,9'-CH_2), 1.50 (6H, t, *J* 7.1 Hz, 13,13'-CH_3), 0.41 (6H, t, *J* 6.9 Hz, 10,10'-CH_3); δ_C (126 MHz, $CDCl_3$) 168.7 (11,11'-C), 168.5 (2,2'-C), 162.4 (8,8'-C), 137.6 (3a,3a'-C), 132.4 (4,4'-C), 132.0 (6,6'-C), 131.1 (8a,8a'-C), 123.0 (5,5'-C), 112.1 (1,1'-C), 111.8 (7,7'-C), 97.8 (3,3'-C), 64.2 (9,9'-C), 60.1 (12,12'-C), 14.6 (13,13'-C), 13.2 (10,10'-C); ν_{max}(film) 2978, 2925, 1625, 1596, 1572, 1527, 1470, 1438, 1418, 1380, 1356, 1312, 1261, 1214, 1178, 1118, 1095, 1007, 956, 927, 889, 869, 813, 788, 731, 701, 683 cm^{-1}; HRMS (ESI+) *m/z* calc for $[C_{30}H_{30}O_8 + Na]^+$, 541.1838; found, 541.1883.

(R_a)-Diethyl (8,8'-diethoxy-2,2'-bis(((((1*R*,2*S*,5*R*)-2-isopropyl-5-methylcyclohexyl)oxy)carbonyl)oxy)-[1,1'-biazulene]-3,3'-dicarboxylate 22 and (S_a)-Diethyl (8,8'-diethoxy-2,2'-bis(((((1*R*,2*S*,5*R*)-2-isopropyl-5-methylcyclohexyl)oxy)carbonyl)oxy)-[1,1'-biazulene]-3,3'-dicarboxylate 22

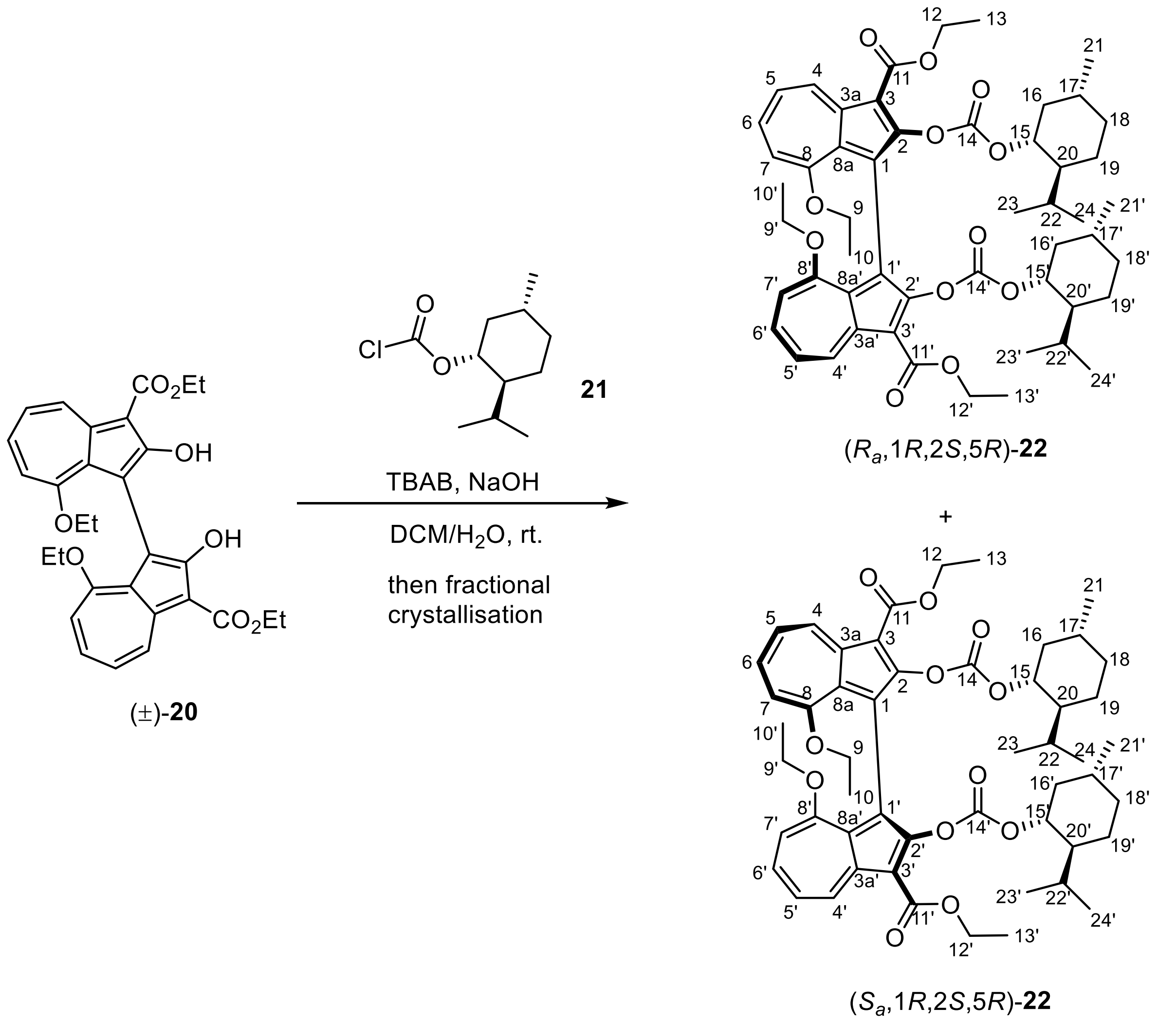

The preparation of these compounds was based on a method by Wan.[10] At r.t., to a mixture of (±)-3,3'-diethyl 2,2'-dihydroxy-8,8'-diethoxy-[1,1'-biazulene]-3,3'-dicarboxylate **20** (1.08 g, 2.07 mmol, 1.00 eq.) and tetra-*n*-butylammonium bromide (267 mg, 0.829 mmol, 0.40 eq.) in DCM (10 mL) was added a solution of NaOH (375 mg, 9.33 mmol, 4.50 eq.) in H_2O (10 mL) and the biphasic mixture was allowed to stir. To this was then slowly added (–)-menthyl chloroformate **21** (1.30 mL, 6.22 mmol, 3.00 eq.), and the mixture was stirred vigorously for 66 h. The mixture was then diluted with DCM (30 mL) and the two phases were separated. The aqueous layer was further extracted with DCM (30 mL), and the combined organic extracts were dried over

anhydrous $MgSO_4$, filtered, and concentrated under reduced pressure to a small volume of DCM. The solution was filtered through a plug of silica gel (25→50% EtOAc/1% NEt_3 in petroleum ether) and concentrated under reduced pressure to give the crude product. Separation of the diastereomers was achieved by recrystallisation (7:1 hexane/THF) to give pure *(*R$_a$*)-diethyl (8,8'-diethoxy-2,2'-bis(((((1*R*,2*S*,5*R*)-2-isopropyl-5-methylcyclohexyl)oxy)carbonyl)oxy)-[1,1'-biazulene]-3,3'-dicarboxylate* **22** (295 mg, 0.333 mmol, 32%) as a fluffy magenta solid, isolated by filtration. The filtrate was then concentrated under reduced pressure, and purified by further recrystallisation (9:1 hexane/THF) to give pure *(*S$_a$*)-diethyl (8,8'-diethoxy-2,2'-bis(((((1*R*,2*S*,5*R*)-2-isopropyl-5-methylcyclohexyl)oxy)carbonyl)oxy)-[1,1'-biazulene]-3,3'-dicarboxylate* **22** (116 mg, 0.131 mmol, 13%) as a fluffy magenta solid, isolated by filtration. The filtrate was then purified by column chromatography (10→17% EtOAc in petroleum ether) and recrystallisation (19:1 hexane/THF) to give a 2nd crop of pure *(*R$_a$*)-diethyl (8,8'-diethoxy-2,2'-bis(((((1*R*,2*S*,5*R*)-2-isopropyl-5-methylcyclohexyl)oxy)carbonyl)oxy)-[1,1'-biazulene]-3,3'-dicarboxylate* **22** (59 mg, 0.0666 mmol, 6.4%) as a fluffy magenta solid.

(*R$_a$*,1*R*,2*S*,5*R*)-**22**: (m.p. 219-221 °C, crystals suitable for X-ray crystallography were grown by storage of a solution of **22** in EtOH at –18 °C); R_f 0.29 (3:1 petroleum ether/EtOAc); $[\alpha]_D^{19}$ -1900 (c 0.005, $CHCl_3$) δ_H (500 MHz, $CDCl_3$) 9.71 (2H, dd, *J* 10.0 Hz, 1.1 Hz, 4,4'-CH), 7.58 (2H, ddd, *J* 10.9 Hz, 9.8 Hz, 1.1 Hz, 6,6'-CH), 7.25 (2H, td, *J* 9.9 Hz, 0.6 Hz, 5,5'-CH), 6.92 (2H, d, *J* 10.9 Hz, 7,7'-CH), 4.37 (2H, q, *J* 7.3 Hz, 12-CH_2 or 12'-CH_2), 4.37 (2H, q, *J* 7.3 Hz, 12-CH_2 or 12'-CH_2), 4.28 (2H, td, *J* 11.0 Hz, 4.4 Hz, 15,15'-CH), 3.82 (app dq, *J* 8.6 Hz, 6.8 Hz, 9,9'-C<u>H</u>H), 3.75 (2H, app dq, *J* 8.4 Hz, 6.9 Hz, 9,9'-CH<u>H</u>), 1.91-1.86 (2H, m, 16,16'-CH_{eq}), 1.57-1.52 (2H, m, 18,18'-C<u>H</u>H), 1.46 (2H, dq, *J* 13.0 Hz, 3.4 Hz, 19,19'-CH_{eq}), 1.38 (6H, t, *J* 7.1 Hz, 13,13'-CH_2), 1.32-1.25 (2H, m, 17,17'-CH), 1.16 (2H, tt, *J* 11.9 Hz, 3.1 Hz, 20,20'-CH), 1.12-1.07 (2H, m, 22,22'-CH), 0.93 (2H, q, *J* 11.5 Hz, 16,16'-CH_{ax}), 0.83 (6H, d, *J* 6.7 Hz, 21,21'-CH_3), 0.85-0.72 (4H, m, 18,18'-CH<u>H</u>, 19,19'-CH_{ax}), 0.46 (6H, d, *J* 6.9 Hz, 24,24'-CH_3), 0.24 (6H, t, *J* 7.1 Hz, 10,10'-CH_3), 0.06 (6H, d, *J* 6.9 Hz, 23,23'-CH_3); δ_C (126 MHz, $CDCl_3$) 165.4 (8,8'-C), 164.8 (11,11'-C), 153.8 (2,2'-C), 152.0 (14,14'-C), 138.2 (4,4'-C), 137.8 (3a,3a'-C), 136.4 (6,6'-C), 128.3 (8a,8a'-C), 122.7 (5,5'-C), 116.0 (1,1'-C), 112.4 (7,7'-C), 105.7 (3,3'-C), 78.3 (15,15'-C), 64.6 (9,9'-C), 59.6 (12,12'-C), 46.9 (20,20'-C), 40.6 (16,16'-C), 34.0 (18,18'-C), 31.3 (17,17'-C), 25.8 (22,22'-C), 23.3 (19,19'-C), 22.0

(21,21'-C), 20.1 (24,24'-C), 15.7 (23,23'-C), 14.5 (13,13'-C), 13.1 (10,10'-C); ν_{max}(film) 2953, 2930, 2869, 1757, 1685, 1595, 1571, 1512, 1450, 1434, 1410, 1384, 1330, 1270, 1227, 1207, 1178, 1094, 1074, 1033, 1000, 980, 958, 925, 891, 827, 781, 720 cm^{-1}; HRMS (ESI+) *m/z* calc for $[C_{52}H_{66}O_{12} + H]^+$, 883.4633; found, 883.4631.

(*S*a,1*R*,2*S*,5*R*)-**22**: (m.p. 213-215 °C, crystals suitable for X-ray crystallography were grown by storage of a solution of **22** in hexane/THF (9:1 v/v) at –18 °C); $[\alpha]_D^{19}$ -1500 (c 0.005, $CHCl_3$); R_f 0.26 (3:1 petroleum ether/EtOAc); δ_H (500 MHz, $CDCl_3$) 9.68 (2H, dd, *J* 10.0 Hz, 1.0 Hz, 4,4'-CH), 7.58 (2H, ddd, *J* 10.9 Hz, 9.8 Hz, 1.1 Hz, 6,6'-CH), 7.25 (2H, td, *J* 9.8 Hz, 0.6 Hz, 5,5'-CH), 6.93 (2H, d, *J* 11.0 Hz, 7,7'-CH), 4.41-4.31 (4H, m, 12,12'-CH_2), 4.25 (2H, td, *J* 10.8 Hz, 4.4 Hz, 15,15'-CH), 3.91-3.79 (4H, m, 9,9'-CH_2), 1.70 (2H, app quint d, *J* 7.0 Hz, 2.4 Hz, 22,22'-CH), 1.56-1.50 (6H, m, 16,16'-CH_{eq}, 18,18'-C<u>H</u>H, 19,19'-CH_{eq}), 1.36 (6H, t, *J* 7.1 Hz, 13,13'-CH_3), 1.27-1.23 (2H, m, 17,17'-CH), 1.22 (2H, tt, *J* 11.6 Hz, 3.0 Hz, 20,20'-CH), 0.90 (2H, qd, *J* 12.8 Hz, 3.4 Hz, 19,19'-CH_{ax}), 0.73 (6H, d, *J* 6.6 Hz, 21,21'-CH_3), 0.71 (2H, m, 18,18'-CH<u>H</u>), 0.69 (6H, d, *J* 7.0, 24,24'-CH_3), 0.63 (2H, app q, *J* 12.1 Hz, 16,16'-CH_{ax}), 0.57 (6H, d, *J* 7.0 Hz, 23,23'-CH_3), 0.36 (6H, t, *J* 6.9 Hz, 10,10'-CH_3); δ_C (126 MHz, $CDCl_3$) 165.1 (8,8'-C), 164.7 (11,11'-C), 154.0 (2,2'-C), 151.4 (14,14'-C), 138.0 (4,4'-C), 137.5 (3a,3a'-C), 136.4 (6,6'-C), 127.3 (8a,8a'-C), 122.6 (5,5'-C), 116.1 (1,1'-C), 112.2 (7,7'-C), 105.8 (3,3'-C), 78.8 (15,15'-C), 64.5 (9,9'-C), 59.6 (12,12'-C), 46.3 (20,20'-C), 39.6 (16,16'-C), 34.0 (18,18'-C), 31.0 (17,17'-C), 25.7 (22,22'-C), 23.2 (19,19'-C), 21.9 (21,21'-C), 20.5 (24,24'-C), 16.0 (23,23'-C), 14.5 (13,13'-C), 13.2 (10,10'-C); ν_{max}(film) 2952, 2952, 2870, 1755, 1678, 1594, 1568, 1511, 1451, 1437, 1409, 1382, 1330, 1233, 1208, 1189, 1179, 1107, 1094, 1074, 1033, 1001, 978, 957, 926, 912, 890, 862, 827, 792, 781, 767, 721, 669 cm^{-1}; HRMS (ESI+) *m/z* calc for $[C_{52}H_{66}O_{12} + H]^+$, 883.4633; found, 883.4643.

(*R*a)-3,3'-Diethyl 2,2'-dihydroxy-8,8'-diethoxy-[1,1'-biazulene]-3,3'-dicarboxylate 20

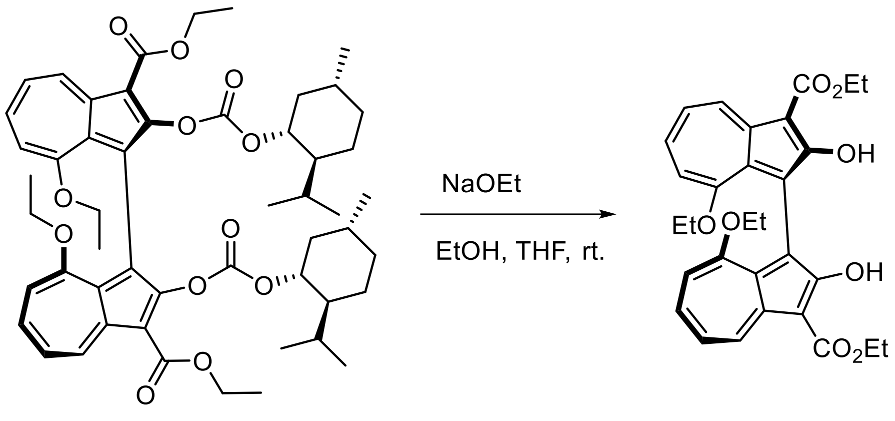

(R_a,1*R*,2*S*,5*R*)-**22** (R_a)-**20**

At r.t., to a stirred solution of (R_a)-diethyl (8,8’-diethoxy-2,2’-bis((((*1R,2S,5R*)-2-isopropyl-5-methylcyclohexyl)oxy)carbonyl)oxy)-[1,1’-biazulene]-3,3’-dicarboxylate **22** (280 mg, 0.317 mmol, 1.00 eq.) in THF (10 mL) was slowly added NaOEt (2.0 M in EtOH, 1.60 mL, 3.20 mmol, 10.0 eq.). After stirring for 2 h, the solution was concentrated under reduced pressure, dissolved in $KOH_{(aq)}$ (1.0 M, 150 mL), and washed with hexane (2 × 40 mL). To the aqueous layer was added $HCl_{(aq)}$ (5.0 M, 60 mL), forming a deep red precipitate, and stored at 2 °C for 1.5 h. The mixture was then filtered, washing with water, to give *(*R_a*)-3,3'-diethyl 2,2'-dihydroxy-8,8'-diethoxy-[1,1'-biazulene]-3,3'-dicarboxylate* **20** (142 mg, 0.274 mmol, 86%) as a deep red solid (crystals suitable for X-ray crystallography were grown by storage of a solution of **20** in EtOH at –18 °C); $[\alpha]_D^{19}$ -2400 (c 0.005, $CHCl_3$).

(S_a)-3,3'-diethyl 2,2'-dihydroxy-8,8'-diethoxy-[1,1'-biazulene]-3,3'-dicarboxylate 20

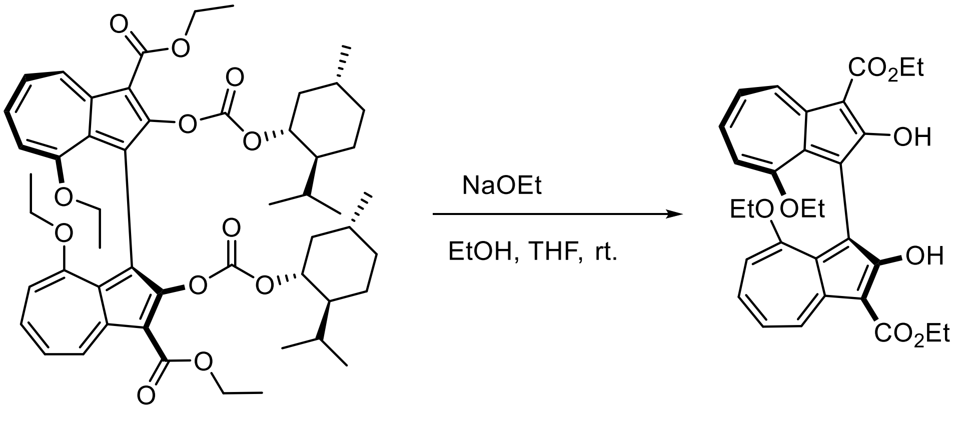

(S_a,1*R*,2*S*,5*R*)-**22** (S_a)-**20**

At 0 °C, to a stirred solution of (S_a)-diethyl (8,8'-diethoxy-2,2'-bis((((*1R,2S,5R*)-2-isopropyl-5-methylcyclohexyl)oxy)carbonyl)oxy)-[1,1'-biazulene]-3,3'-dicarboxylate **22** (131 mg, 0.148 mmol, 1.00 eq.) was slowly added NaOEt (2.0 M in EtOH, 740 µL, 1.48 mmol, 10.0 eq.). On completion of addition, the solution was raised out of ice bath and allowed to warm to r.t., and stirred for 1.5 h. After concentrating under reduced pressure, the solid was dissolved in $KOH_{(aq)}$ (1 M, 30 mL) and water (70 mL), and washed with hexane (2 × 25 mL). On addition of $HCl_{(aq)}$ (5.0 M, 20 mL) to the aqueous layer, a red precipitate formed instantly. After storing at 2 °C for 2.5 h, the mixture was filtered, washing with water, to give (S_a)-*3,3'-diethyl 2,2'-dihydroxy-8,8'-diethoxy-[1,1'-biazulene]-3,3'-dicarboxylate* **20** (66 mg, 0.128 mmol, 86%) as a deep red solid; $[\alpha]_D^{19}$ +2400 (c 0.005, $CHCl_3$).

Ethyl 2,4-dimethoxyazulene-1-carboxylate 24

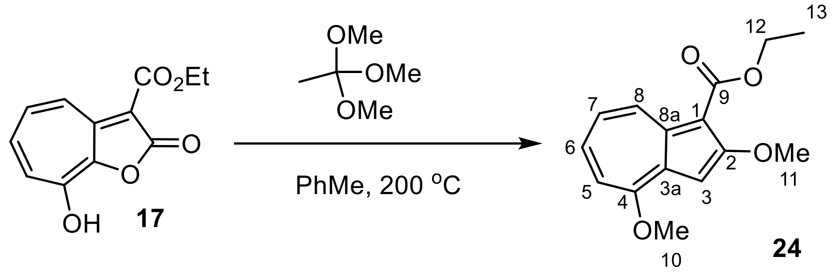

The preparation of this compound was based on a method by Pham.[8] Under atmosphere of air at r.t., to microwave tubes (7 × 10 mL capacity) was added ethyl 8-hydroxy-2-oxo-*2H*-cyclohepta[*b*]furan-3-carboxylate **17** (2.80 g, 12.0 mmol, 1.00 eq.), trimethyl orthoacetate (7.70 mL, 60.5 mmol, 5.05 eq.) and toluene (7.0 mL). The tubes were sealed, and the suspension was stirred, heating under air at 200 °C, for 5 h (CAUTION: the reaction was run behind a blast shield). The resultant deep red solution was loaded onto a silica column, and purified by column chromatography (5→25% EtOAc in petroleum ether) to give *ethyl 2,4-dimethoxyazulene-1-carboxylate* **24** (2.45 g, 9.40 mmol, 79%) as a red, crystalline solid (m.p. 103-105 °C); R_f 0.51 (1:1 petroleum ether/EtOAc); δ_H (500 MHz, $CDCl_3$) 9.39 (1H, d, *J* 10.3 Hz, 8-CH), 7.51 (1H, ddd, *J* 10.9 Hz, 9.9 Hz, 1.0 Hz, 6-CH), 7.29 (1H, t, *J* 10.0 Hz, 7-CH), 7.11 (1H, d, *J* 10.8 Hz, 5-CH), 7.01 (1H, s, 3-CH), 4.45 (2H, q, *J* 6.8 Hz, 12-CH_2), 4.12 (3H, s, 11-CH_3), 4.11 (3H, s, 10-CH_3), 1.45 (3H, t, *J* 7.1 Hz, 13-CH_3); δ_C (126 MHz, $CDCl_3$) 167.6 (2-C), 165.2 (9-C), 160.3 (4-C), 138.9 (8a-C), 135.0 (8-C), 132.8 (6-C), 131.8 (3a-C), 124.0 (7-C), 111.3 (5-C), 96.9 (3-C), 96.8 (1-C), 59.4 (12-C), 58.1 (11-C), 56.4 (10-C), 14.6 (13-C); ν_{max} (film) 2977, 2938, 2837, 1659, 1589, 1575, 1540, 1501, 1455, 1420, 1394, 1344, 1309, 1265, 1198, 1171, 1130, 1088, 1029, 980, 786, 745, 700, 658 cm^{-1}; HRMS (ESI+) *m/z calc* for $[C_{15}H_{16}O_4 + Na]^+$, 283.0941; found, 283.0953.

Ethyl 2-hydroxy-4-methoxyazulene-1-carboxylate 25

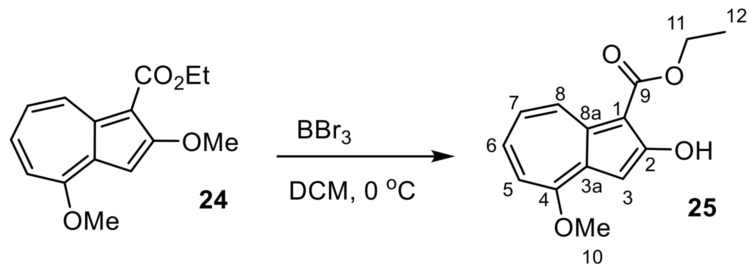

At 0 °C, to a stirred solution of ethyl 2,4-dimethoxyazulene-1-carboxylate **24** (2.45 g, 9.41 mmol, 1.00 eq.) in DCM (50 mL) was added slowly BBr_3 (1.0 M in hexanes, 9.50 mL, 1.01 eq.). After stirring for 40 min, to the reaction mixture was added methanol (5.0 mL) to quench. The solution was diluted with DCM (100 mL) and washed with water (3 × 50 mL). The organic layer was dried over anhydrous $MgSO_4$, filtered and the filtrate was concentrated under reduced pressure to give *2-hydroxy-4-methoxyazulene-1-carboxylate* **25** (2.28 g, 9.23 mmol, 98%) as an orange/brown crystalline solid (m.p. 98-100 °C); R_f 0.37 (4:1 petroleum ether/EtOAc); δ_H (300 MHz, $CDCl_3$) 10.82 (1H, s, 2-OH), 8.84 (1H, d, *J* 9.5 Hz, 8-CH), 7.38 (1H, td, *J* 10.2 Hz, 0.9 Hz, 6-CH), 7.17 (1H, t, *J* 10.0 Hz, 7-CH), 6.94 (1H, s, 3-CH), 6.93 (1H, d, *J* 10.6 Hz, 5-CH), 4.50 (2H, q, *J* 7.2 Hz, 10-CH_2), 4.01 (3H, s, 12-CH_3), 1.51 (3H, t, *J* 7.2 Hz, 11-CH_3); δ_C (75 MHz, $CDCl_3$) 169.4 (2-C), 168.6 (9-C), 160.4 (4-C), 136.6 (8a-C), 133.6 (3a-C), 132.7 (8-C), 132.0 (6-C), 123.5 (7-C), 110.5 (5-C), 101.3 (3-C), 98.5 (1-C), 60.0 (11-C), 55.9 (10-C), 14.3 (12-C); ν_{max}(film) 2981, 2936, 2908, 2837, 1628, 1596, 1535, 1478, 1450, 1436, 1396, 1381, 1351, 1316, 1266, 1171, 1128, 1091, 1069, 1021, 958, 924, 871, 821, 806, 785, 763, 739, 714, 670 cm^{-1}; HRMS (ESI+) *m/z* calc for $[C_{14}H_{14}O_4 + Na]^+$, 269.0790; found, 269.0785.

(±)-3,3'-Diethyl 2,2'-dihydroxy-8,8'-dimethoxy-[1,1'-biazulene]-3,3'-dicarboxylate 26

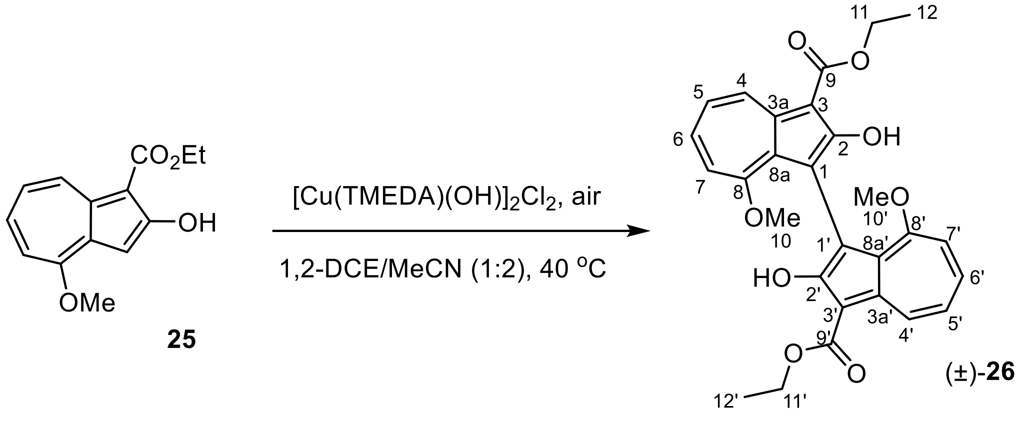

Under atmosphere of air, at r.t., to a stirred solution of ethyl 2-hydroxy-4-methoxyazulene-1-carboxylate **25** (2.20 g, 8.94 mmol, 1.00 eq.) in 1,2-dichloroethane (10 mL) and acetonitrile (20 mL) was added di-μ-hydroxo-bis[(*N*,*N*,*N'*,*N'*-tetramethylethylenediamine)copper(II)] chloride (208 mg, 0.894 mmol (by mol. weight of monomer), 0.10 eq.). The mixture was stirred at 40 °C for 89 h in a sealed vessel. After allowing to cool to r.t., the suspension was stored at –18 °C for 2 h, and the mixture was filtered, washing with cold $CHCl_3$, to give *(±)-3,3'-diethyl 2,2'-dihydroxy-8,8'-dimethoxy-[1,1'-biazulene]-3,3'-dicarboxylate* **26** (1.08 g, 2.20 mmol, 49%) as a red solid. After storing the filtrate for an additional 3 days at -18 °C, a 2nd crop of product (124 mg, 0.252 mmol, 5.6%) was collected by filtration as a red solid (m.p. >300 °C (dec. ca. 233 °C)); δ_H (250 MHz, $CDCl_3$) 10.84 (2H, br s, 2,2'-OH), 9.03 (2H, d, *J* 9.5 Hz, 4,4'-CH), 7.41 (2H, t, *J* 10.2 Hz, 6,6'-CH), 7.21 (2H, t, *J* 10.0 Hz, 5,5'-CH), 6.93 (2H, d, *J* 10.5 Hz, 7,7'-CH), 4.60-4.50 (4H, m, 11,11'-CH_2), 3.58 (6H, s, 10,10'-CH_3), 1.52 (6H, t, *J* 7.0 Hz, 12,12'-CH_3); δ_C (75 MHz, $CDCl_3$) 169.1 (9,9'-C or 2,2'-C), 169.0 (9,9'-C or 2,2'-C), 163.0 (8,8'-C), 137.7 (3a,3a'-C), 132.3 (4,4'-C or 6,6'-C), 132.0 (4,4'-C or 6,6'-C), 130.4 (8a,8a'-C), 123.5 (5,5'-C), 111.9 (1,1'-C), 111.7 (7,7'-C), 98.0 (3,3'-C), 60.3 (11,11'-C), 56.5 (10,10'-C), 14.7 (12,12'-C); ν_{max}(film) 2924, 2852, 1740, 1686, 1628, 1595, 1572, 1449, 1422, 1396, 1380, 1357, 1313, 1259, 1215, 1205, 1183, 1170, 1130, 1098, 1082, 1022, 957, 870, 838, 810, 789, 746, 725, 698 cm^{-1}; HRMS (ESI+) *m/z* calc for $[C_{28}H_{26}O_8 + Na]^+$, 513.1525; found, 513.1551.

(±)-3,3'-Diethyl 8,8'-dimethoxy-2,2'-bis(trifluoromethanesulfonyloxy)-[1,1'-biazulene]-3,3'-dicarboxylate 27

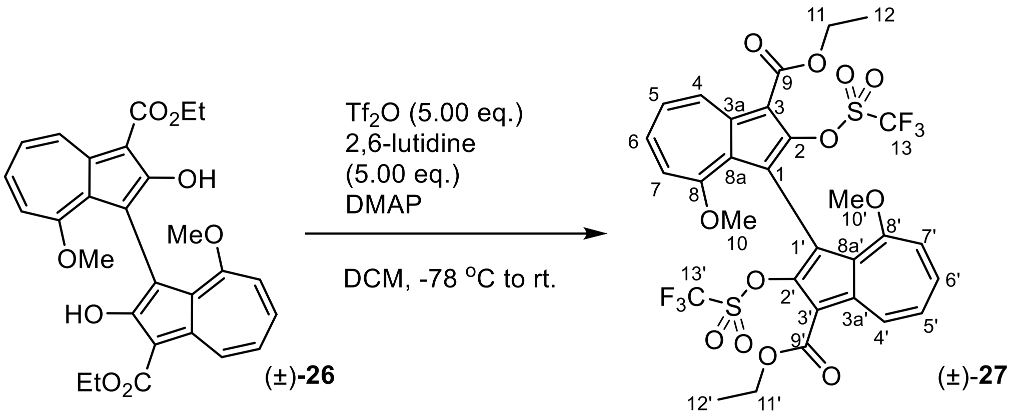

At –78 °C, to a stirred suspension of 3,3'-diethyl 2,2'-dihydroxy-8,8'-dimethoxy-[1,1'-biazulene]-3,3'-dicarboxylate **26** (200 mg, 0.408 mmol, 1.00 eq.), 4-(dimethylamino)pyridine (10 mg, 0.0816 mmol, 0.20 eq.) and 2,6-lutidine (240 µL, 2.07 mmol, 5.06 eq.) in DCM (10.0 mL) was added trifluoromethanesulfonic anhydride (170 µL, 1.01 mmol, 2.46 eq.). The mixture temperature was maintained at –78 °C for 5 h. To it was then added trifluoromethanesulfonic anhydride (170 µL, 1.01 mmol, 2.46 eq.), the mixture was allowed to warm to r.t. in dry ice/acetone bath. After stirring for an additional 17 h, water (5.0 mL) was added slowly, and allowed to stir for 5 min. The mixture was diluted with DCM (30 mL), and the two phases were separated. The organic phase was washed further with water (3 × 15 mL), dried over anhydrous $MgSO_4$, and filtered. The filtrate was concentrated under reduced pressure to give the crude product, which was purified by column chromatography (10→50% EtOAc in petroleum ether) to give *(±)-3,3'-diethyl 8,8'-dimethoxy-2,2'-bis(trifluoromethanesulfonyloxy)-[1,1'-biazulene]-3,3'-dicarboxylate* **27** (145 mg, 0.193 mmol, 47%) as a magenta solid (m.p. 183-185 °C); R_f 0.23 (1:1 petroleum ether/EtOAc); δ_H (500 MHz, $CDCl_3$) 9.81 (2H, dd, *J* 10.2 Hz, 1.1 Hz, 4,4'-CH), 7.81 (2H, ddd, *J* 10.9 Hz, 9.7 Hz, 1.1 Hz, 6,6'-CH), 7.43 (2H, t, *J* 9.8 Hz, 5,5'-CH), 7.14 (2H, d, *J* 11.1 Hz, 7,7'-CH), 4.51 (4H, q, *J* 7.0 Hz, 11,11'-CH_2), 3.61 (6H, s, 10,10'-CH_3), 1.47 (6H, t, *J* 7.1 Hz, 12,12'-CH_3); δ_C (126 MHz, $CDCl_3$) 167.2 (8,8'-C), 163.8 (9,9'-C), 149.4 (2,2'-C), 140.1 (4,4'-C), 138.7 (6,6'-C), 136.6 (3a,3a'-C), 126.4 (8a,8a'-C), 124.2 (5,5'-C), 117.9 (q, $^1J_{CF}$ 321.0 Hz, 13,13'-C) 113.0 (7,7'-C), 112.6 (1,1'-C), 106.8 (3,3'-C), 60.5 (11,11'-C), 56.6 (10,10'-C), 14.3 (12,12'-C); ν_{max}(film) 2924, 2851, 1690, 1598, 1570, 1516, 1458, 1412, 1391, 1316, 1268, 1191, 1135, 1086, 1048, 1021, 954, 923, 875, 846, 832, 805, 790, 759, 726, 690 cm^{-1} ; HRMS (ESI+) *m/z* calc for $[C_{30}H_{24}F_6O_{12}S_2 + Na]^+$, 777.0511; found, 777.0513.

(±)-8,8'-Dimethoxy-2'-(trifluoromethanesulfonyloxy)-[1,1'-biazulene]-2-yl trifluoromethanesulfonate 28 and (±)-2'-ethoxy-8,8'-dimethoxy-[1,1'-biazulen]-2-yl trifluoromethanesulfonate S2

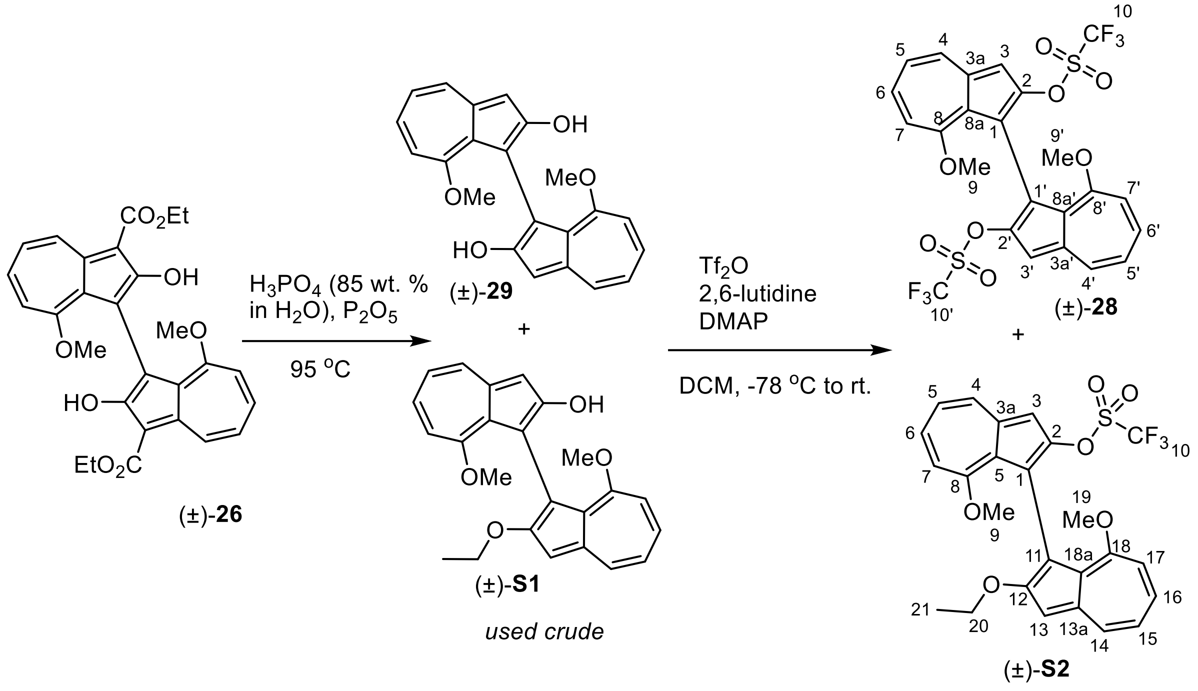

Under atmosphere of air, at 0 °C, to phosphorus pentoxide (800 mg) was added $H_3PO_{4(aq)}$ (85 wt. %, 1.20 mL). The mixture was then stirred at 95 °C, and to it was added (±)-3,3'-diethyl 2,2'-dihydroxy-8,8'-dimethoxy-[1,1'-biazulene]-3,3'-dicarboxylate **26** (200 mg, 0.408 mmol, 1.00 eq.), stirred for 2 h, and then allowed to cool to r.t. To the resultant slurry was slowly added water (20 mL), and extracted with DCM (2 × 25 mL). The organic extracts were combined and washed with saturated aqueous $NaHCO_3$ (20 mL) and water (2 × 20 mL). The organic layer was then dried over anhydrous $MgSO_4$, and filtered. The filtrate was concentrated under reduced pressure to give the crude intermediate mixture as a maroon oil. Under atmosphere of N_2, at –78 °C, to a stirred solution of the crude diol, 4-(dimethylamino)pyridine (10 mg, 0.0816 mmol, 0.20 eq.) and 2,6-lutidine (250 µL, 2.15 mmol, 5.27 eq.) in DCM (20 mL) was added dropwise trifluoromethanesulfonic anhydride (170 µL, 1.01 mmol, 2.47 eq.), and the mixture was allowed to stir at –78 °C for 45 min. To this was then added trifluoromethanesulfonic anhydride (170 µL, 1.01 mmol, 2.47 eq.), and stirred at –78 °C for another 45 min. The mixture was allowed to warm to r.t., and to it was added water (20 mL), allowing to stir for 10 min, and then the phases were separated. The aqueous layer was extracted with DCM (60 mL), and the combined organic extracts

were washed with water (2 × 20 mL), dried over anhydrous $MgSO_4$, and filtered. The filtrate was concentrated under reduced pressure to give the crude product mixture, which was purified by column chromatography (10→50% EtOAc in petroleum ether) to give *(±)-8,8'-dimethoxy-2'-(trifluoromethanesulfonyloxy)-[1,1'-biazulene]-2-yl trifluoromethanesulfonate* **28** (110 mg, 0.181 mmol, 44%) as a purple crystalline solid (m.p. 174-176 °C); R_f 0.19 (4:1 petroleum ether/EtOAc); δ_H (500 MHz, $CDCl_3$) 8.28 (2H, d, *J* 9.2 Hz, 4,4'-CH), 7.59 (2H, td, *J* 11.0 Hz, 9.8 Hz, 1.2 Hz, 6,6'-CH), 7.21 (2H, s, 3,3'-CH), 7.08 (2H, t, *J* 9.5 Hz, 5,5'-CH), 6.91 (2H, d, *J* 11.0 Hz, 7,7'-CH), 3.69 (6H, s, 9-CH_3); δ_C (126 MHz, $CDCl_3$) 165.6 (8,8'-C), 150.0 (2,2'-C), 139.1 (4,4'-C), 136.6 (6,6'-C), 136.3 (3a,3a'-C), 122.1 (8a,8a'-C), 120.3 (5,5'-C), 118.4 (d, $^1J_{CF}$ 320.4 Hz, 10,10'-C), 112.4 (1,1'-C), 109.6 (7,7'-C), 105.2 (3,3'-C), 56.3 (9,9'-C); ν_{max}(film) 3015, 2939, 2844, 1597, 1568, 1523, 1482, 1457, 1441, 1411, 1382, 1265 1241, 1230, 1196, 1181, 1162, 1078, 1004, 958, 916, 875, 822, 788, 781, 764, 745, 725, 690, 679 cm^{-1}; HRMS (ESI+) *m/z* calc for $[C_{24}H_{16}F_6O_4S_2 + H]^+$, 611.0264; found, 611.0277. Also isolated *(±)-2'-ethoxy-8,8'-dimethoxy-[1,1'-biazulen]-2-yl trifluoromethanesulfonate* **S2** (10 mg, 0.0197 mmol, 4.8%) as a pale purple solid (m.p. 127-130 °C); R_f 0.23 (4:1 petroleum ether/EtOAc); δ_H (500 MHz, $CDCl_3$) 8.23 (1H, d, *J* 9.6 Hz, 4-CH), 8.01 (1H, d, *J* 9.1 Hz, 14-CH), 7.51 (1H, ddd, *J* 11.0 Hz, 9.8 Hz, 1.2 Hz, 6-CH), 7.28 (1H, ddd, *J* 10.8 Hz, 10.0 Hz, 1.0 Hz, 16-CH), 7.19 (1H, s, 3-CH), 7.00 (1H, t, *J* 9.6 Hz, 5-CH), 6.96 (1H, t, *J* 9.6 Hz, 15-CH), 6.82 (1H, d, *J* 11.0 Hz, 7-CH), 6.77 (1H, d, *J* 10.7 Hz, 17-CH), 6.70 (1H, s, 13-CH), 4.23 (2H, q, *J* 7.0 Hz, 20-CH_2), 3.60 (3H, s, 9-CH_3), 3.53 (3H, s, 19-CH_3), 1.32 (3H, t, *J* 7.0 Hz, 21-CH_3); δ_C (126 MHz, $CDCl_3$) 166.2 (8-C), 164.7 (12-C), 161.3 (18-C), 150.2 (2-C), 139.9 (13a-C), 138.7 (4-C), 136.2 (6-C), 136.1 (3a-C), 131.8 (14-C), 130.4 (16-C), 124.3 (18a-C), 122.4 (8a-C), 119.7 (15-C), 119.5 (5-C), 118.4 (q, $^1J_{CF}$ 321 Hz, 10-C), 115.9 (1-C), 111.6 (11-C), 109.3 (7-C), 108.6 (17-C), 105.5 (3-C), 97.3 (13-C), 65.6 (20-C), 56.23 (9-C or 19-C), 56.19 (9-C or 19-C), 14.8 (21-C); ν_{max}(film) 2985, 2935, 2849, 1594, 1567, 1518, 1494, 1456, 1408, 1387, 1359, 1261, 1243, 1202, 1187, 1163, 1137, 1116, 1100, 1041, 1006, 974, 942, 887, 843, 827, 784, 767, 728, 707, 684, 662 cm^{-1}; HRMS (ESI+) *m/z* calc for $[C_{25}H_{21}F_3O_6S + Na]^+$, 529.0903; found, 529.0912.

In a separate experiment on a larger scale, the desired product **28** was isolated in an increased yield (168 mg, 0.274 mmol, 52%), with only a trace of side product **S2**.

(±)-Diethyl (2'-(diethoxyphosphoryl)-8,8'-dimethoxy-[1,1'-biazulene]-2-yl)phosphonate 30

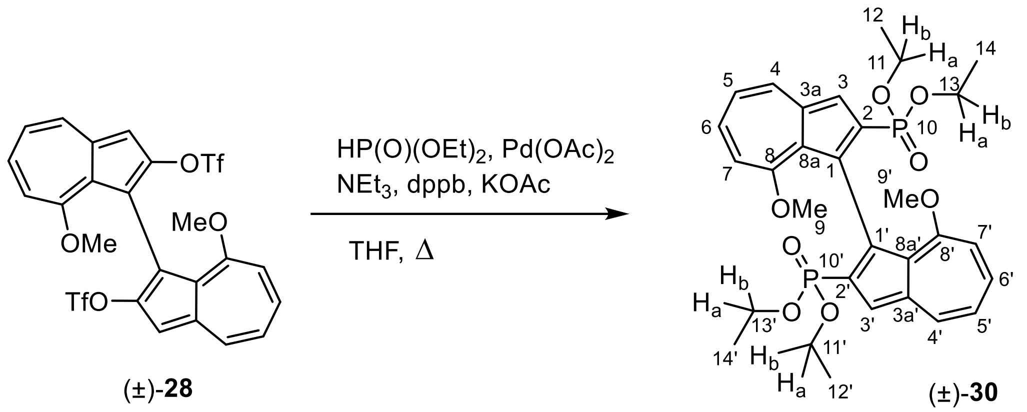

The preparation of this compound was based on a method by Stawinski.[11] A mixture of palladium diacetate (5.5 mg, 0.0246 mmol, 0.10 eq.), 1,4-bis(diphenylphosphino)butane (21 mg, 0.0492 mmol, 0.20 eq.), potassium acetate (24 mg, 0.246 mmol, 1.00 eq.) was dissolved in THF (0.5 mL) and stirred at 60 °C for 30 min. To the mixture was then added triethylamine (100 µL, 0.738 mmol, 3.00 eq.), diethyl phosphite (100 µL, 0.738 mmol, 3.00 eq.) and a solution of (±)-8,8'-dimethoxy-2'-(trifluoromethanesulfonyloxy)-[1,1'-biazulene]-2-yl trifluoromethanesulfonate **28** (150 mg, 0.246 mmol, 1.00 eq.) in THF (1.5 mL), and heated at 68 °C for 45 h. After allowing to cool to r.t., the mixture was concentrated under reduced pressure and purified by column chromatography (0→10% MeOH in DCM) to give the crude product. After dissolving in ethyl acetate (20 mL), washing with water (5 × 10 mL) and with saturated brine, the solution was concentrated under reduced pressure to give *(±)-diethyl [2'-(diethoxyphosphoryl)-8,8'-dimethoxy-[1,1'-biazulene]-2-yl]phosphonate* **30** (56 mg, 0.0958 mmol, 39%) as a deep blue solid (m.p. 156-158 °C); R_f 0.21 (19:1 DCM/MeOH); δ_H (500 MHz, $CDCl_3$) 8.25 (2H, d, *J* 9.3 Hz, 4,4'-CH), 7.69 (2H, d, $^3J_{PH}$ 6.9 Hz, 3,3'-CH), 7.48 (2H, t, *J* 10.2 Hz, 6,6'-CH), 6.84 (2H, t, *J* 9.6 Hz, 5,5'-CH), 6.66 (2H, d, *J* 10.8 Hz, 7,7'-CH), 3.94-3.87 (2H, m, 11a,11a'-CH_2), 3.85-3.77 (2H, m, 11b,11b'-CH_2), 3.60-3.54 (2H, m, 13a,13a'-CH_2), 3.52 (6H, s, 9,9'-CH_3), 3.42-3.34 (2H, m, 13b,13b'-CH_2), 1.10 (6H, t, *J* 7.1 Hz, 12,12'-CH_3), 0.81 (6H, t, *J* 7.1 Hz, 14,14'-CH_3); δ_C 167.5 (8,8'-C), 140.2 (4,4'-C), 138.5 (6,6'-C), 138.3 (d, $^3J_{CP}$ 20.0 Hz, 3a,3a'-C), 132.3 (d, $^1J_{CP}$ 199.9 Hz, 2,2'-C), 130.3 (d, $^2J_{CP}$ 14.2 Hz, 1,1'-C) 124.6 (d, $^3J_{CP}$ 17.4 Hz, 8a,8a'-C), 123.9 (d, $^2J_{CP}$ 13.4 Hz, 3,3'-C), 117.9 (d, $^5J_{CP}$ 1.9 Hz, 5,5'-C), 108.5 (d, $^5J_{CP}$ 1.2 Hz, 7,7'-C), 61.1 (d, $^2J_{CP}$ 5.9 Hz, 11,11'-C), 60.9 (d, $^2J_{CP}$ 6.3 Hz, 13,13'-C), 56.3 (9,9'-C), 16.2 (d, $^3J_{CP}$ 6.7 Hz, 12,12'-C), 16.1 (d, $^3J_{CP}$ 6.3 Hz, 14,14'-C); δ_P (202 MHz,

$CDCl_3$) 17.6 (10,10'-P); ν_{max}(film) 2975, 2925, 2850, 1597, 1561, 1528, 1496, 1477, 1450, 1436, 1408, 1386, 1364, 1341, 1292, 1264, 1242, 1229, 1217, 1193, 1178, 1159, 1122, 1095, 1047, 1025, 941, 909, 841, 824, 789, 726, 695, 666 cm^{-1}; HRMS (ESI+) *m/z* calc for $[C_{30}H_{36}O_8P_2 + H]^+$, 587.1964; found 587.1929.

Calculation of barrier to racemisation of 1,1'-BazOL 20

To calculate the relevant kinetic parameters, a CD spectrum of a 0.01 mM solution of (S_a)-1,1'-BAzOL (+)-**20** in 1,1,2,2-tetrachloroethane was recorded every 5 minutes for 845 minutes at three temperatures: 333.15 K, 343.15 K, 353.15 K (60 °C, 70 °C, 80 °C) (Figures S1-S3). As expected, the rate of racemisation increased with a higher temperature.

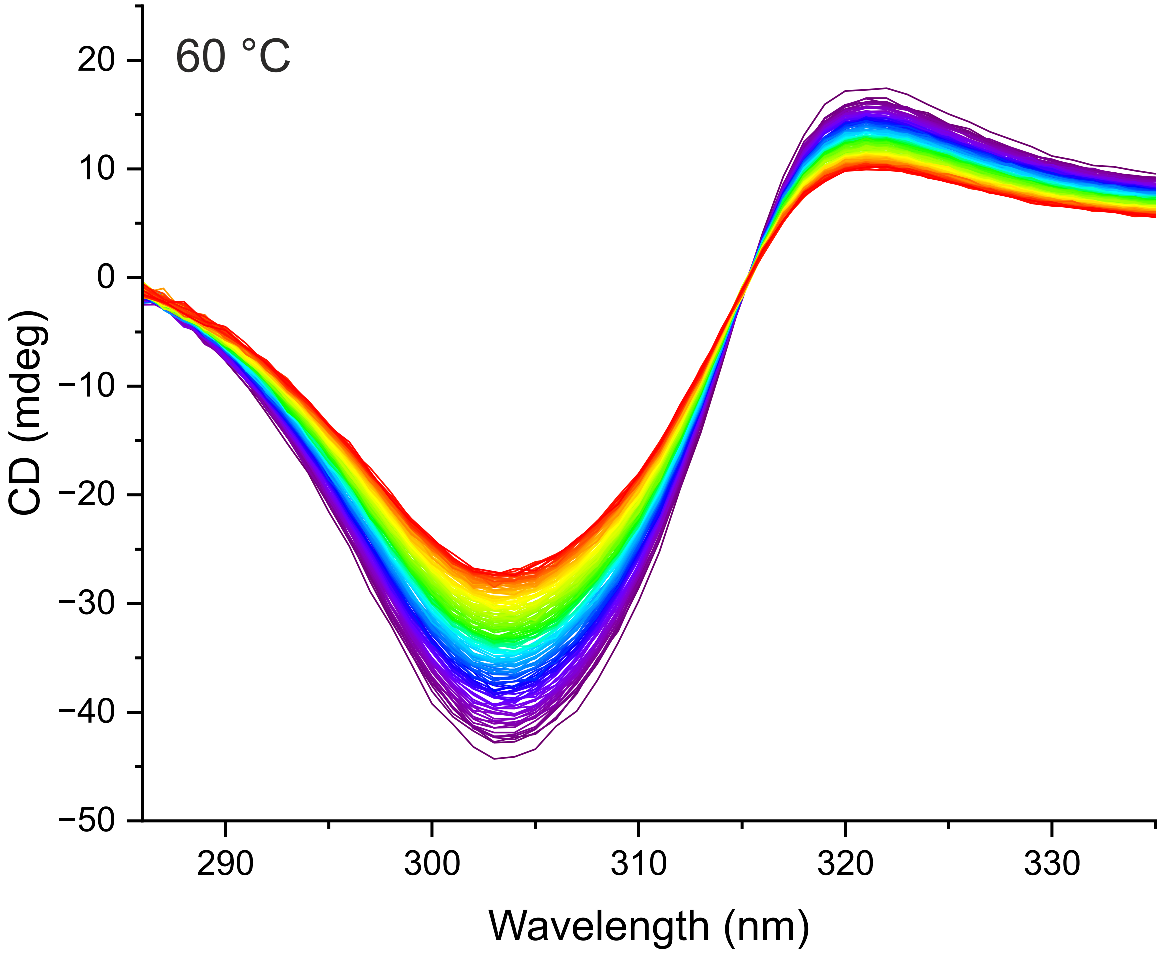

Figure S1: CD spectra of (S_a)-1,1'-BAzOL (+)-**20** in 1,1,2,2,-tetrachloroethane at 0.01 mM concentration at 333.15 K.

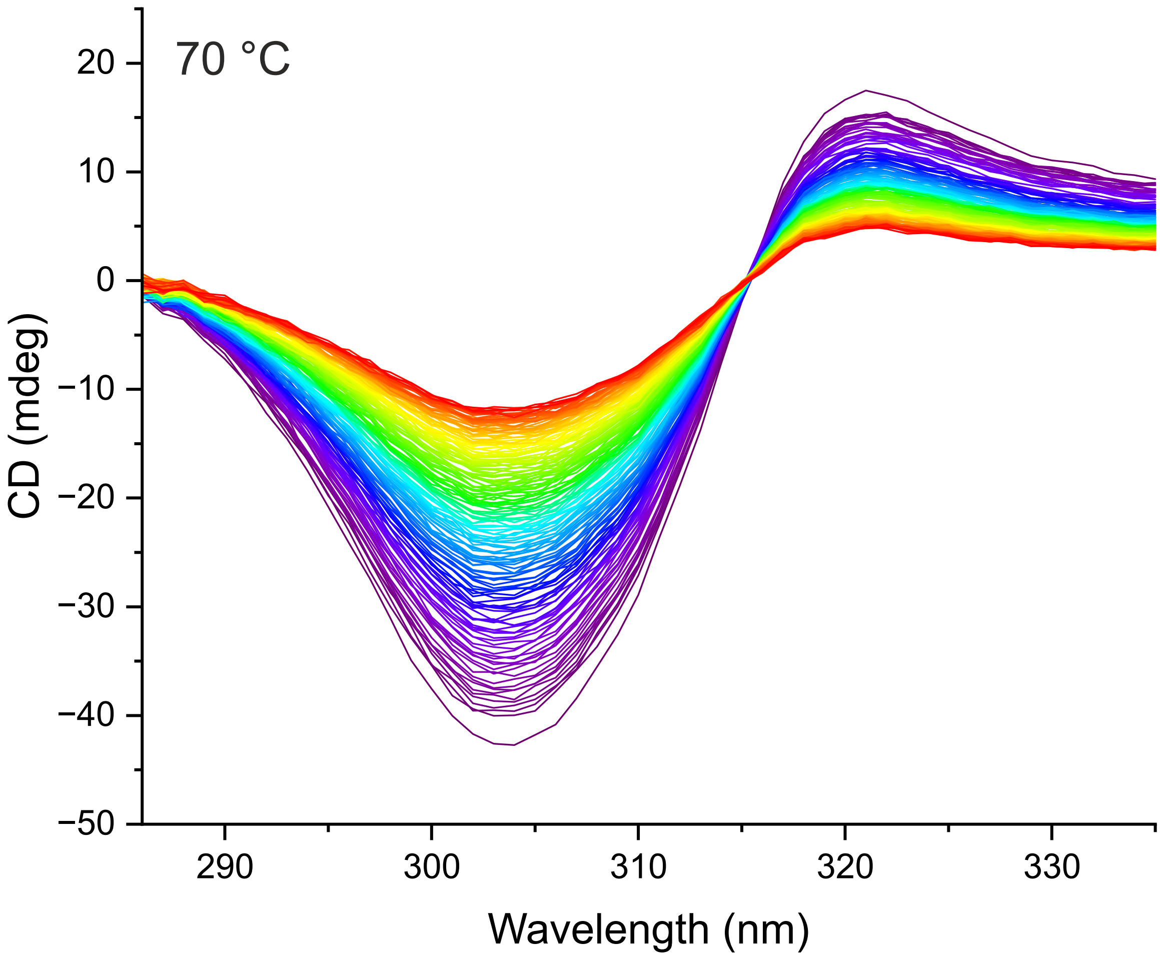

Figure S2: CD spectra of (S_a)-1,1'-BAzOL (+)-**20** in 1,1,2,2,-tetrachloroethane at 0.01 mM concentration at 343.15 K.

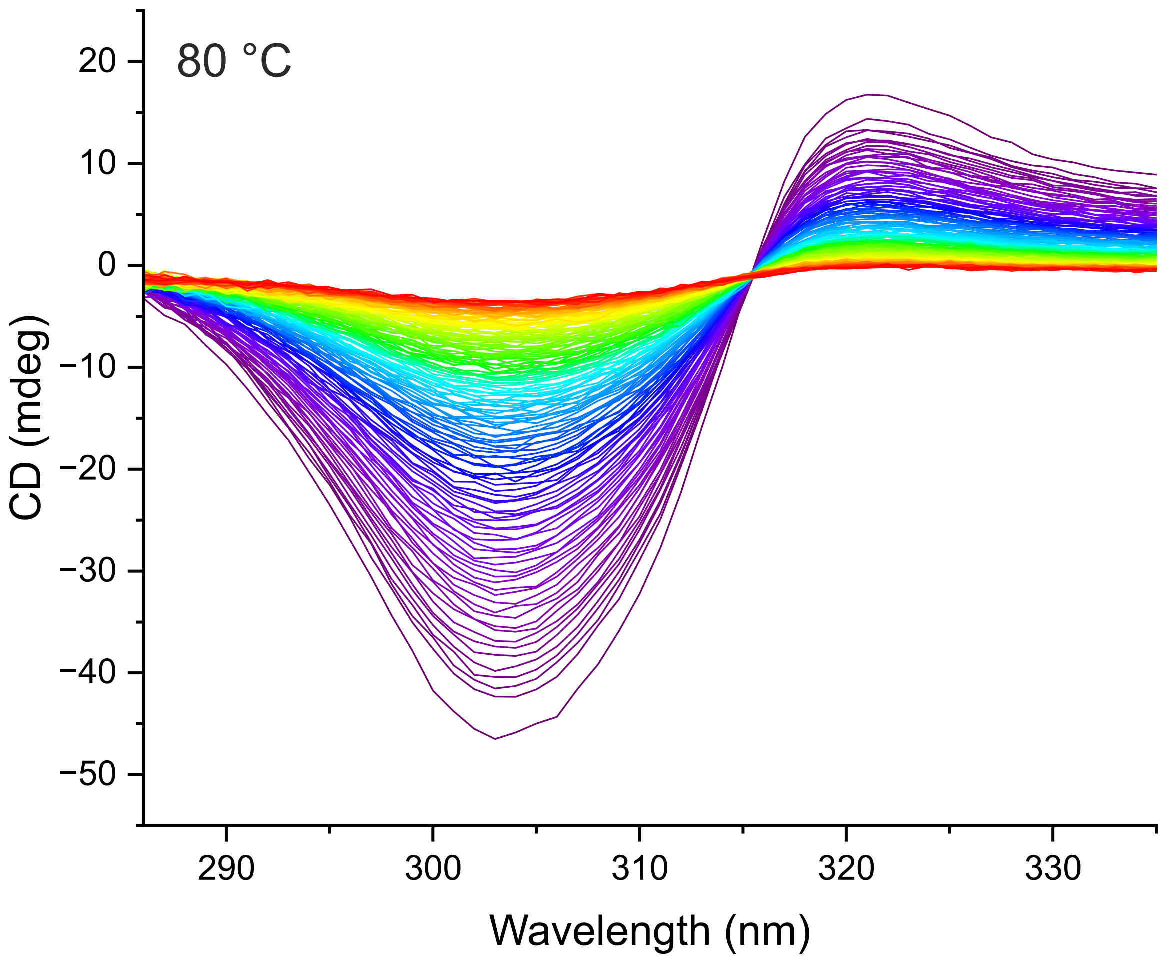

Figure S3: CD spectra of (S_a)-1,1'-BAzOL (+)-**20** in 1,1,2,2,-tetrachloroethane at 0.01 mM concentration at 353.15 K.

To calculate the barrier to racemisation from these data, the rate constant (k) of the racemisation had to be calculated for each temperature. Since the absorption of the circular polarised light was strong at 304 nm, a first order rate equation was adopted for the data at this wavelength (Figure S4).

$$\ln \frac{C}{C_0} = -kt$$

$$\ln C = -kt - \ln C_0$$

where: $ln\,C = \ln(-\mathrm{CD})_t$; $t = \mathrm{time}$; $ln\ A_0 = \ln(-\mathrm{CD})_0$

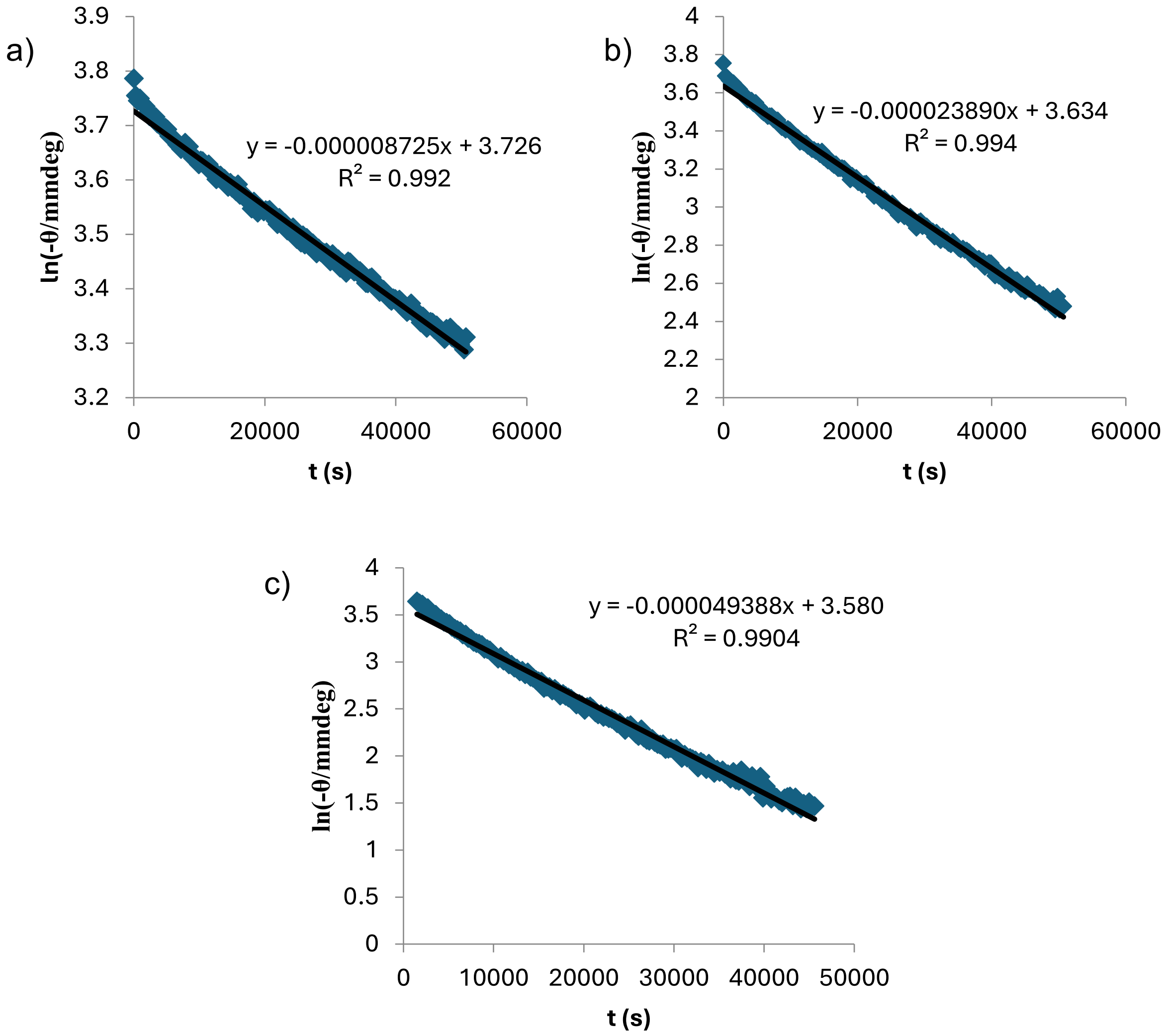

Figure S4: Linear regression analysis of the kinetic measurements at 333.15 K (a); 343.15 K (b); 353.15 K (c); using data points at λ = 304 nm.

Table S1: The rate constants calculated from the linear regression analysis of the kinetic measurements at different temperatures.

T (K)	k (s^{-1})	1/T (K^{-1})
333.15	8.725 x 10^{-6}	0.003002
343.15	23.890 x 10^{-6}	0.002914
353.15	49.388 x 10^{-6}	0.002832

From the three values for the rate constant k, the activation energy (E_a) could now be calculated using the linear form of the Arrhenius equation (Figure S5).

$$\ln k = -\frac{E_a}{R}\frac{1}{T} + \ln A$$

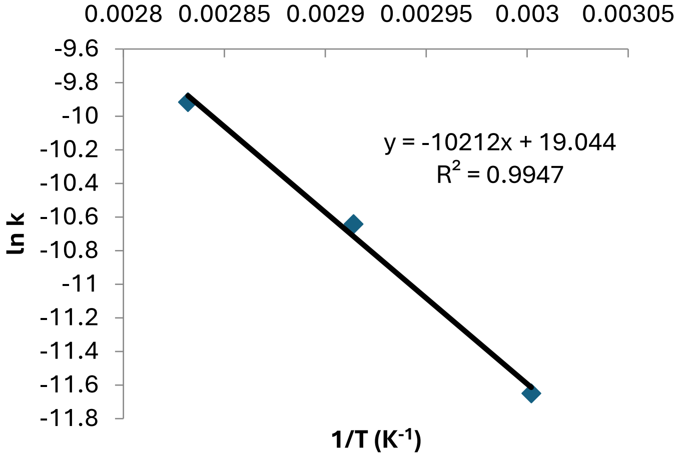

Figure S5: Linear regression analysis of the rate constants k, related to the different temperatures.

The activation energy E_a is therefore 84.902 kJ mol^{-1}, with the pre-exponential factor equating to $e^{19.044}$ s^{-1}. These values can be used to calculate the half-life of racemisation in solution of (S_a)-1,1'-BAzOL (+)-**20** at 293.15 K (20 °C) through application to the Arrhenius equation. The rate constant at this temperature is 1.38 × 10^{-7} s^{-1}, so therefore the half-life is 1389 h, or 57.9 days. To quantify the barrier to racemisation, or the Gibbs free energy of activation ΔG‡, the linear form of the Eyring equation can be employed, plotting the ln(k/T) against 1/T (Figure S6).

$$\ln\frac{k}{T} = -\left(\frac{\Delta H^{\ddagger}}{R}\right)\frac{1}{T} + \frac{\Delta S^{\ddagger}}{R} + \ln\frac{k_B}{h}$$

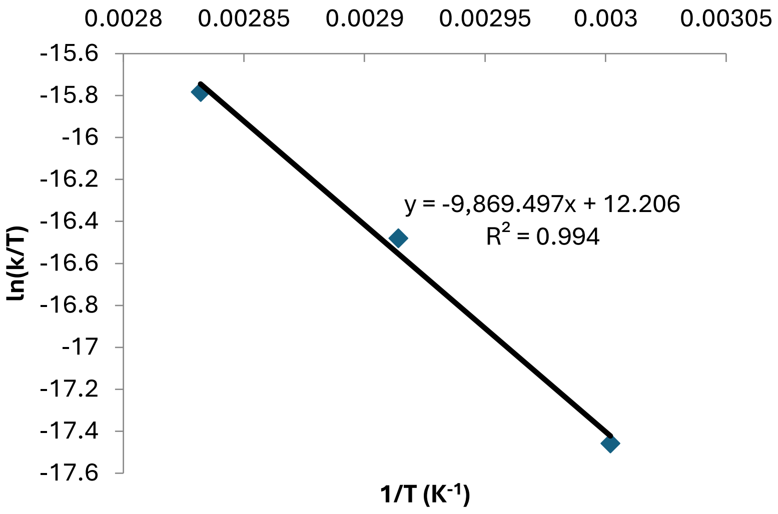

Figure S6: Linear regression analysis of the Eyring equation for the calculation of thermodynamic quantities of activation.

From the linear Eyring plot, the enthalpy and entropy of activation can be calculated as $\Delta H^{\ddagger}$ = 82.055 kJ mol^{-1} and $\Delta S^{\ddagger}$ = - 96.060 J mol^{-1} K^{-1}, respectively. The equation for $\Delta G^{\ddagger}$ is expressed as:

$$\Delta G^{\ddagger} = \Delta H^{\ddagger} - T\Delta S^{\ddagger}$$

Therefore, the Gibbs free energy of activation, or barrier to racemisation, at 293.15 K (20 °C) is:

$$\Delta G^{\ddagger}_{293.15\,K} = 110.25\ kJ\ mol^{-1}$$

The thermodynamic and kinetic values are summarised in Table S2.

Table S2: Summary of the thermodynamic and kinetic quantities for (S_a)-1,1'-BAzOL (+)-**20**.

$k_{333.15\,K}$ /s^{-1}	$k_{343.15\,K}$ /s^{-1}	$k_{353.15\,K}$ /s^{-1}	E_a /kJ mol^{-1}	A /s^{-1}	$\Delta H^{\ddagger}$ /kJ mol^{-1}	$\Delta S^{\ddagger}$ /J mol^{-1} K^{-1}	$\Delta G^{\ddagger}_{293.15\,K}$ /kJ mol^{-1}
8.725 x 10^{-6}	23.890 x 10^{-6}	49.388 x 10^{-6}	84.902	1.865 × 10^{8}	82.055	-96.060	110.215

By using the equation for the Gibbs free energy of activation, the relationship between ΔG‡ and temperature can be plotted (Figure S7).

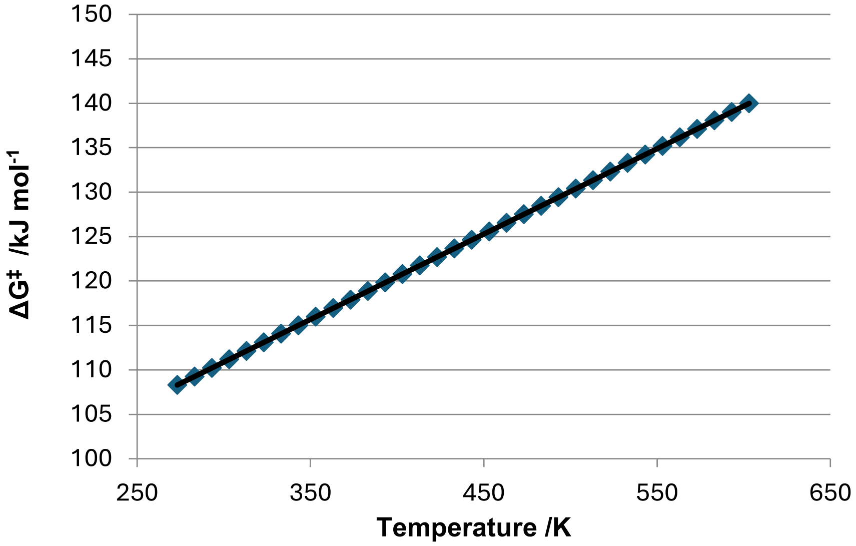

Figure S7: The Gibbs free energy of activation of (S_a)-1,1'-BAzOL (+)-**20** as a function of temperature.

NMR Spectra

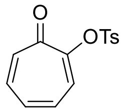

16

^{1}H-NMR, 250 MHz, $CDCl_3$

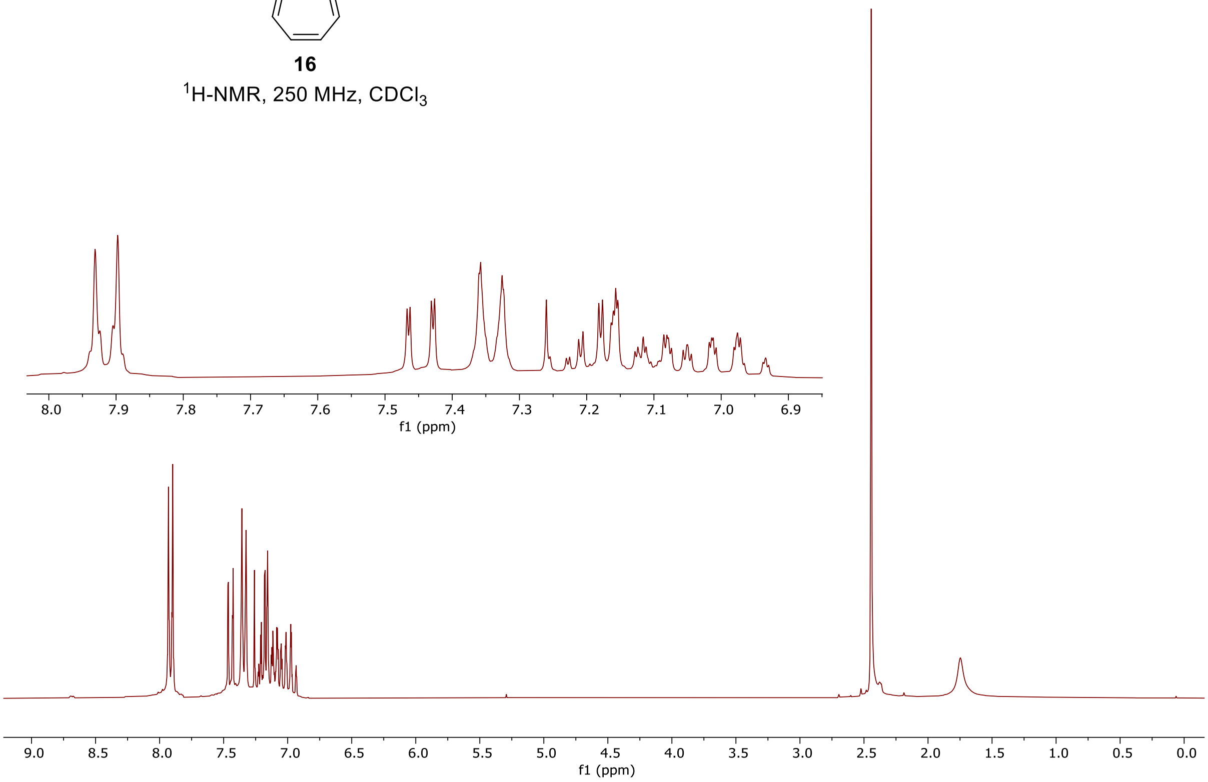

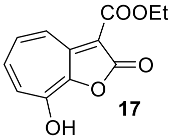

17

^{1}H-NMR, 250 MHz, DMSO-d_6

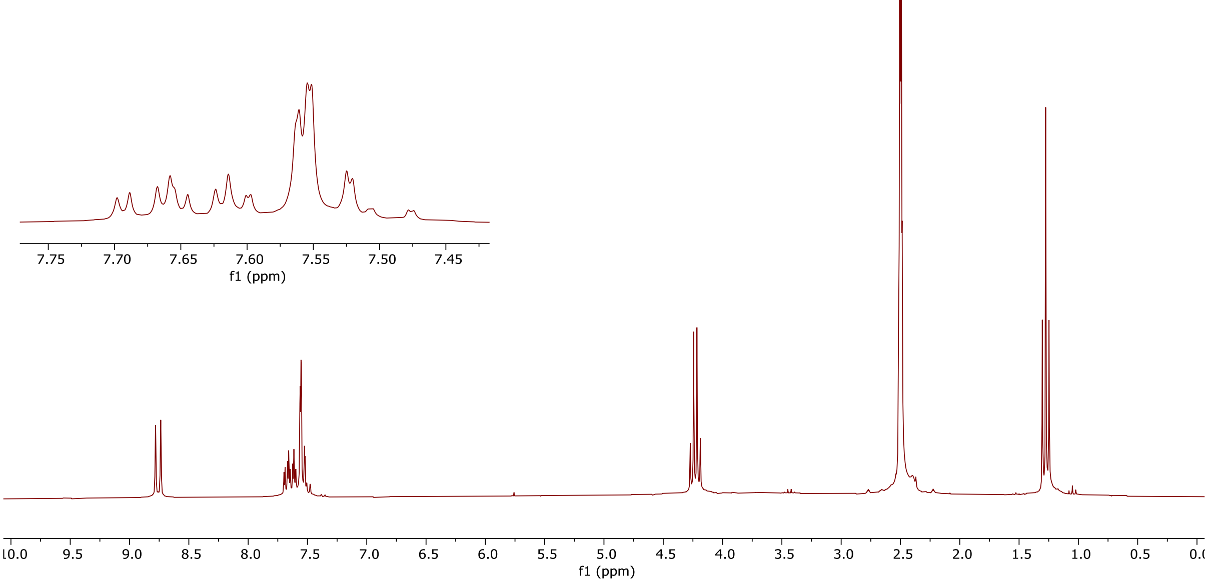

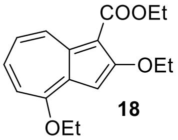

18

^{1}H-NMR, 250 MHz, $CDCl_3$

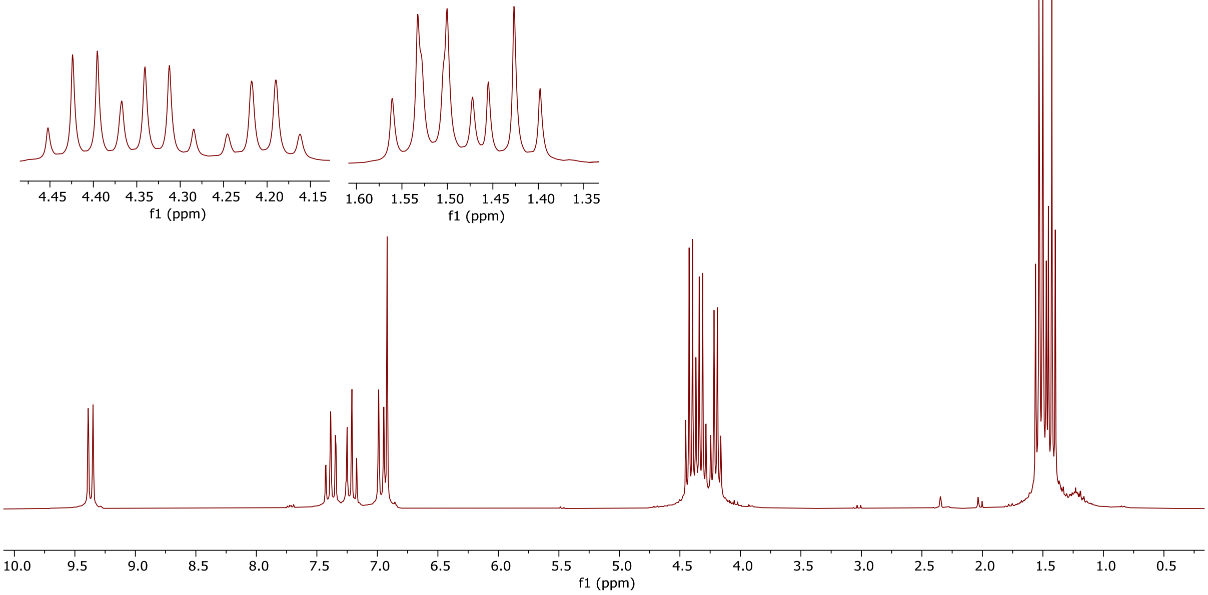

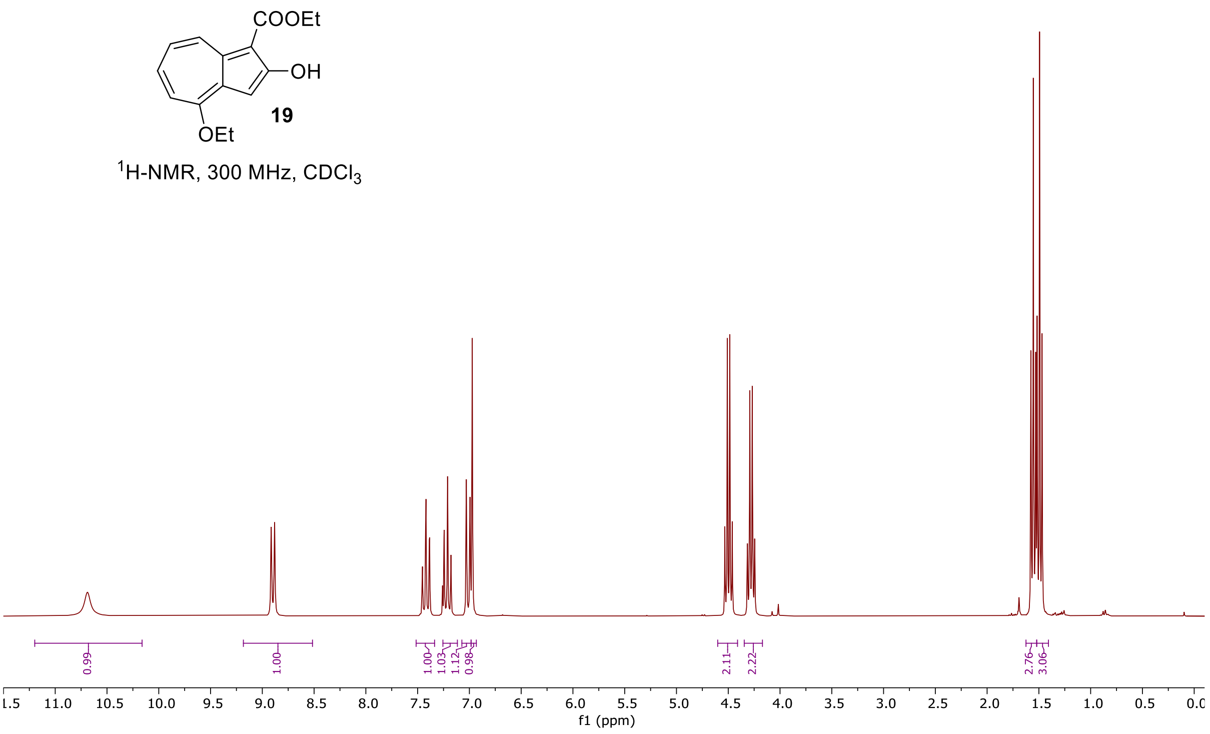
COOEt
OH
OEt
19
^{1}H-NMR, 300 MHz, CDCl$_3$
0.99
1.00
1.00
1.03
1.12
0.98
2.11
2.22
2.76
3.06
f1 (ppm)

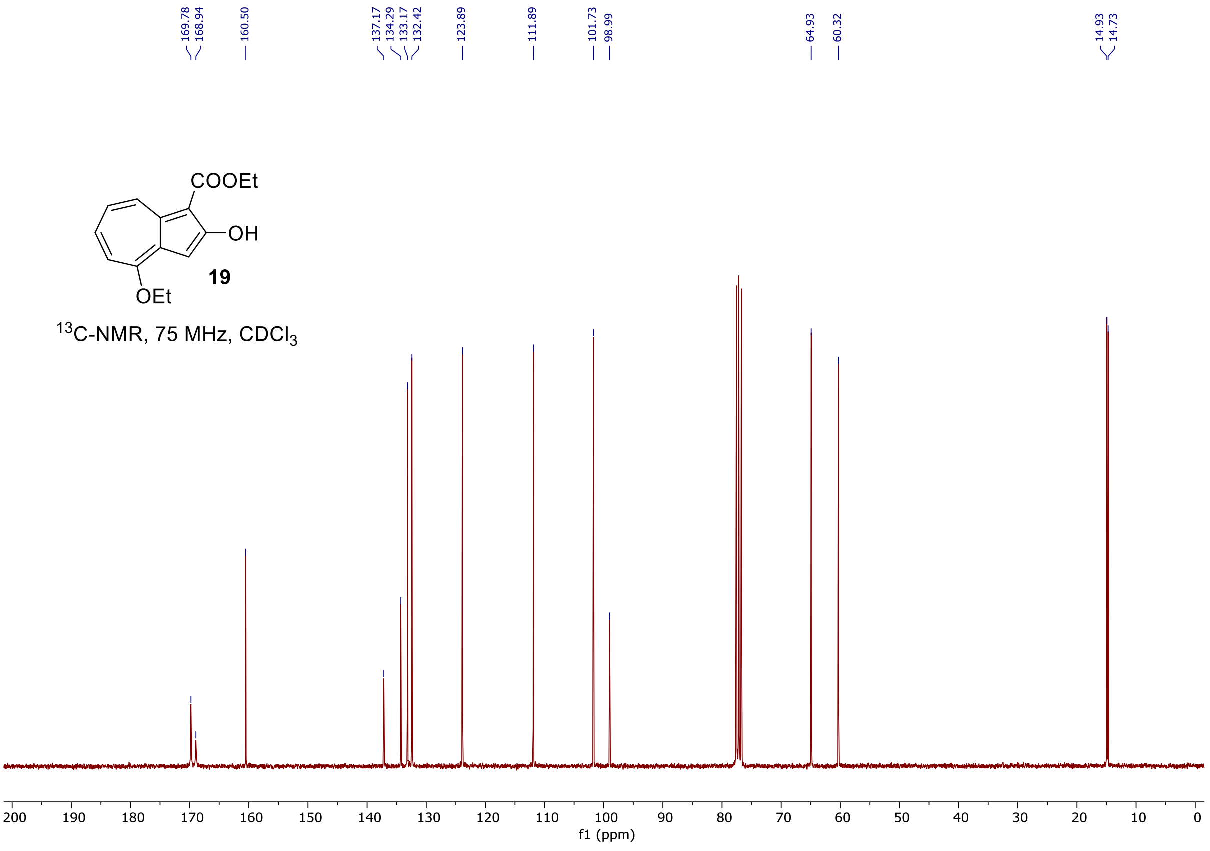
169.78
168.94
160.50
137.17
134.29
133.17
132.42
123.89
111.89
101.73
98.99
64.93
60.32
14.93
14.73
COOEt
OH
OEt
19
^{13}C-NMR, 75 MHz, CDCl$_3$
f1 (ppm)

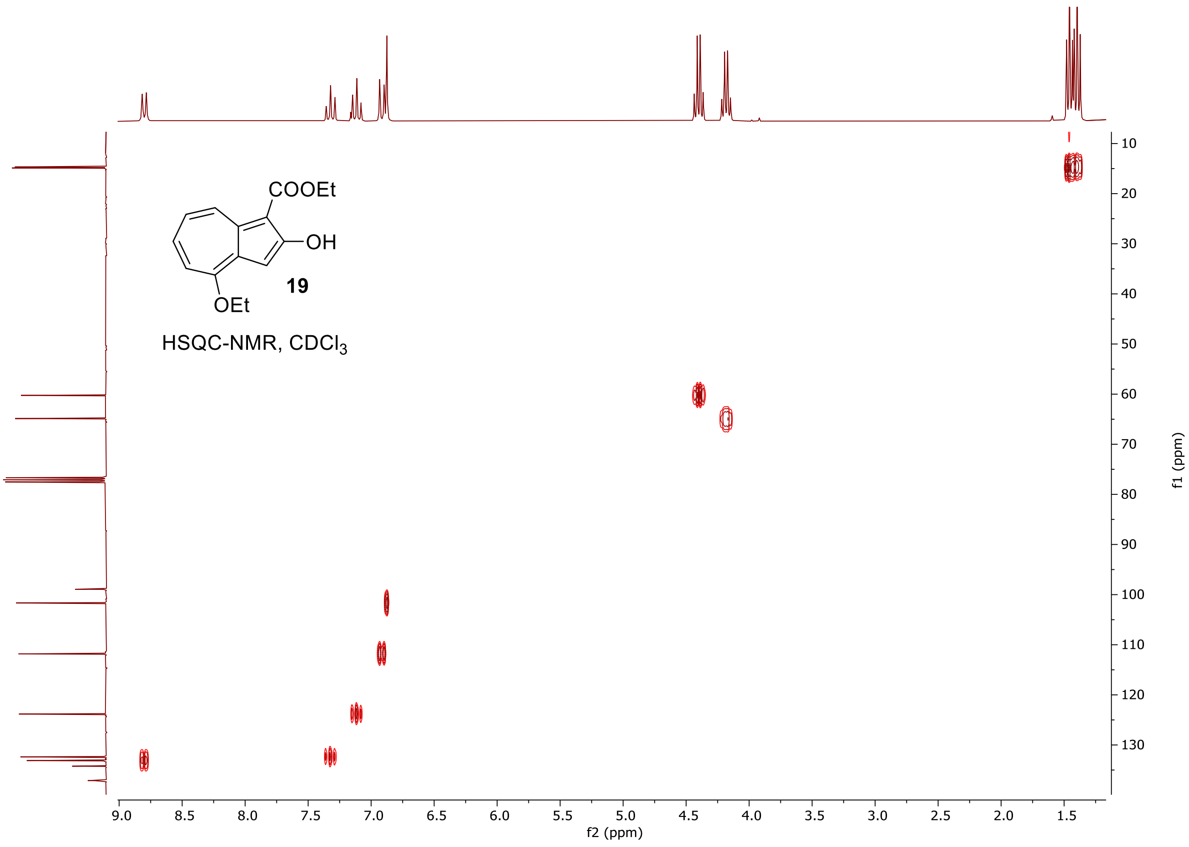
COOEt
OH
19
OEt
HSQC-NMR, CDCl3
f2 (ppm)
f1 (ppm)

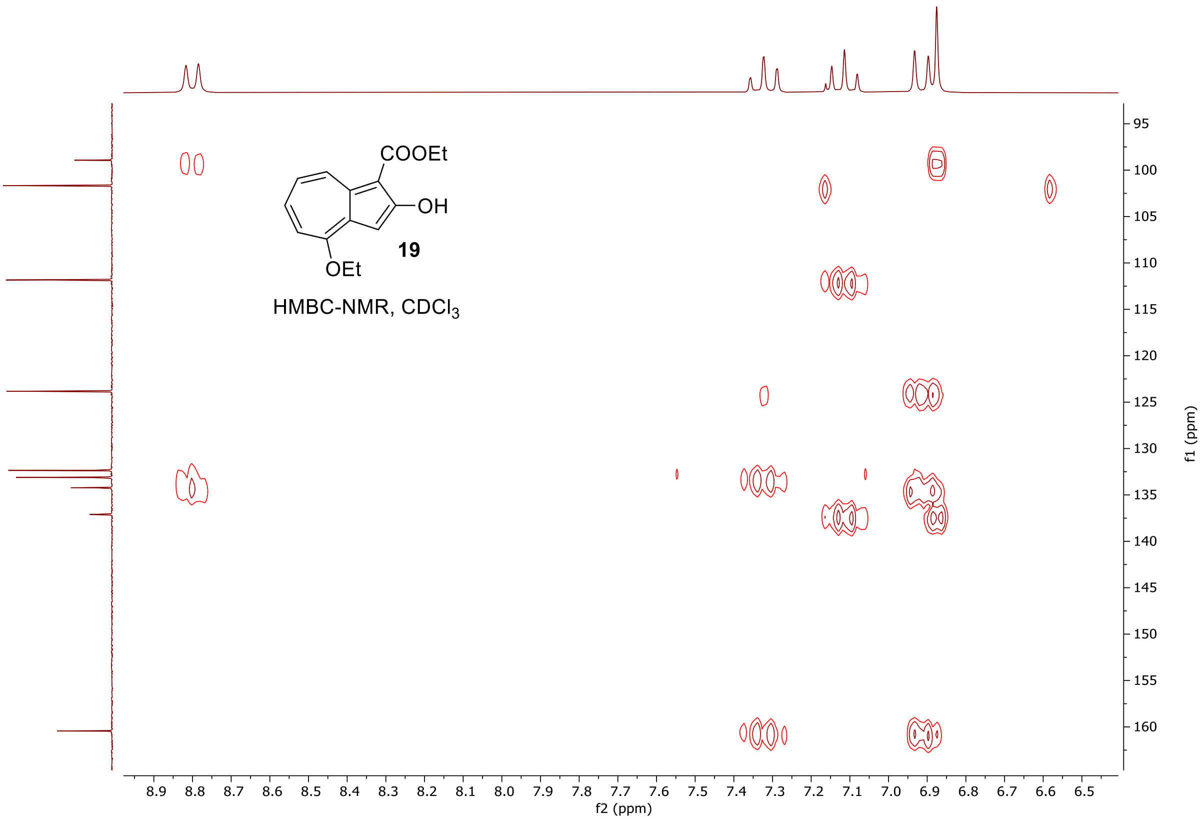
COOEt
OH
19
OEt
HMBC-NMR, CDCl3
f2 (ppm)
f1 (ppm)

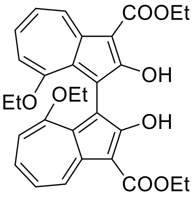

rac-**20**
1,1'-BAzOL

^{1}H-NMR, 500 MHz, $CDCl_3$

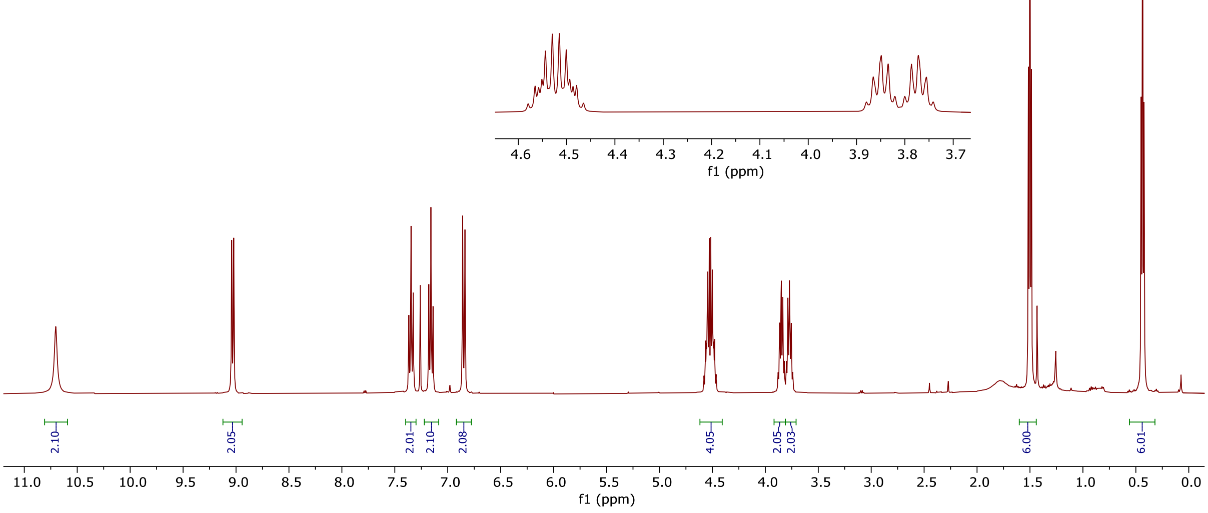

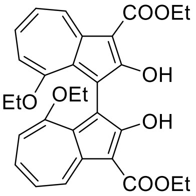

rac-**20**
1,1'-BAzOL

^{13}C-NMR, 126 MHz, $CDCl_3$

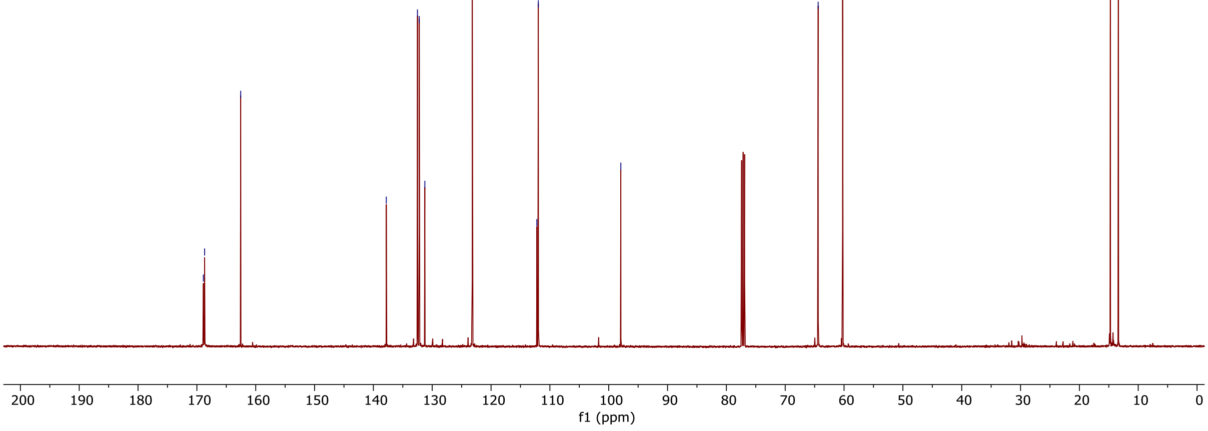

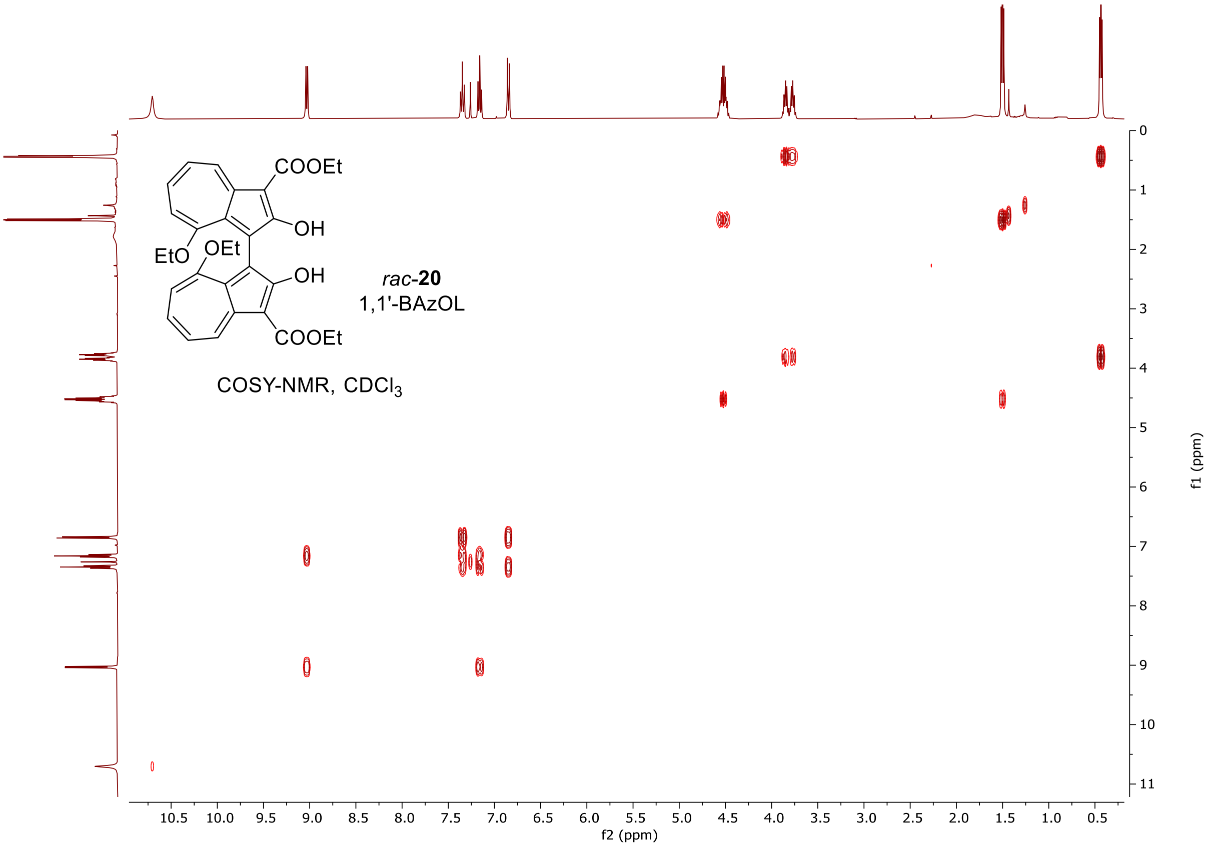
COOEt
OH
EtO
OEt
OH
COOEt
rac-20
1,1'-BAzOL
COSY-NMR, CDCl3
f2 (ppm)
f1 (ppm)

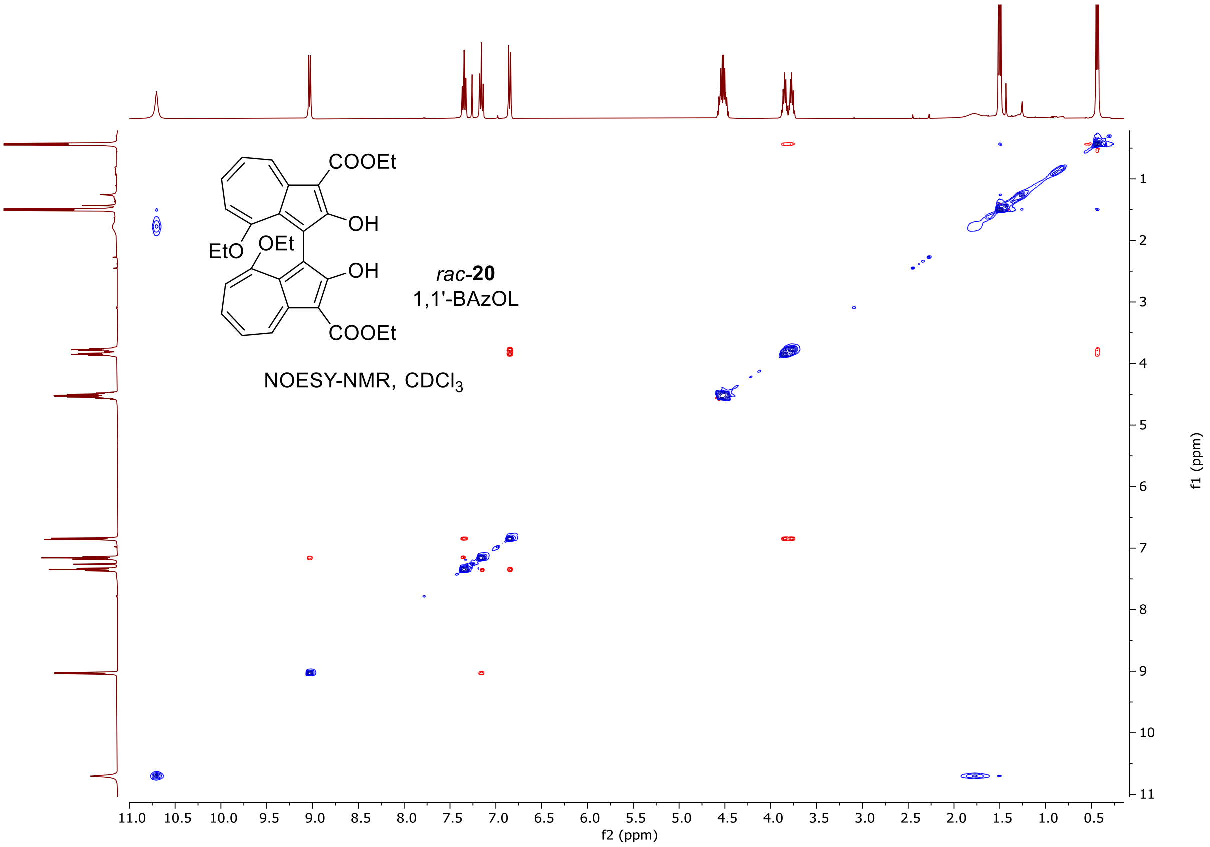
COOEt
OH
EtO
OEt
OH
COOEt
rac-20
1,1'-BAzOL
NOESY-NMR, CDCl3
f2 (ppm)
f1 (ppm)

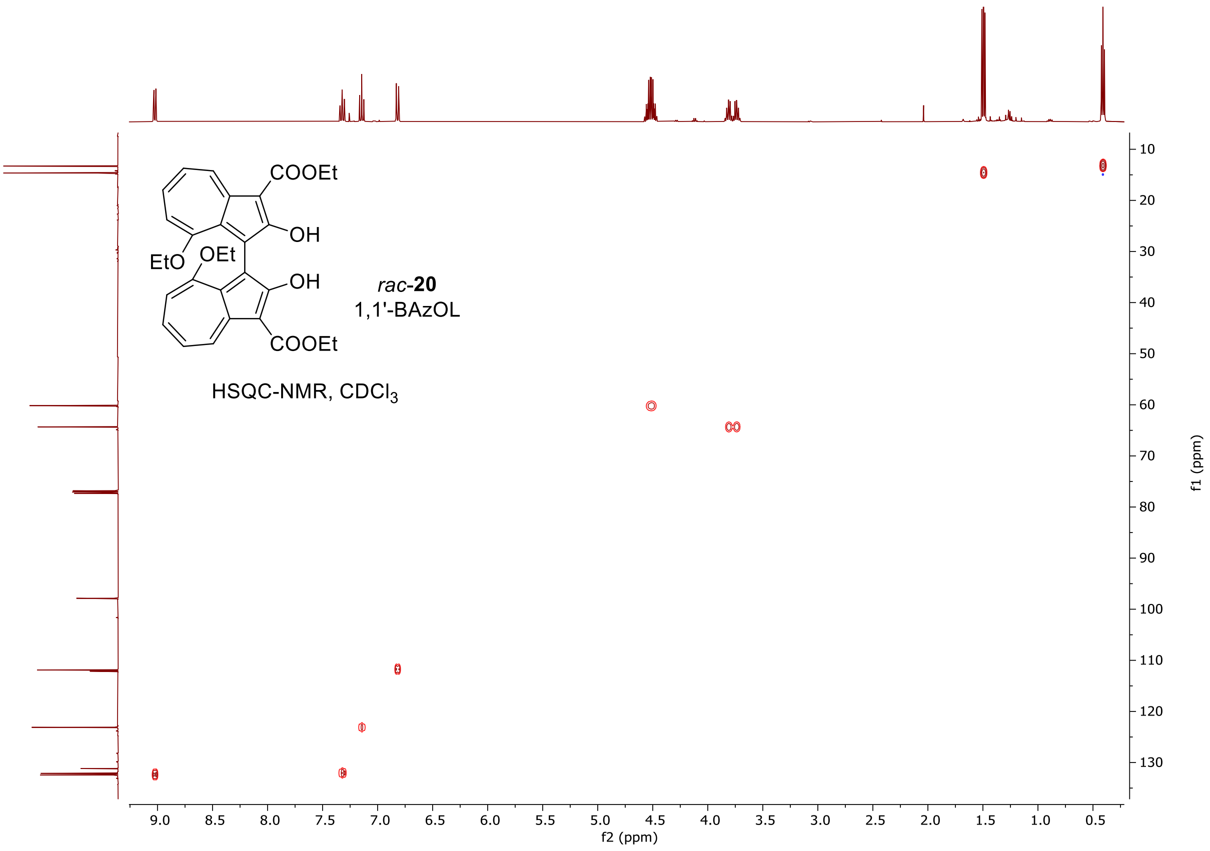
COOEt
OH
EtO
OEt
OH
COOEt
rac-20
1,1'-BAzOL
HSQC-NMR, CDCl3
f2 (ppm)
f1 (ppm)

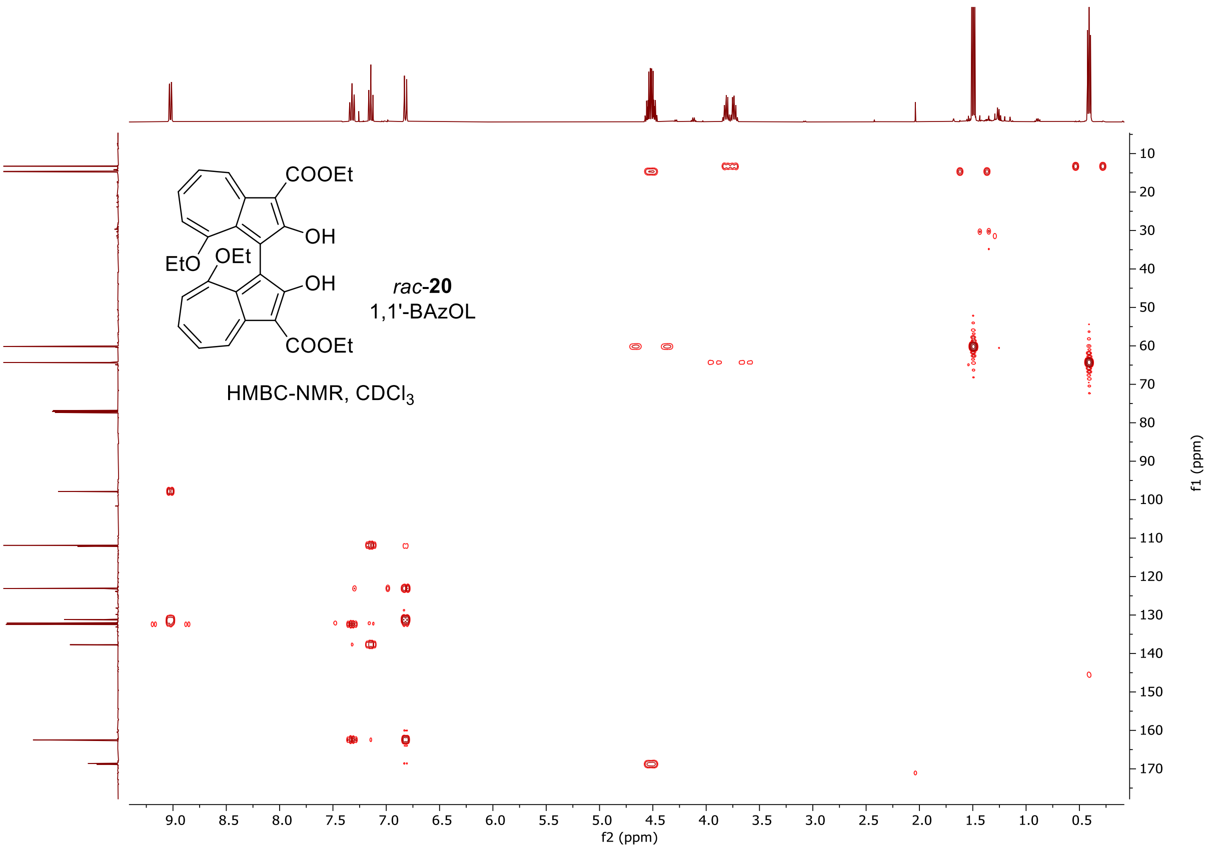
COOEt
OH
EtO
OEt
OH
COOEt
rac-20
1,1'-BAzOL
HMBC-NMR, CDCl3
f2 (ppm)
f1 (ppm)

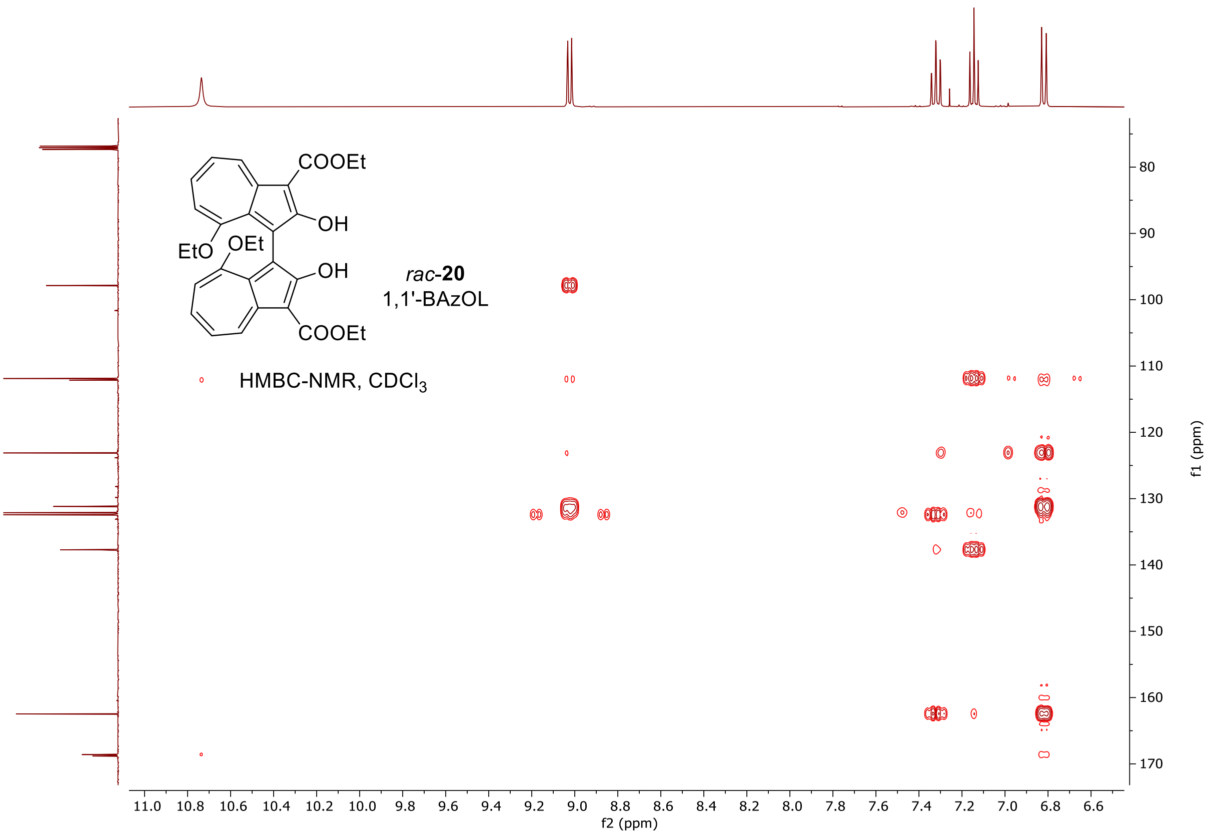
COOEt
OH
EtO
OEt
OH
COOEt
rac-**20**
1,1'-BAzOL
HMBC-NMR, $CDCl_3$
f1 (ppm)
f2 (ppm)
80
90
100
110
120
130
140
150
160
170
11.0
10.8
10.6
10.4
10.2
10.0
9.8
9.6
9.4
9.2
9.0
8.8
8.6
8.4
8.2
8.0
7.8
7.6
7.4
7.2
7.0
6.8
6.6

(R_a,1R,2S,5R)-**22**

^{1}H-NMR, 500 MHz, $CDCl_3$

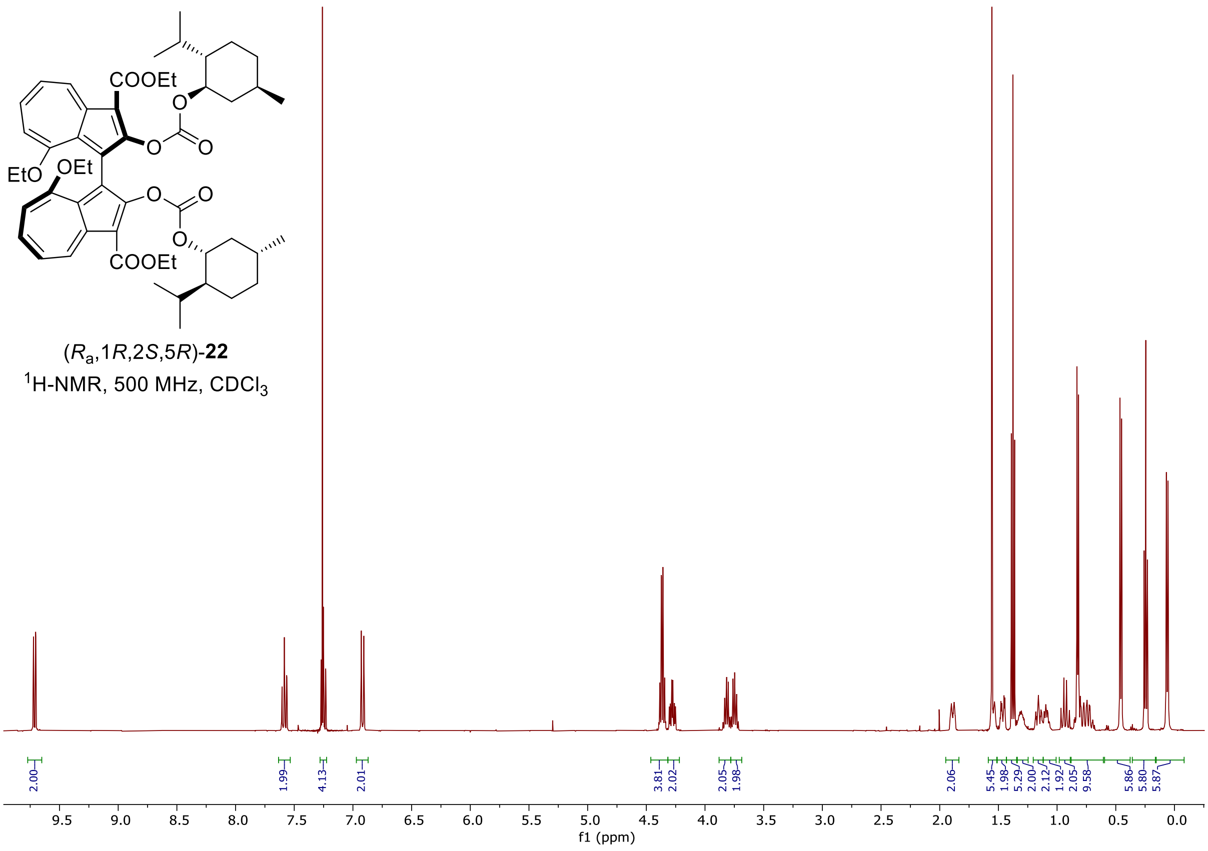

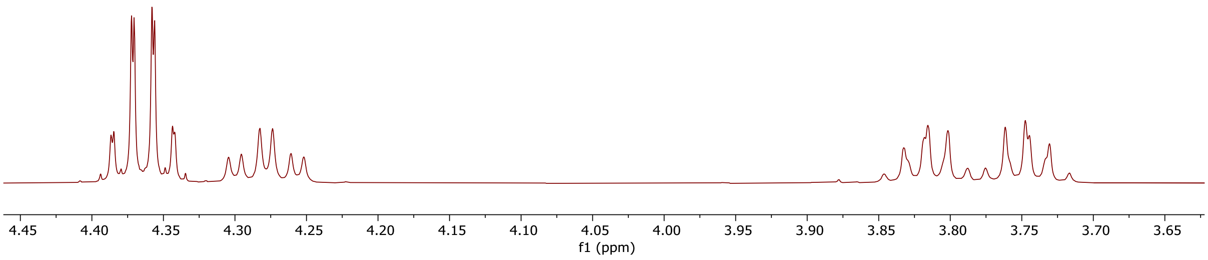

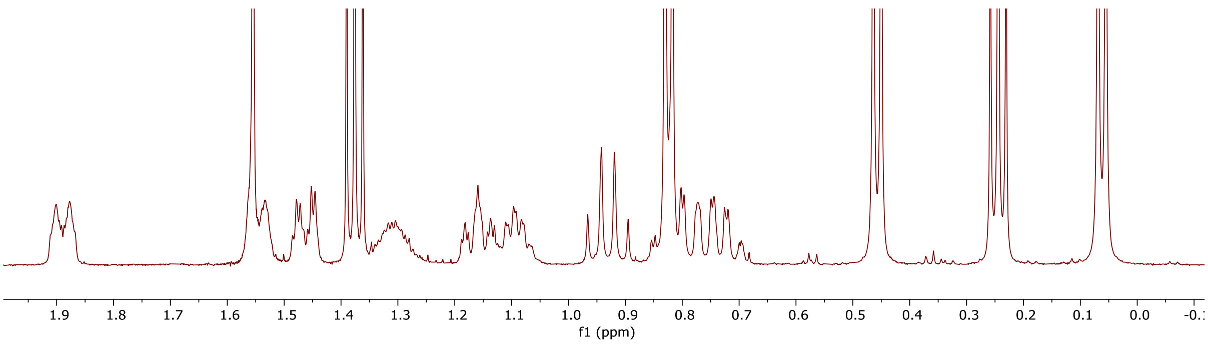

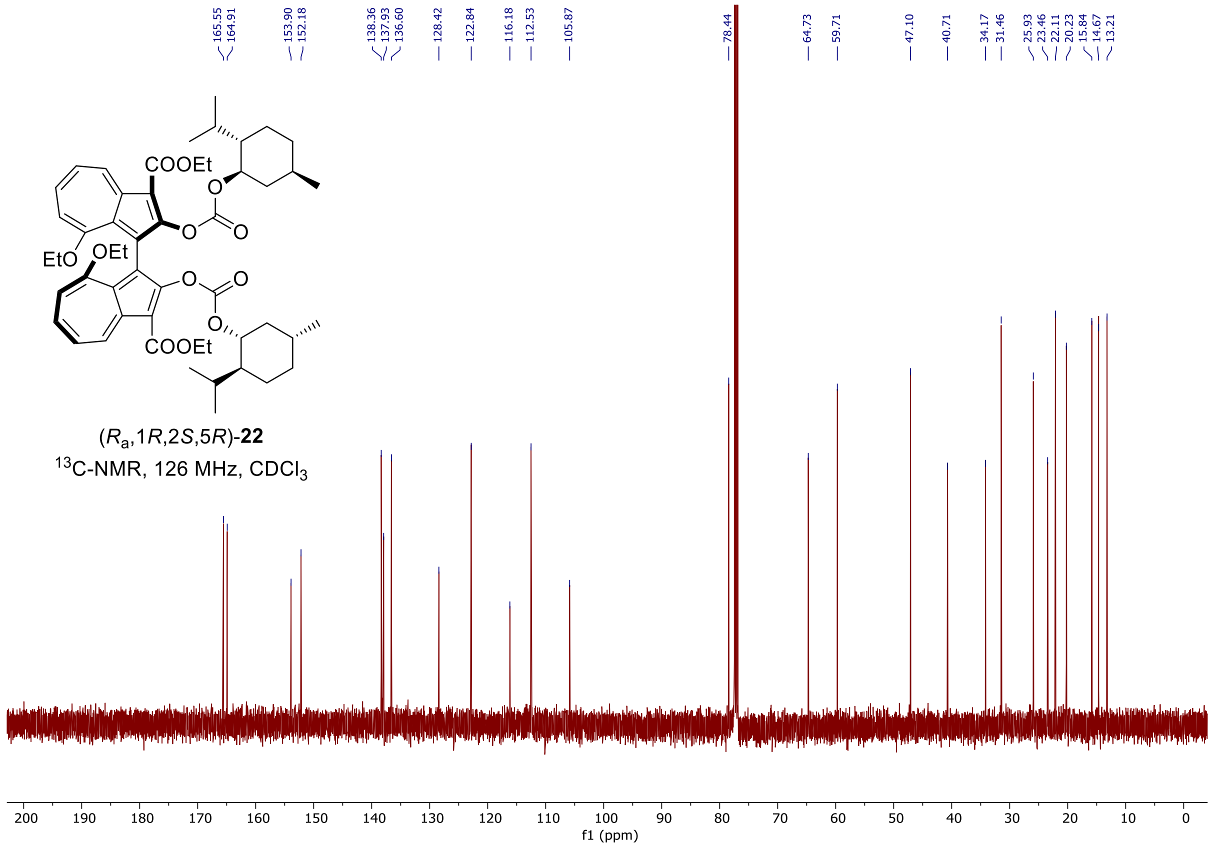
165.55
164.91
153.90
152.18
138.36
137.93
136.60
128.42
122.84
116.18
112.53
105.87
78.44
64.73
59.71
47.10
40.71
34.17
31.46
25.93
23.46
22.11
20.23
15.84
14.67
13.21
COOEt
O
O
O
EtO
OEt
O
O
O
COOEt
(R_a,1R,2S,5R)-**22**
^{13}C-NMR, 126 MHz, $CDCl_3$
f1 (ppm)

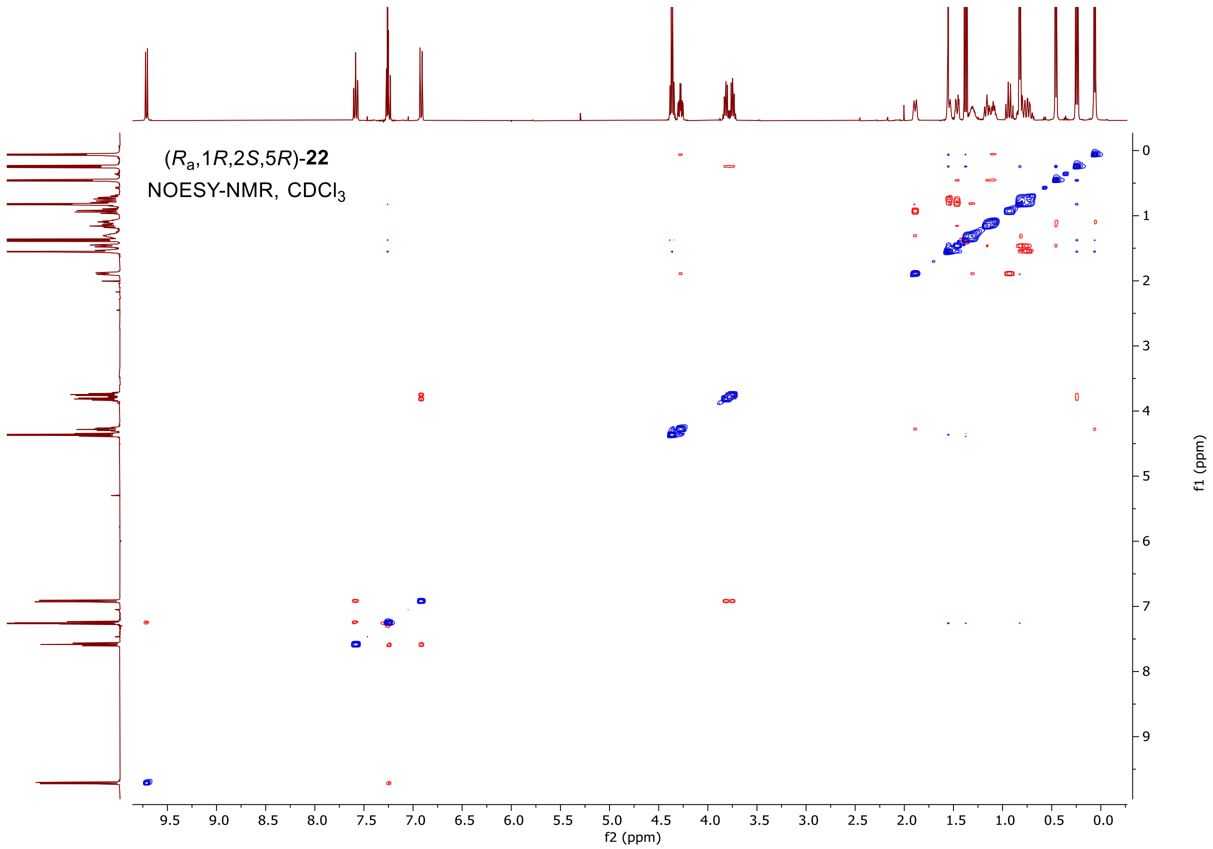
(R_a,1R,2S,5R)-**22**
NOESY-NMR, $CDCl_3$
f2 (ppm)
f1 (ppm)

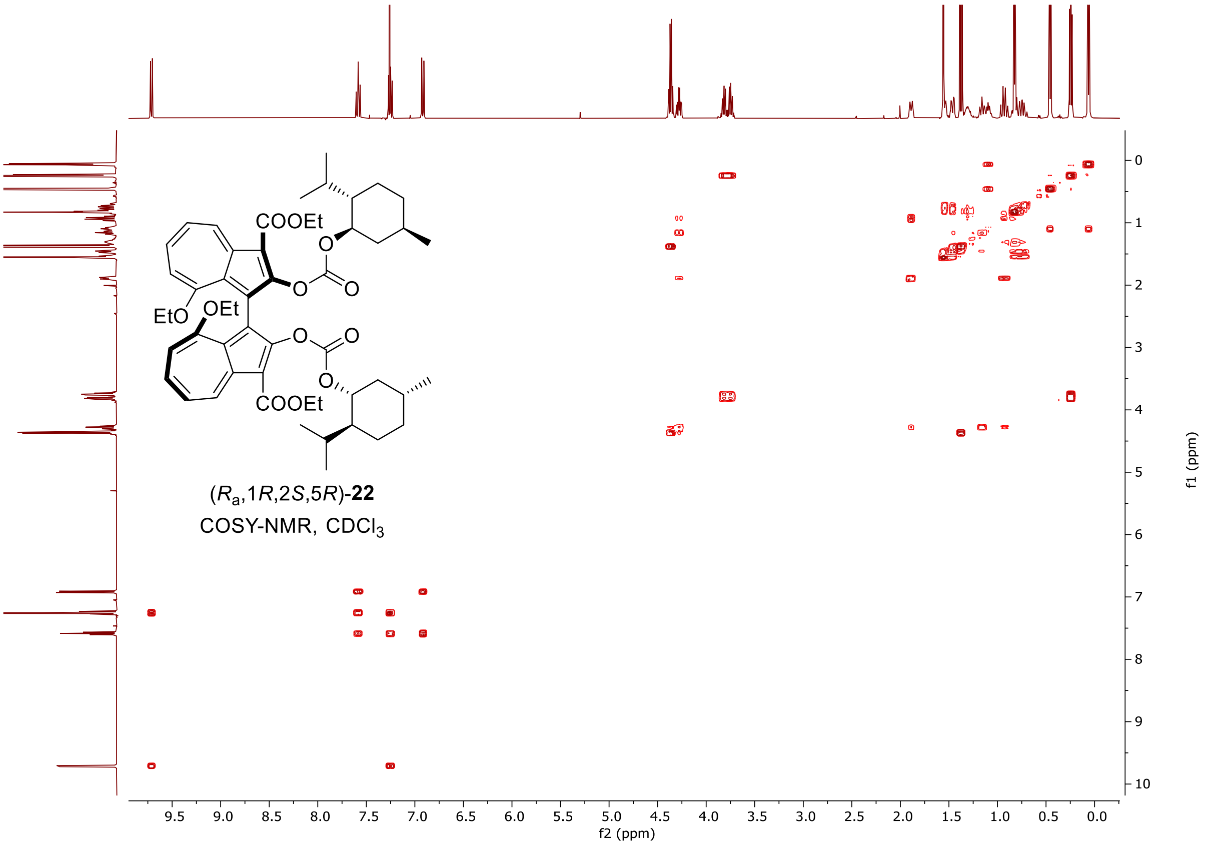

(R_a,1R,2S,5R)-**22**
COSY-NMR, $CDCl_3$

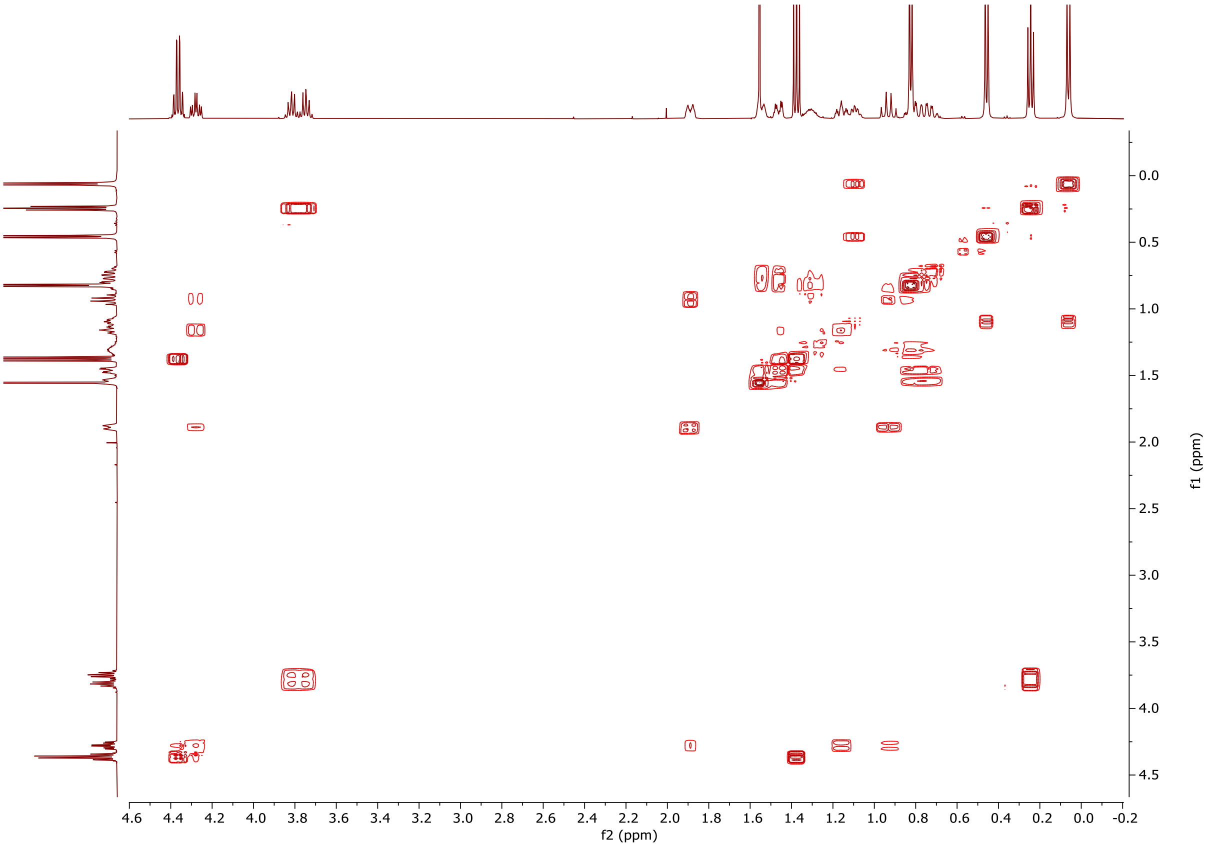

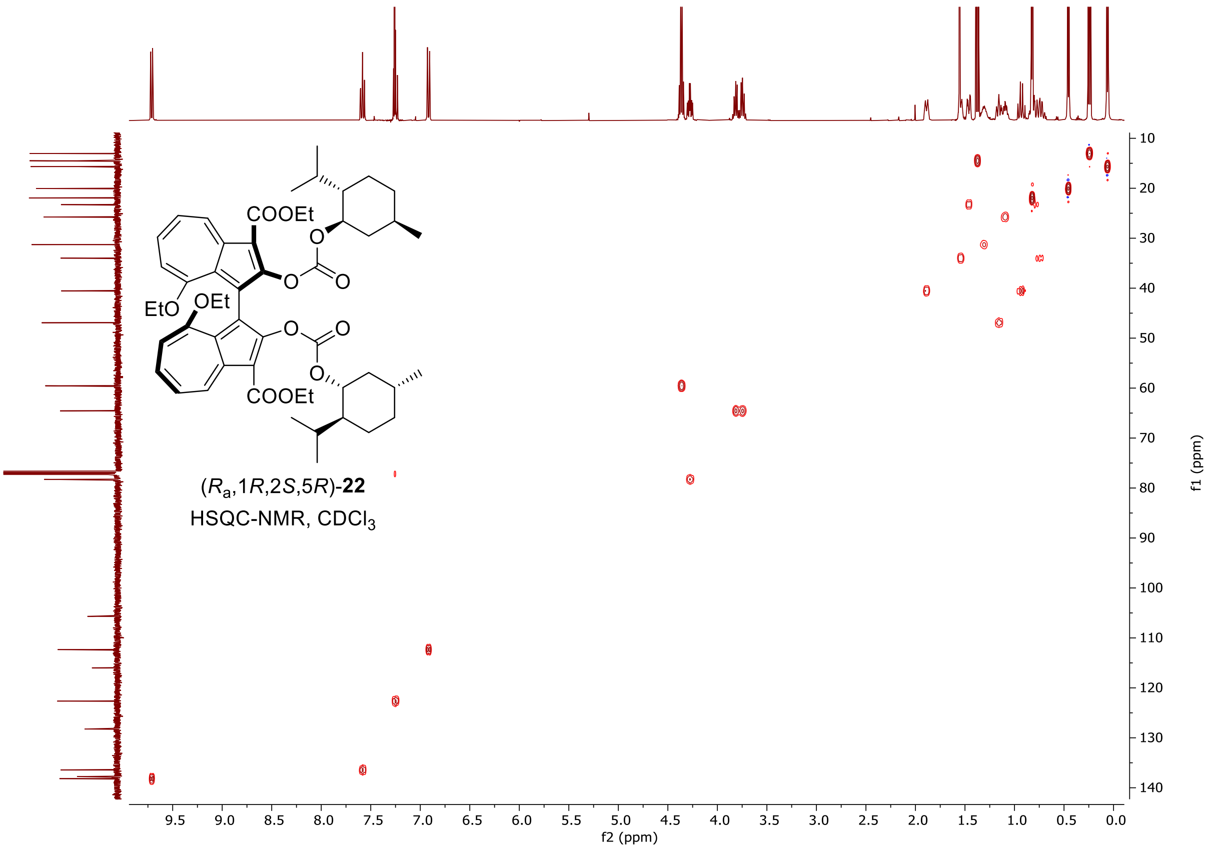
COOEt
EtO
OEt
O
O
O
O
COOEt
(R_a,1R,2S,5R)-**22**
HSQC-NMR, $CDCl_3$
f2 (ppm)
f1 (ppm)

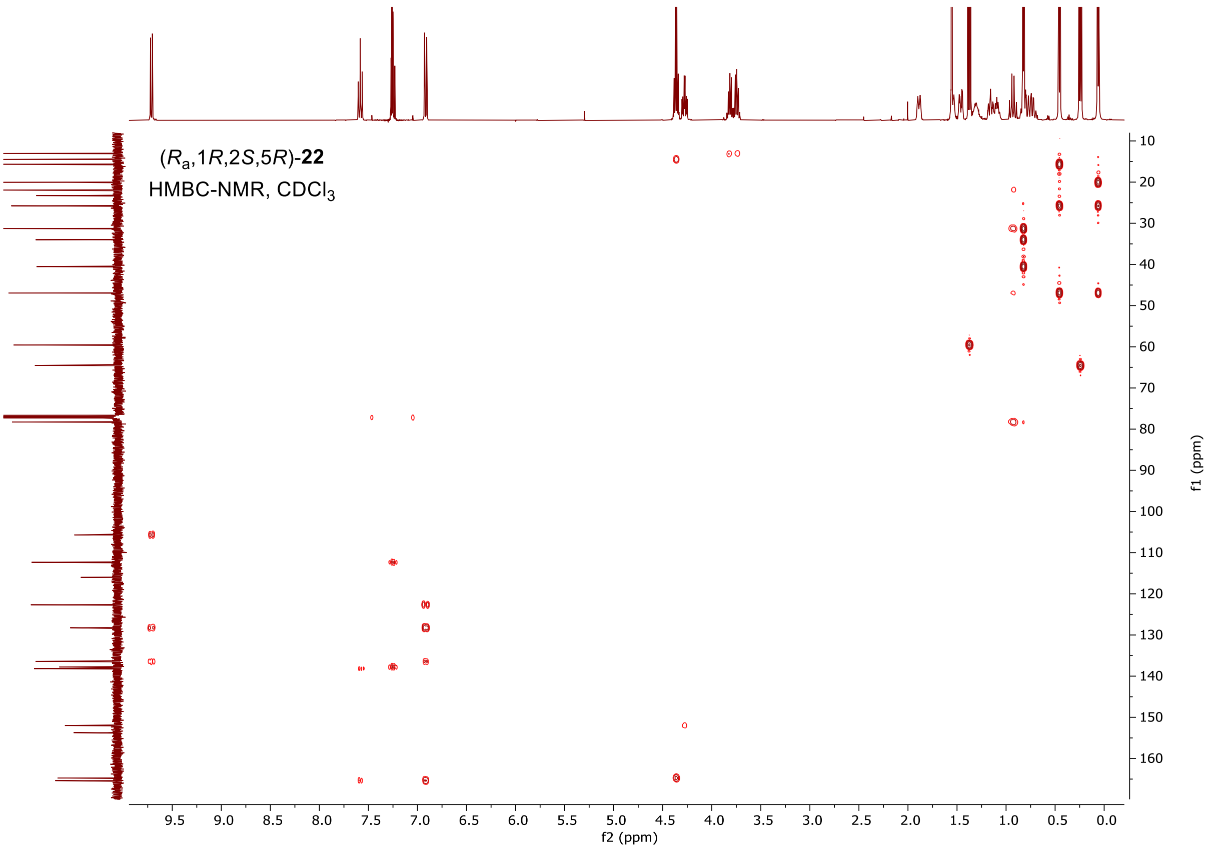
(R_a,1R,2S,5R)-**22**
HMBC-NMR, $CDCl_3$
f2 (ppm)
f1 (ppm)

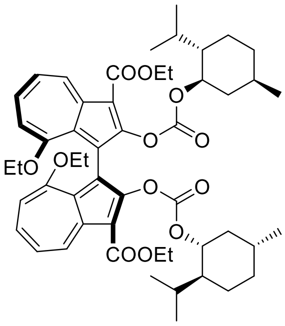

(S_a,1R,2S,5R)-**22**

^{1}H-NMR, 500 MHz, CDCl$_3$

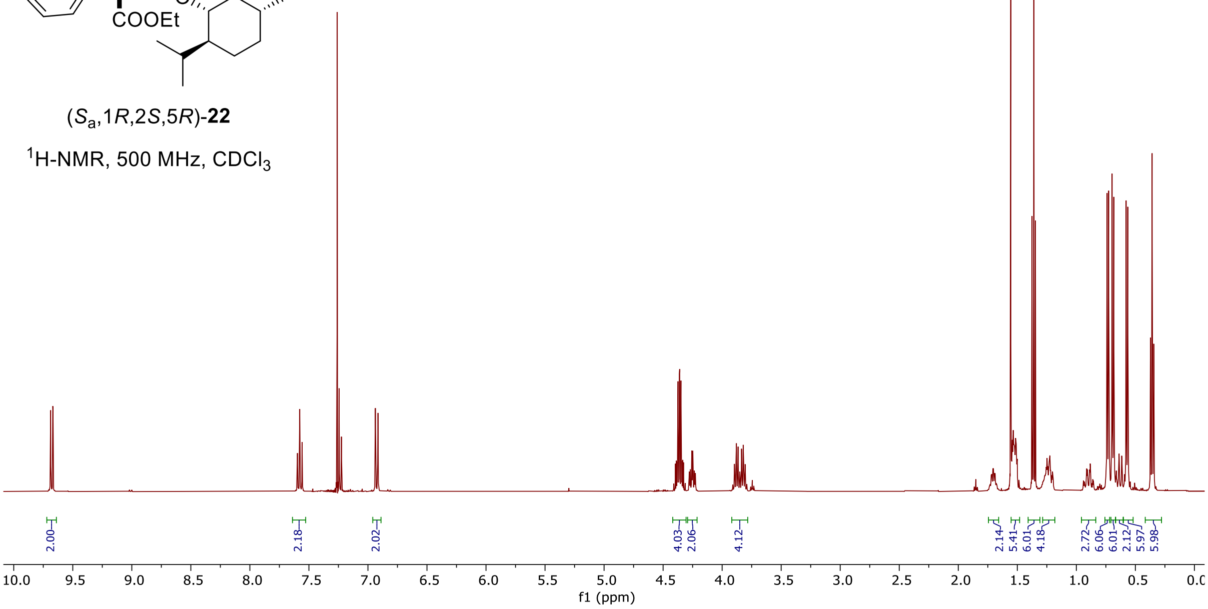

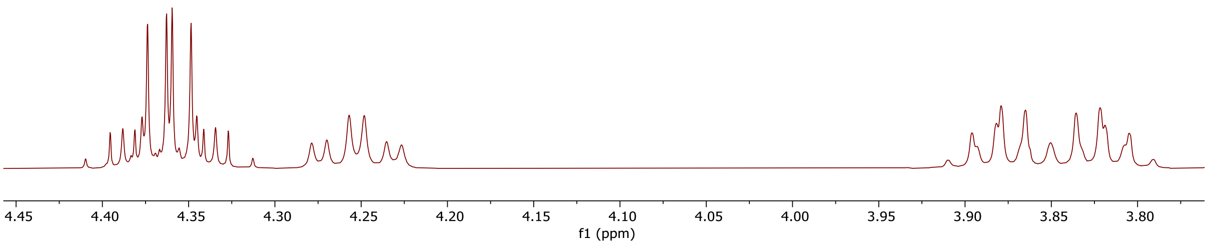

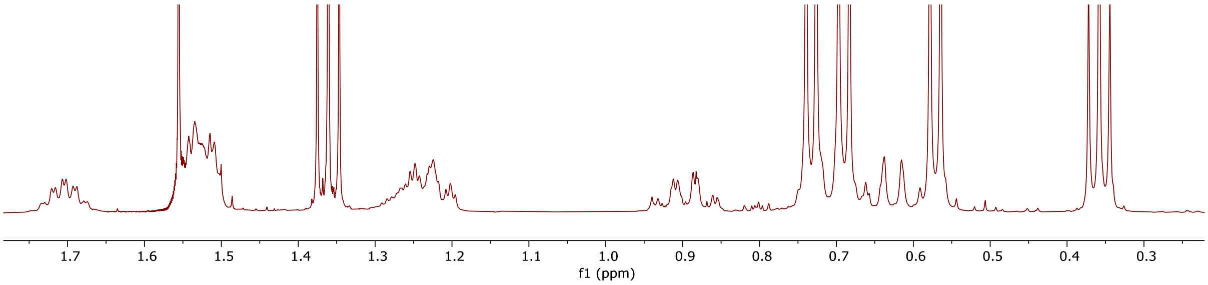

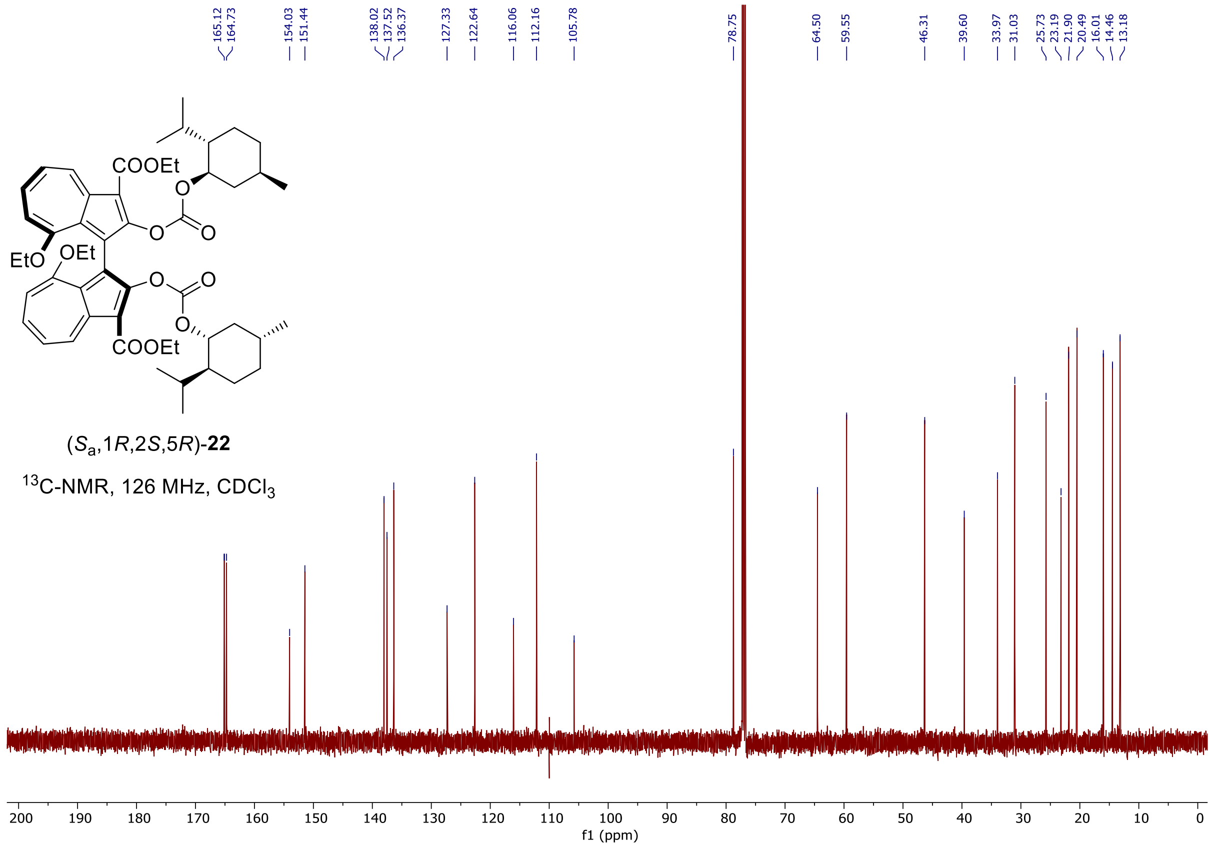

(S_a,1R,2S,5R)-**22**

^{13}C-NMR, 126 MHz, $CDCl_3$

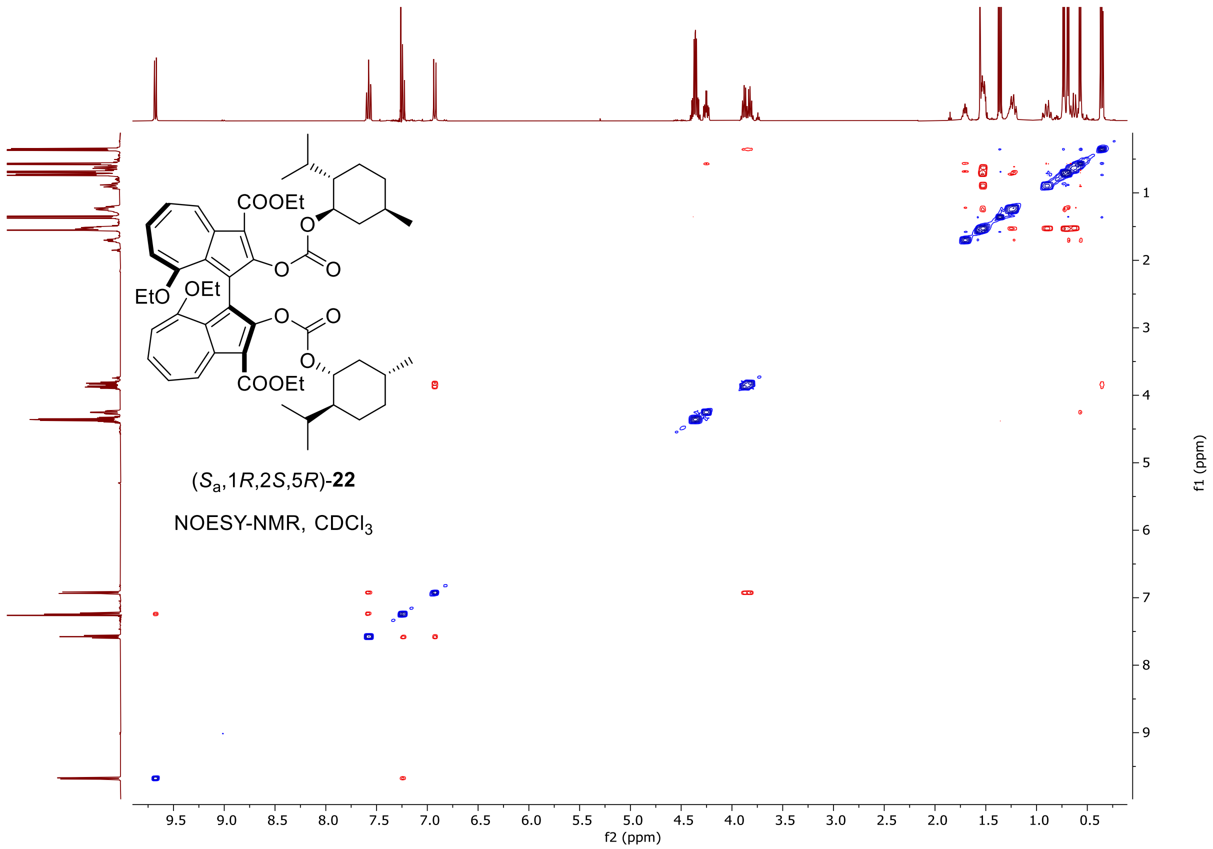

(S_a,1R,2S,5R)-**22**

NOESY-NMR, $CDCl_3$

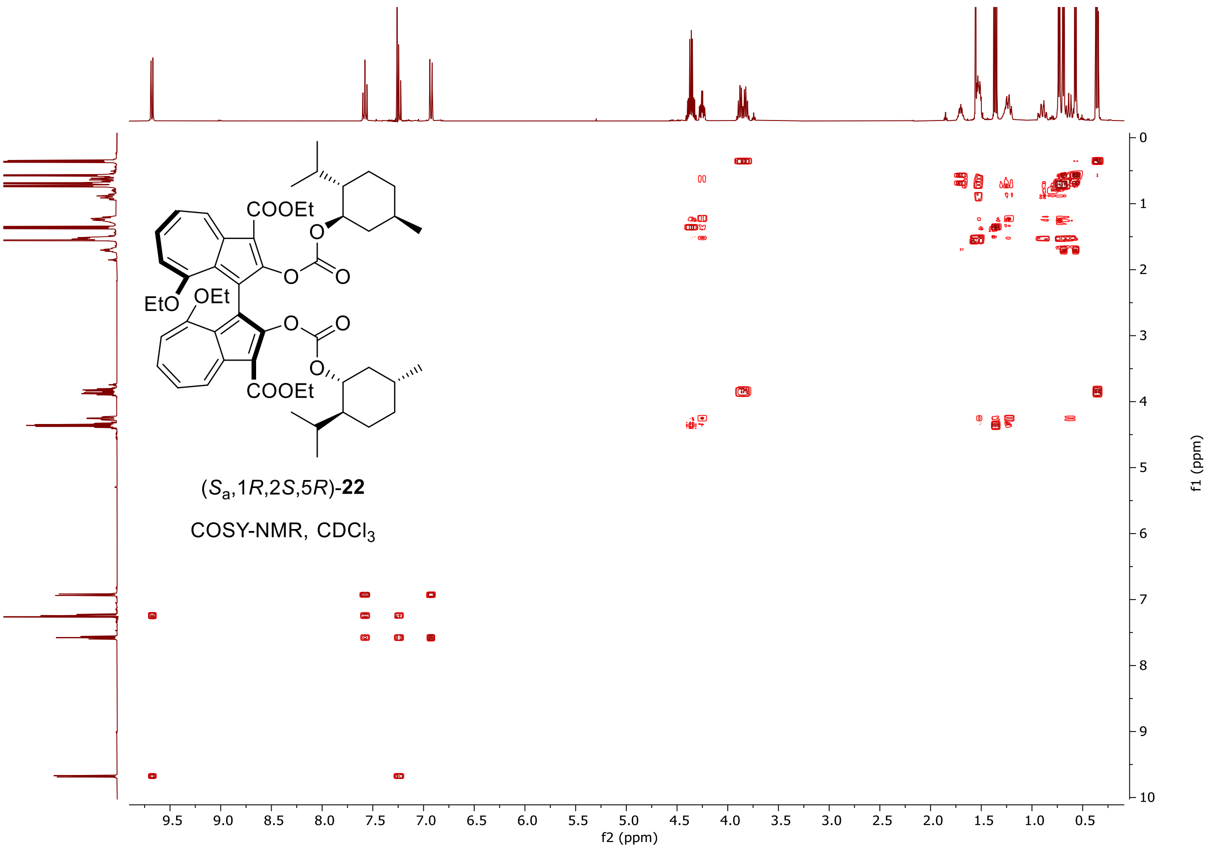
COOEt
COOEt
EtO
OEt
O
(Sa,1R,2S,5R)-22
COSY-NMR, CDCl3
f2 (ppm)
f1 (ppm)

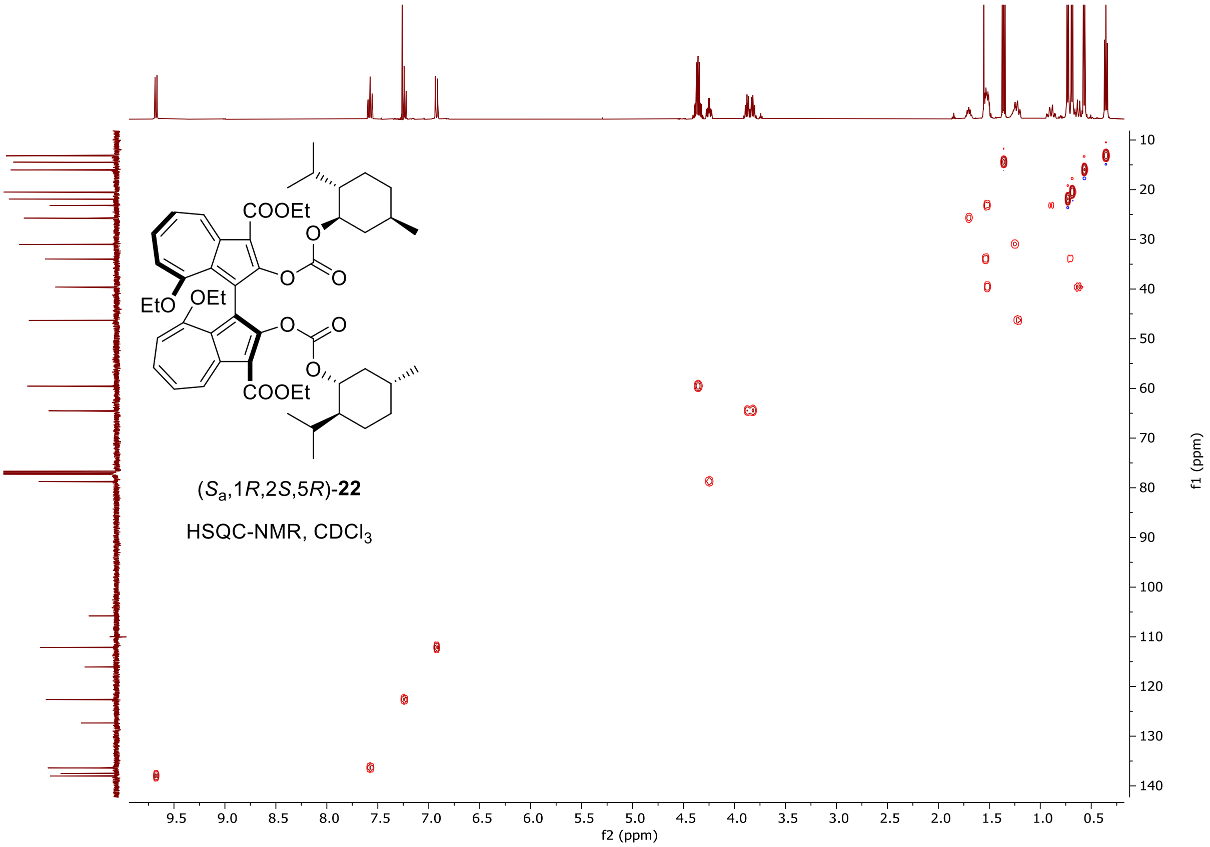
COOEt
COOEt
EtO
OEt
O
(Sa,1R,2S,5R)-22
HSQC-NMR, CDCl3
f2 (ppm)
f1 (ppm)

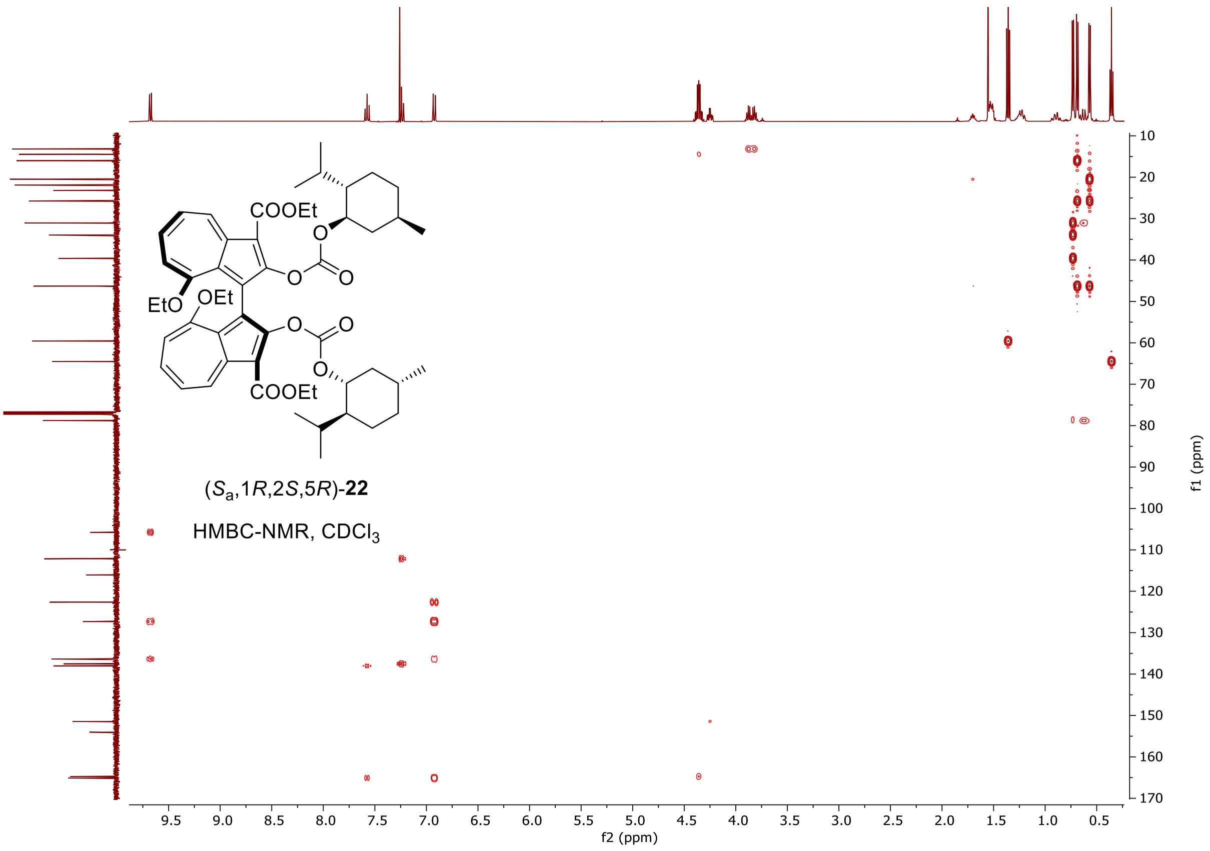
COOEt
O
O
O
EtO
OEt
O
O
O
COOEt
(S_a,1R,2S,5R)-**22**
HMBC-NMR, $CDCl_3$
f1 (ppm)
f2 (ppm)

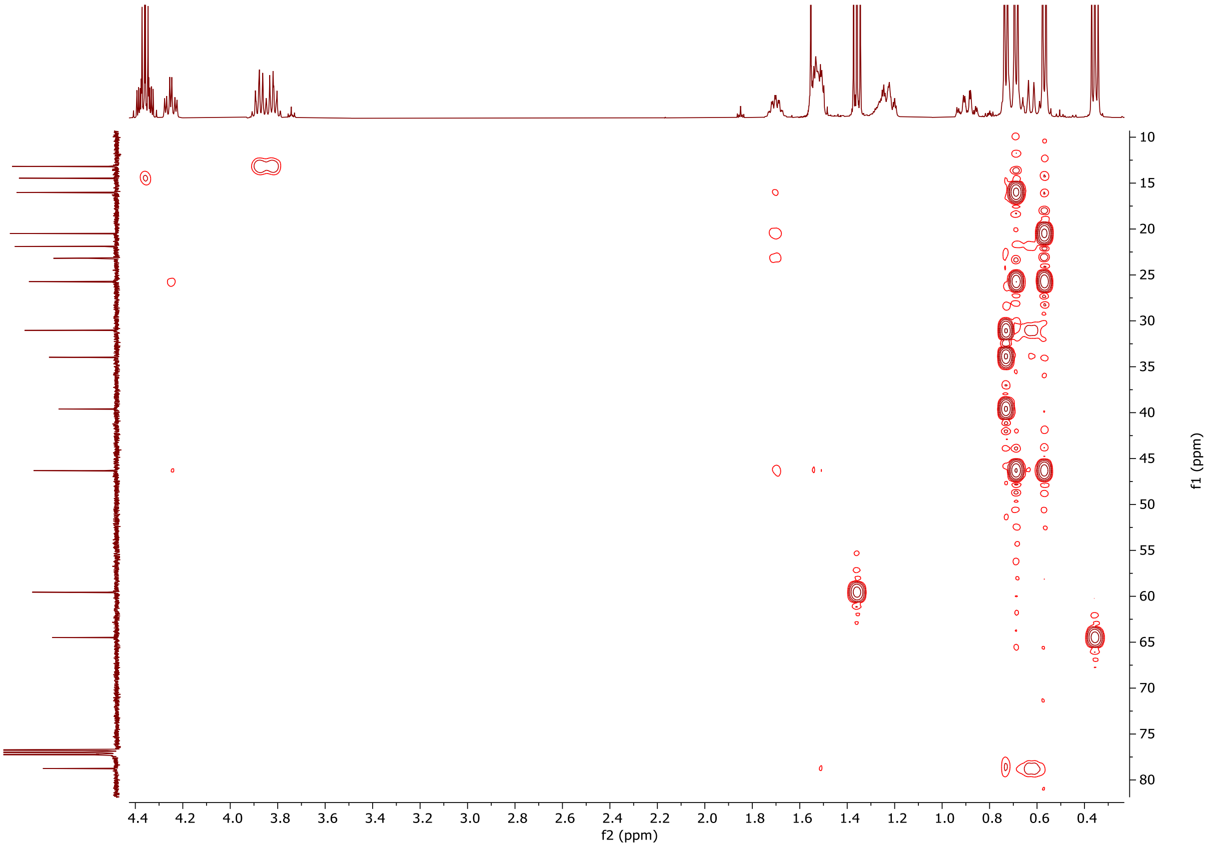
f1 (ppm)
f2 (ppm)

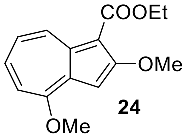

24

^{1}H-NMR, 500 MHz, $CDCl_3$

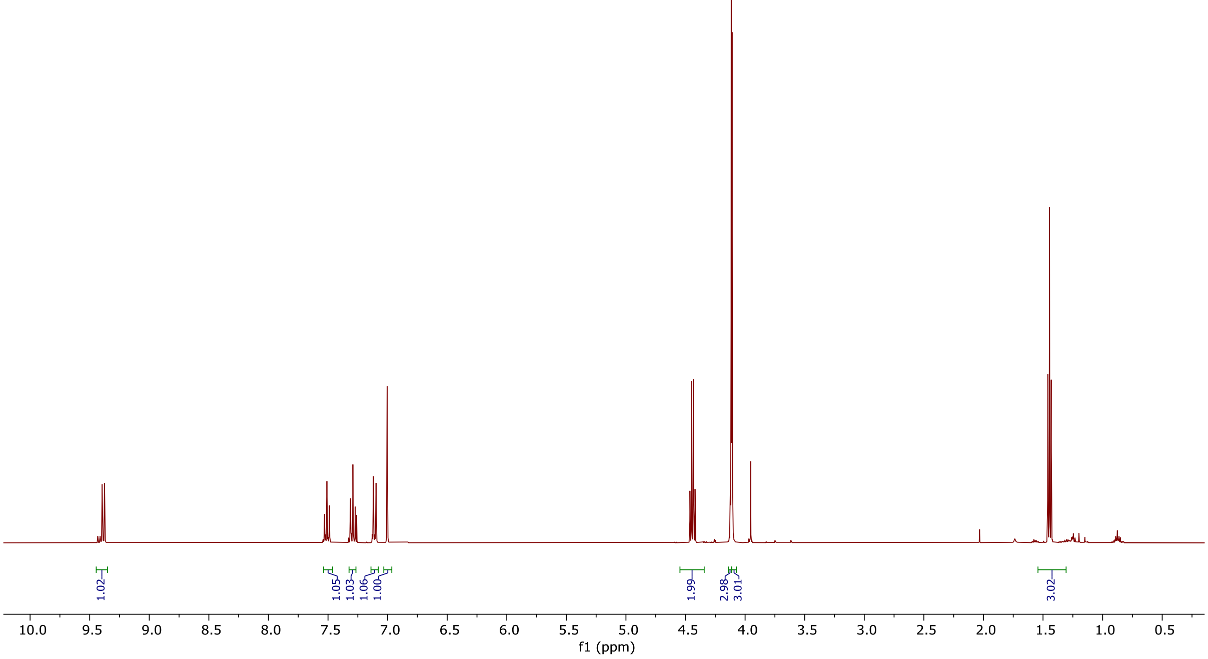

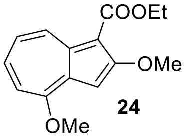

24

^{13}C-NMR, 126 MHz, $CDCl_3$

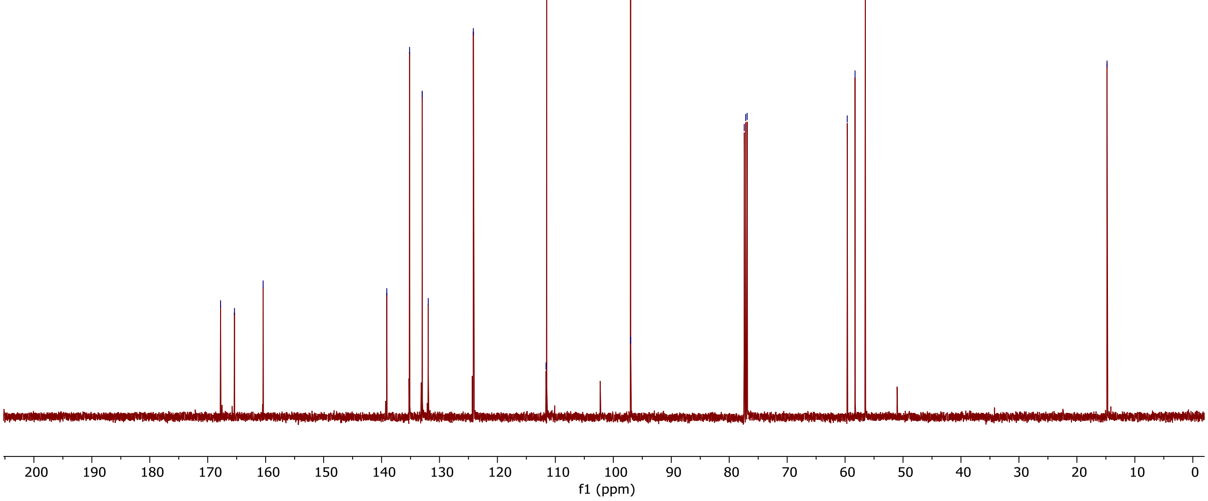

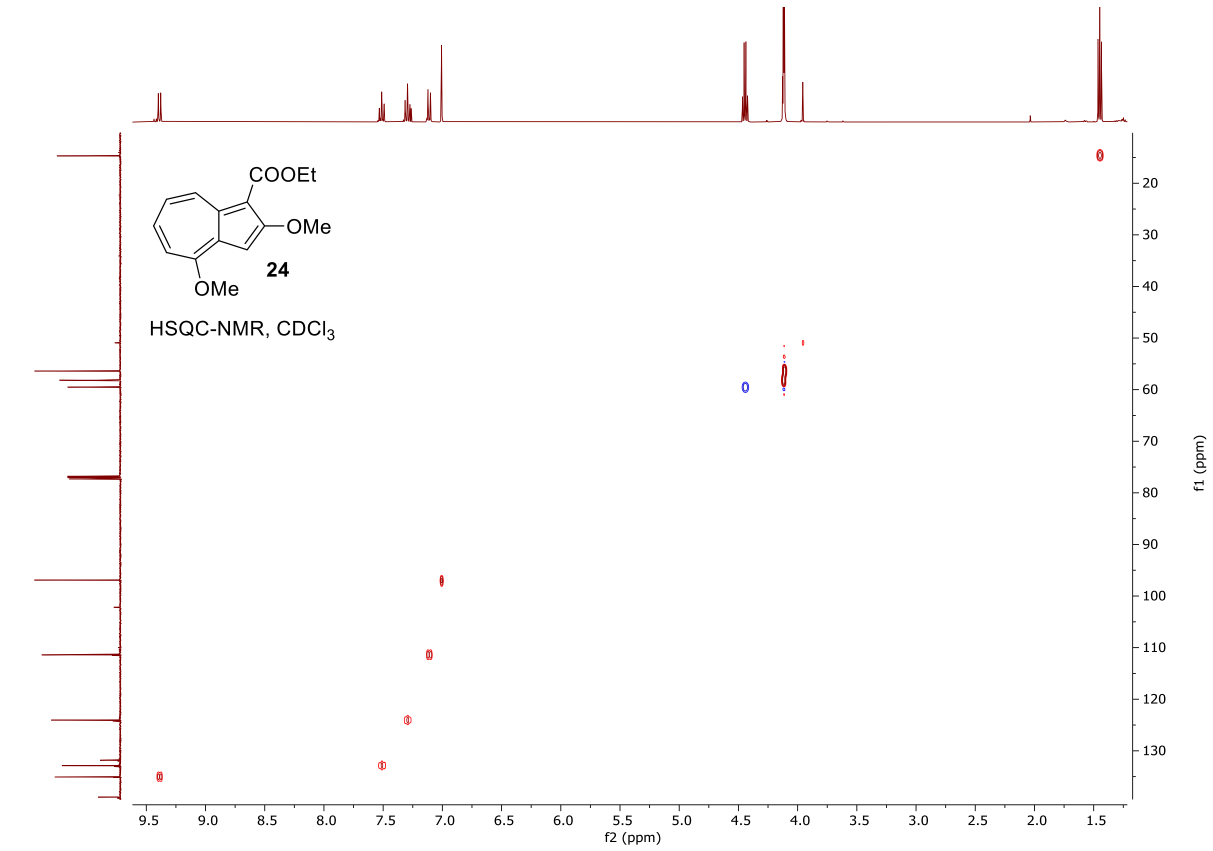
COOEt
OMe
24
OMe
HSQC-NMR, CDCl3
f2 (ppm)
f1 (ppm)

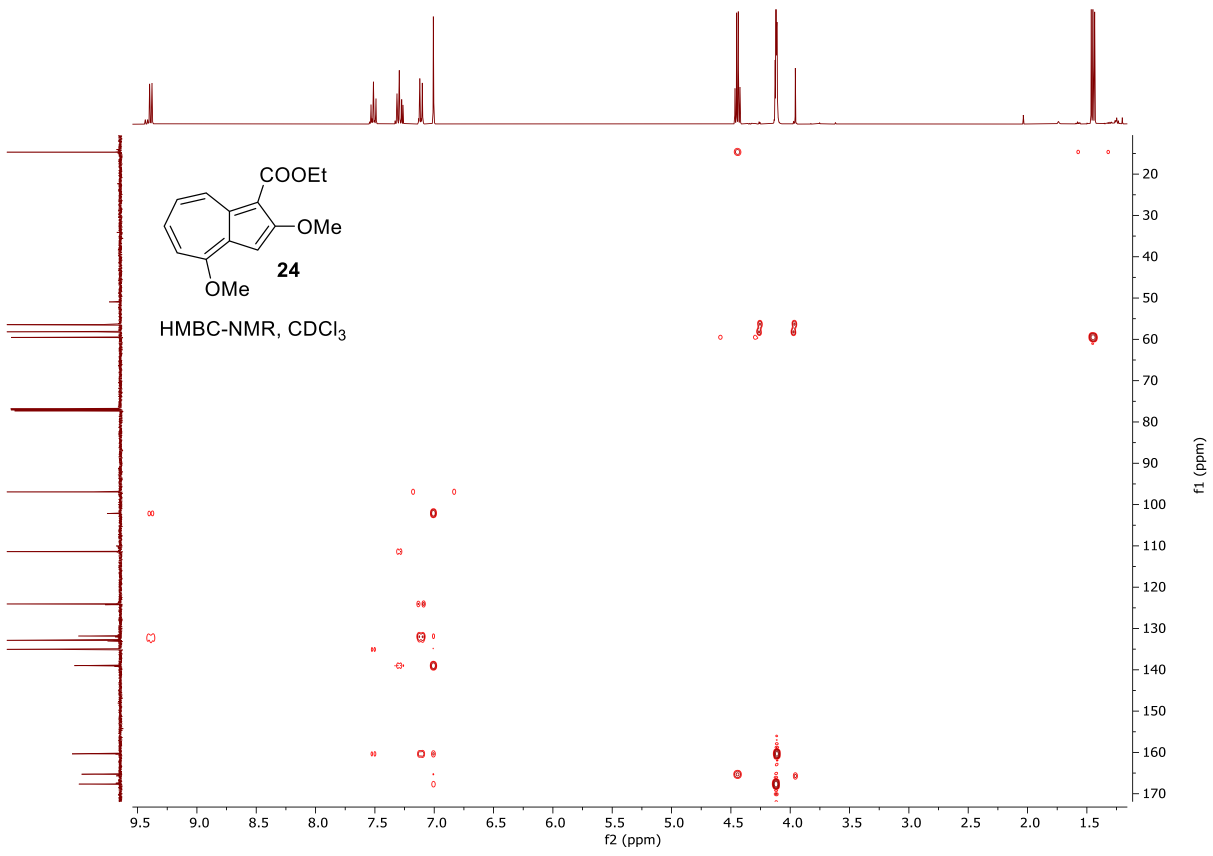
COOEt
OMe
24
OMe
HMBC-NMR, CDCl3
f2 (ppm)
f1 (ppm)

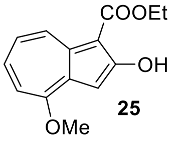

25

^{1}H-NMR, 300 MHz, $CDCl_3$

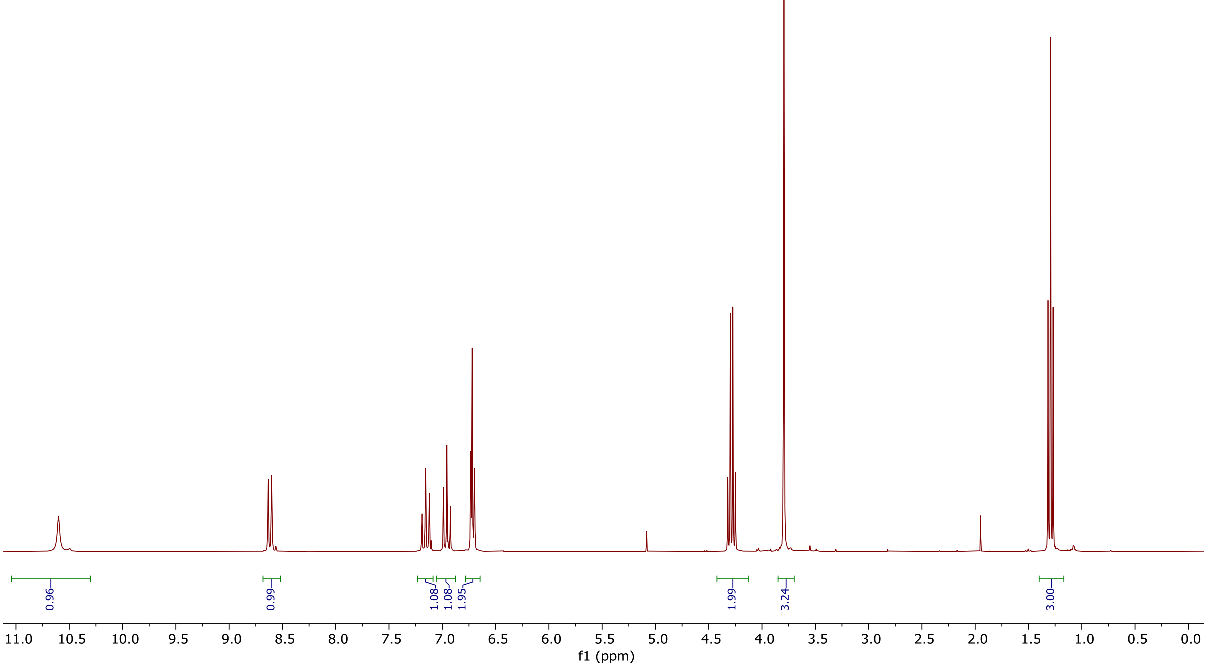

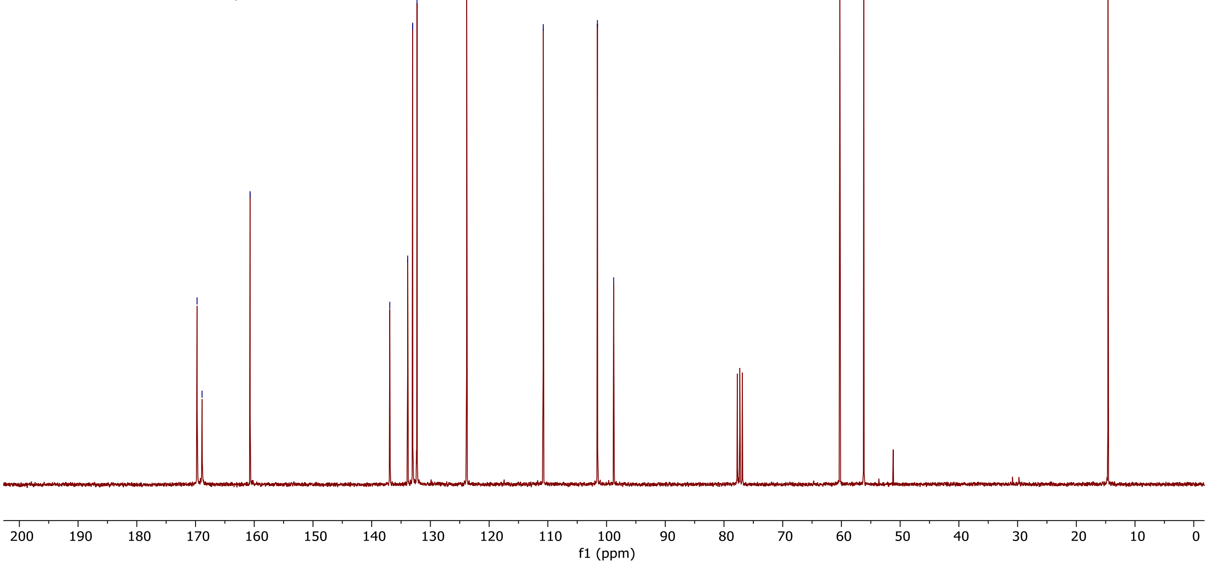

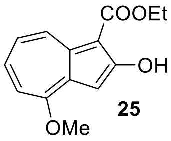

25

^{1}H-NMR, 75 MHz, $CDCl_3$

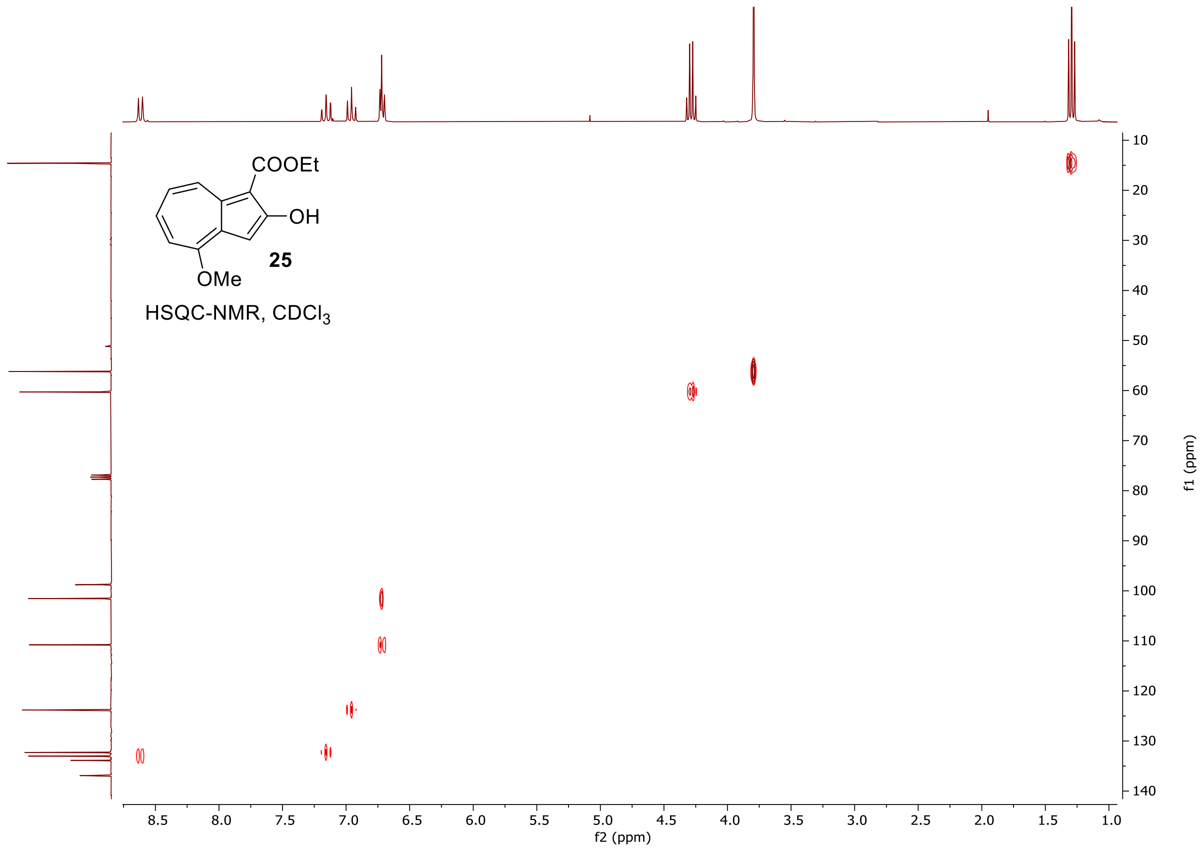
COOEt
OH
25
OMe
HSQC-NMR, CDCl3
f1 (ppm)
f2 (ppm)

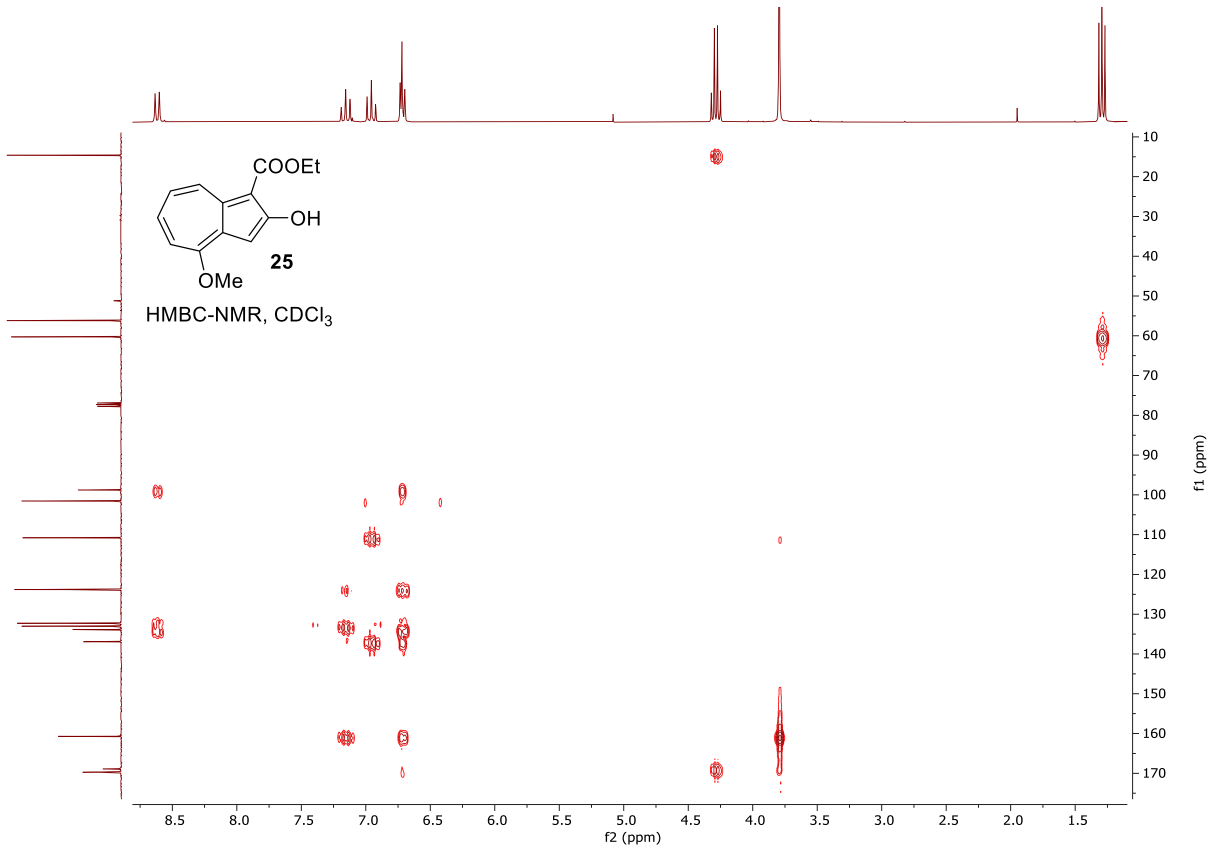
COOEt
OH
25
OMe
HMBC-NMR, CDCl3
f1 (ppm)
f2 (ppm)

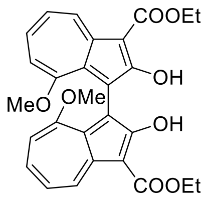

26

^{1}H-NMR, 250 MHz, $CDCl_3$

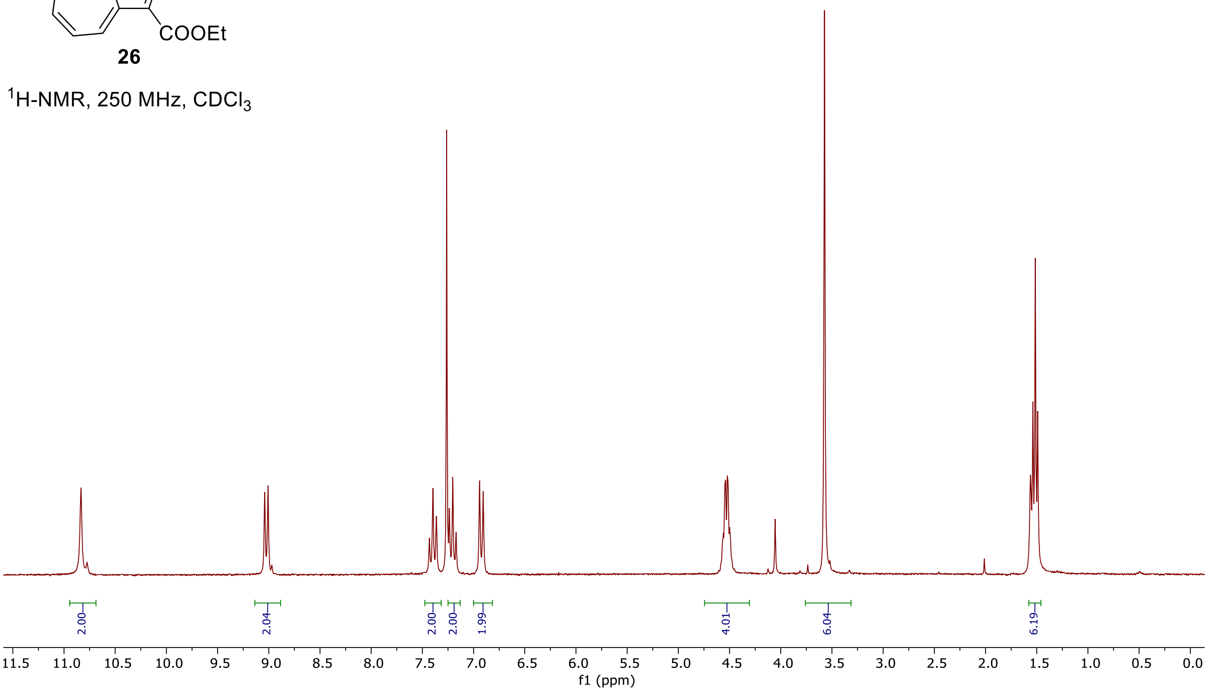

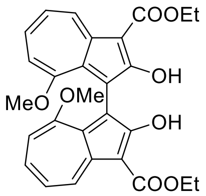

26

^{13}C-NMR, 75 MHz, $CDCl_3$

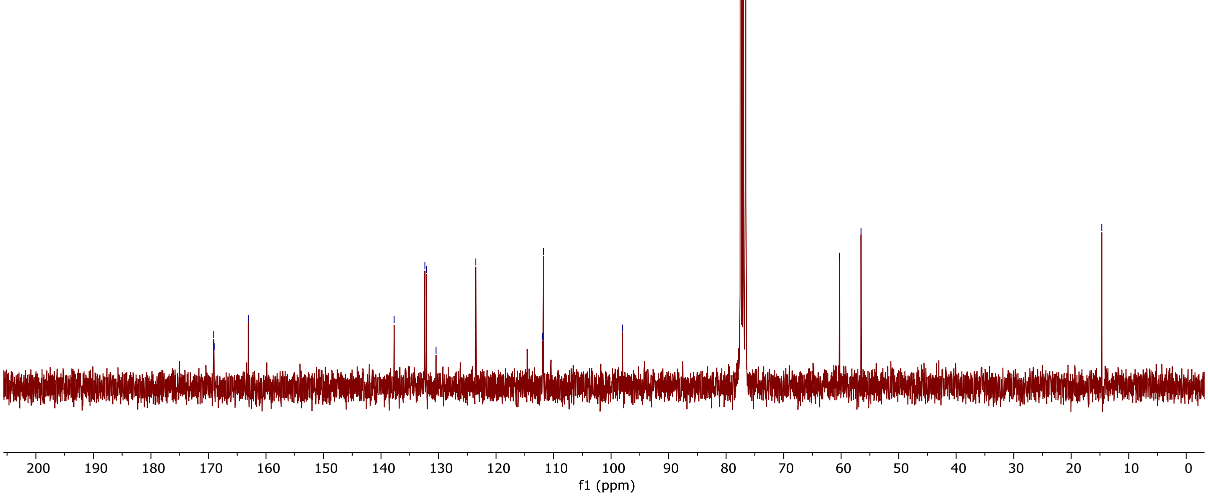

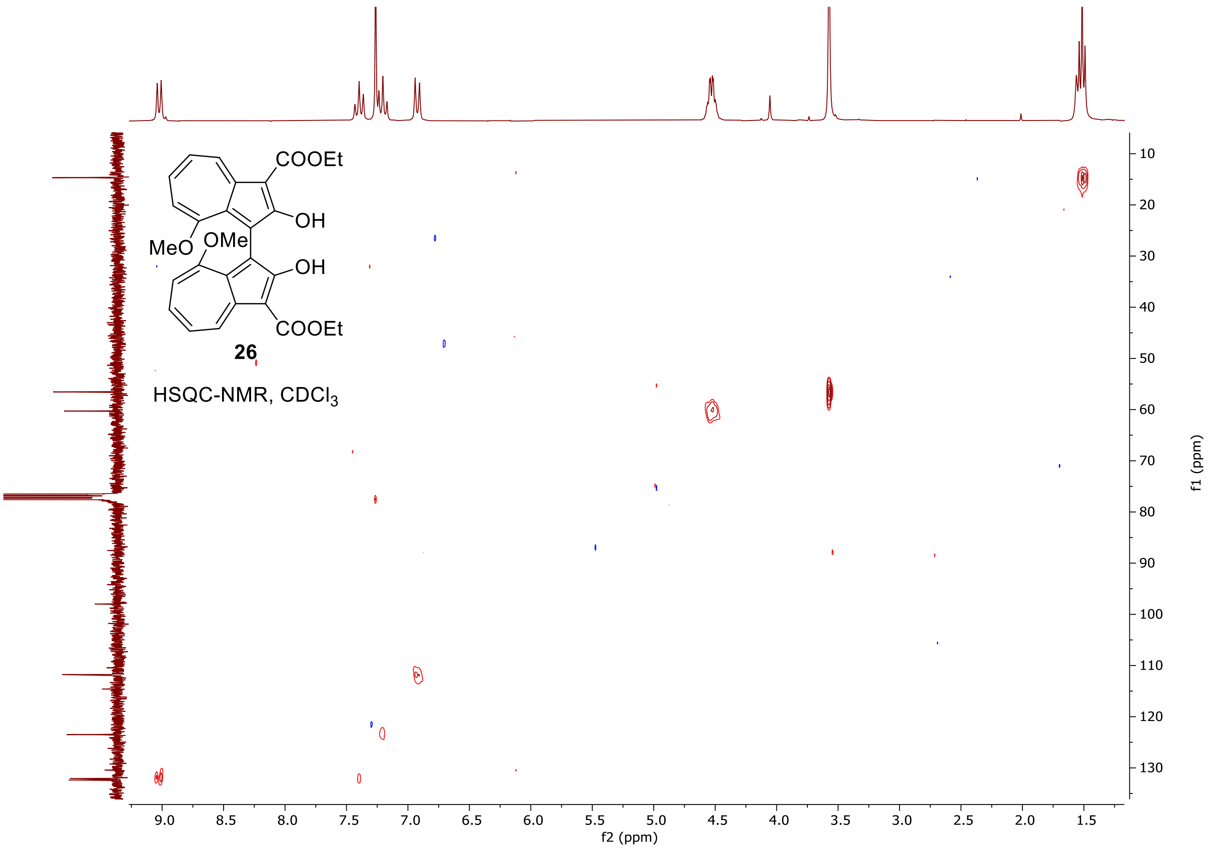
COOEt
OH
MeO
OMe
OH
COOEt
26
HSQC-NMR, CDCl3
f2 (ppm)
f1 (ppm)

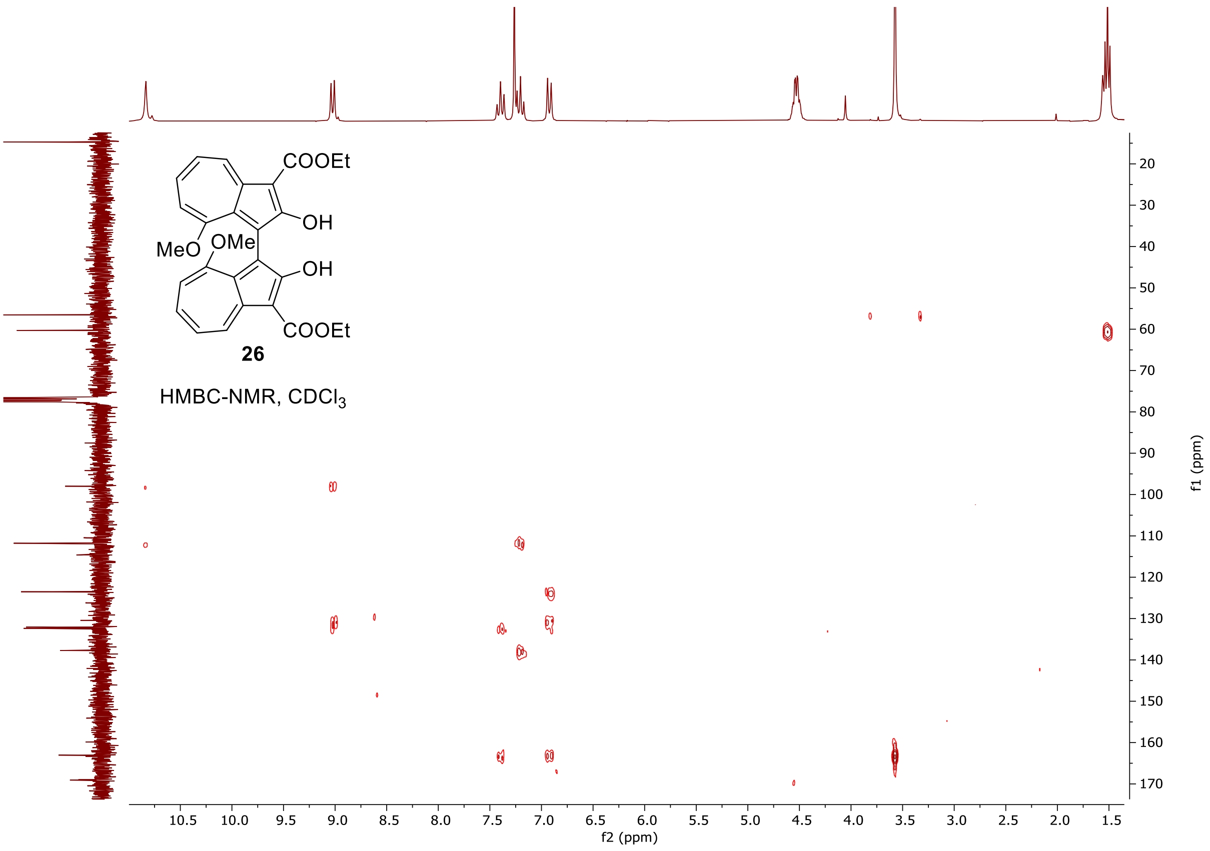
COOEt
OH
MeO
OMe
OH
COOEt
26
HMBC-NMR, CDCl3
f2 (ppm)
f1 (ppm)

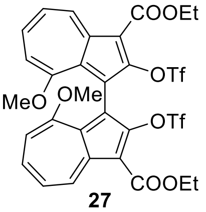

27

^{1}H-NMR, 500 MHz, $CDCl_3$

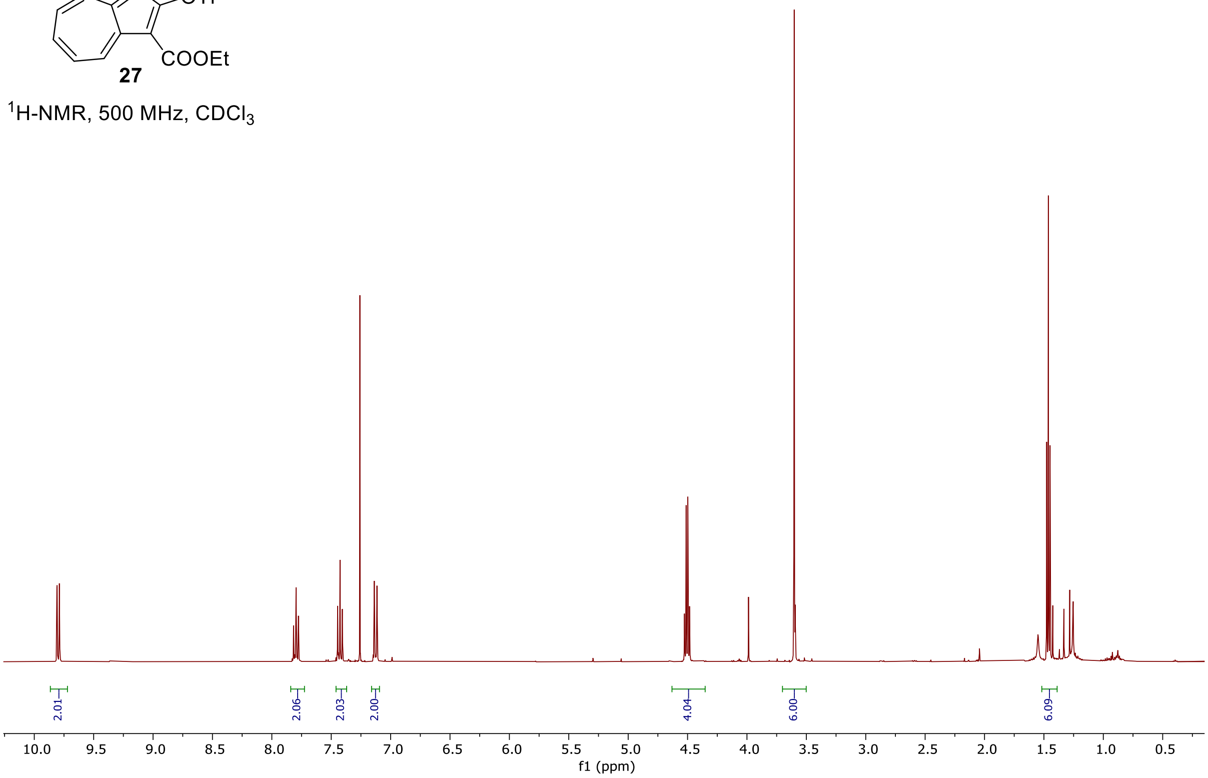

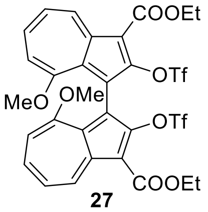

27

^{13}C-NMR, 126 MHz, $CDCl_3$

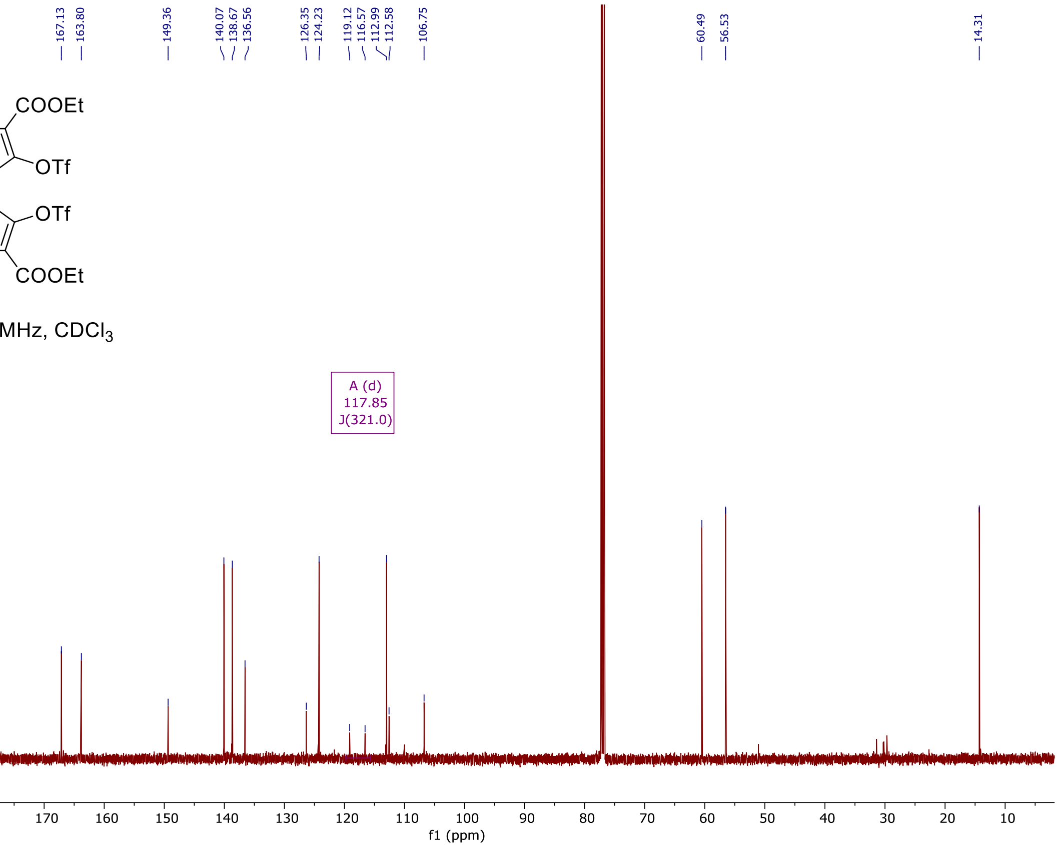

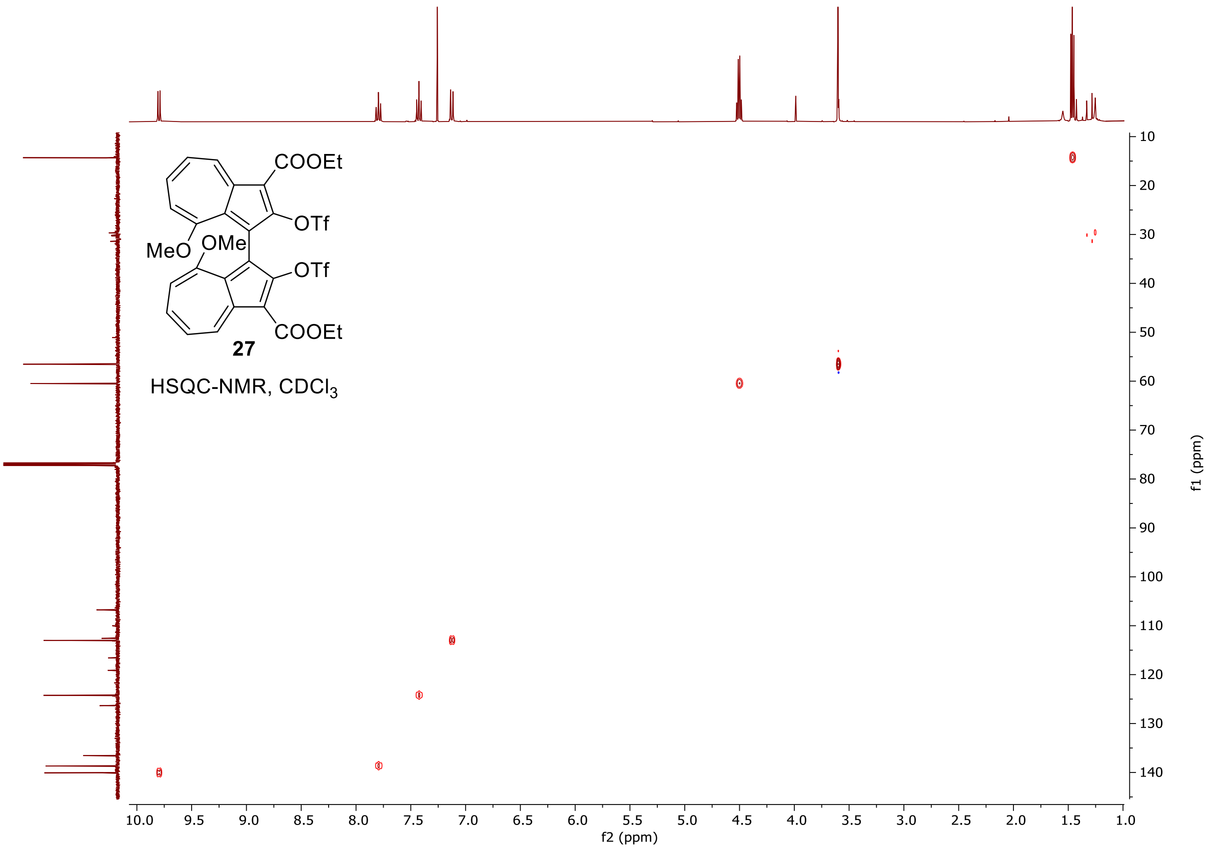
COOEt
OTf
MeO
OMe
OTf
COOEt
27
HSQC-NMR, CDCl3
f2 (ppm)
f1 (ppm)

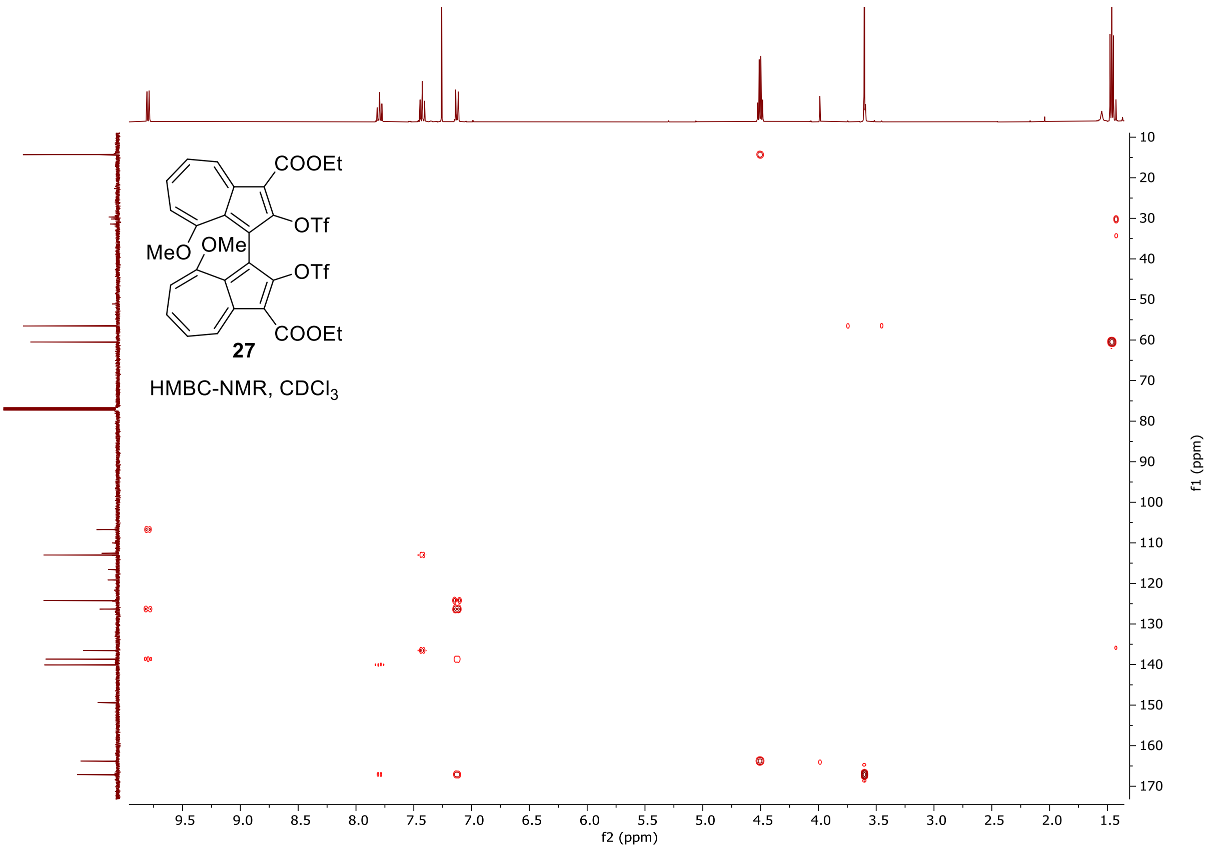
COOEt
OTf
MeO
OMe
OTf
COOEt
27
HMBC-NMR, CDCl3
f2 (ppm)
f1 (ppm)

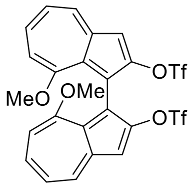

28

^{1}H-NMR, 500 MHz, $CDCl_3$

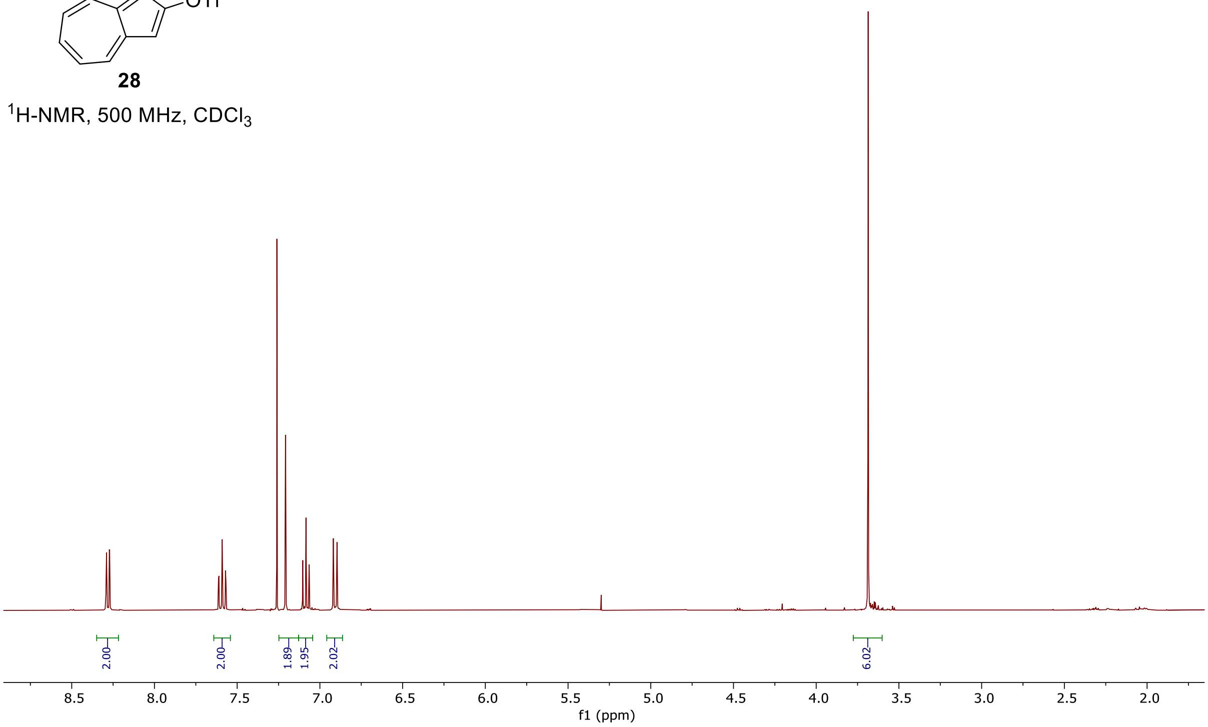

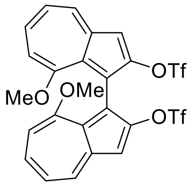

28

^{13}C-NMR, 126 MHz, $CDCl_3$

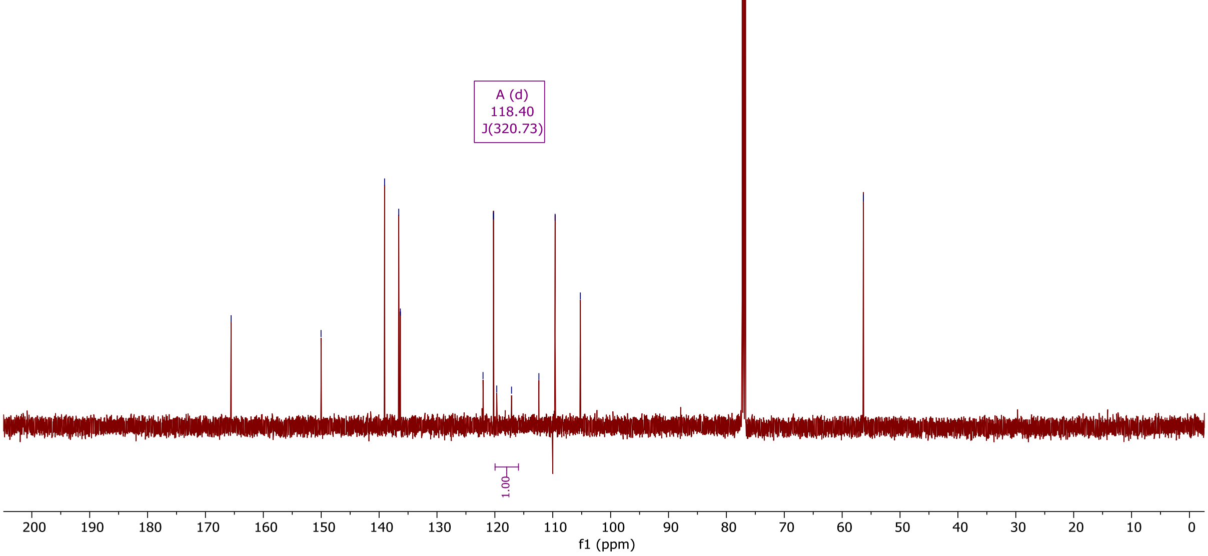

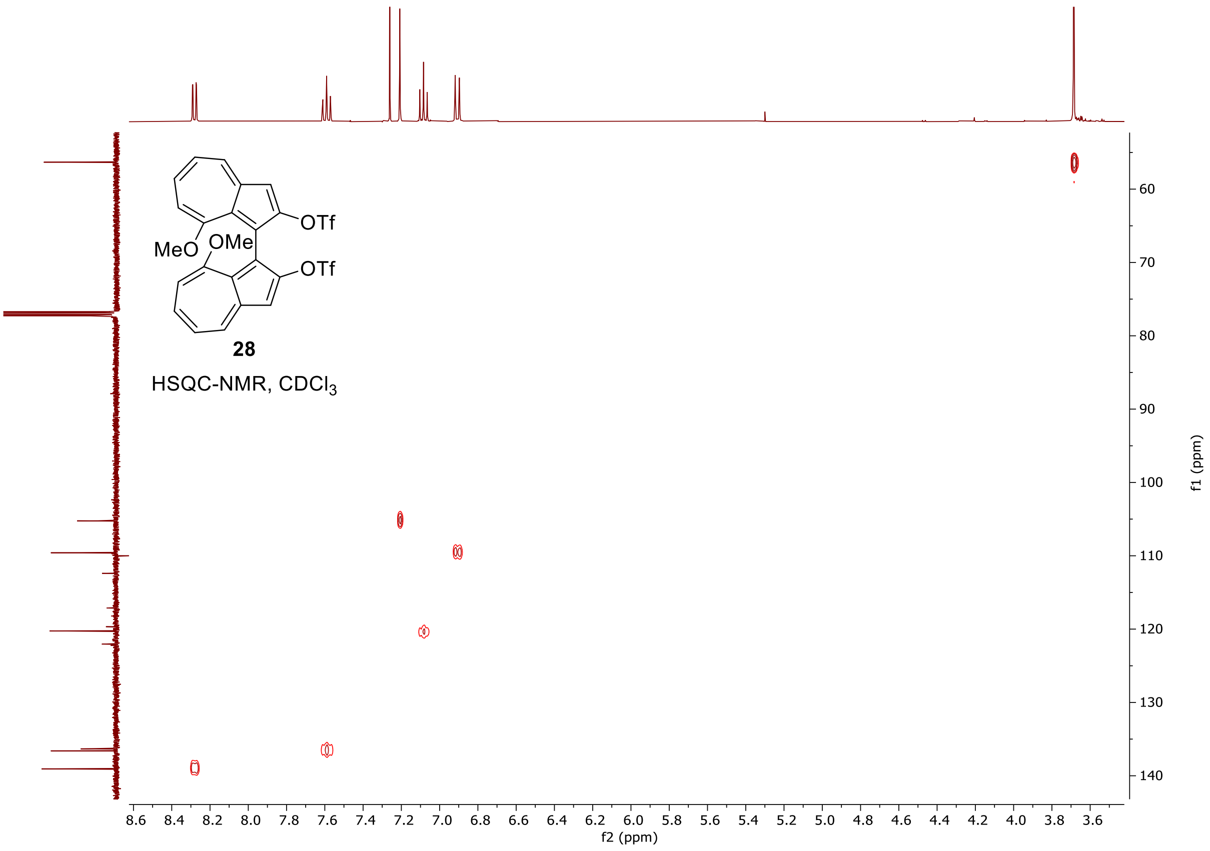
OTf
MeO
OMe
OTf
28
HSQC-NMR, $CDCl_3$
f2 (ppm)
f1 (ppm)

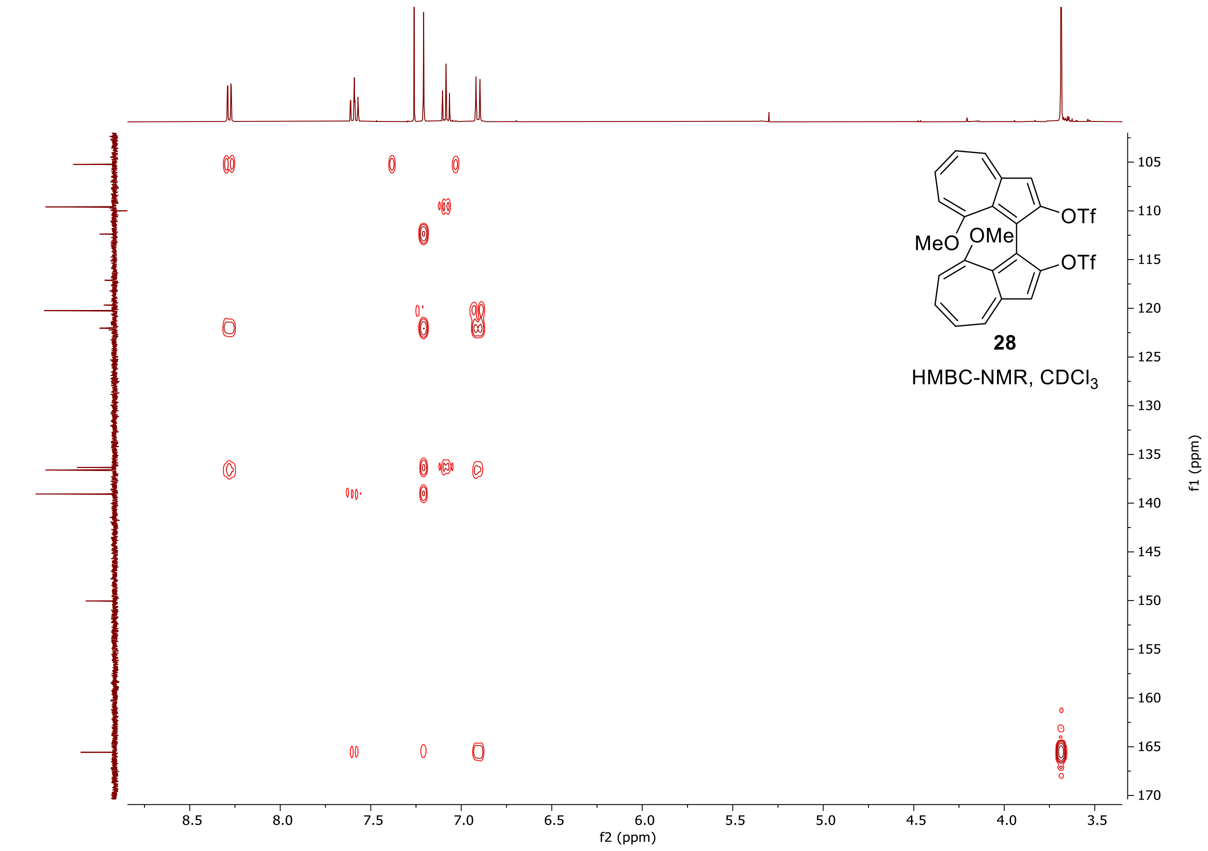
OTf
MeO
OMe
OTf
28
HMBC-NMR, $CDCl_3$
f2 (ppm)
f1 (ppm)

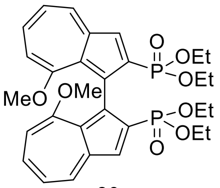

30

^{1}H-NMR, 500 MHz, $CDCl_3$

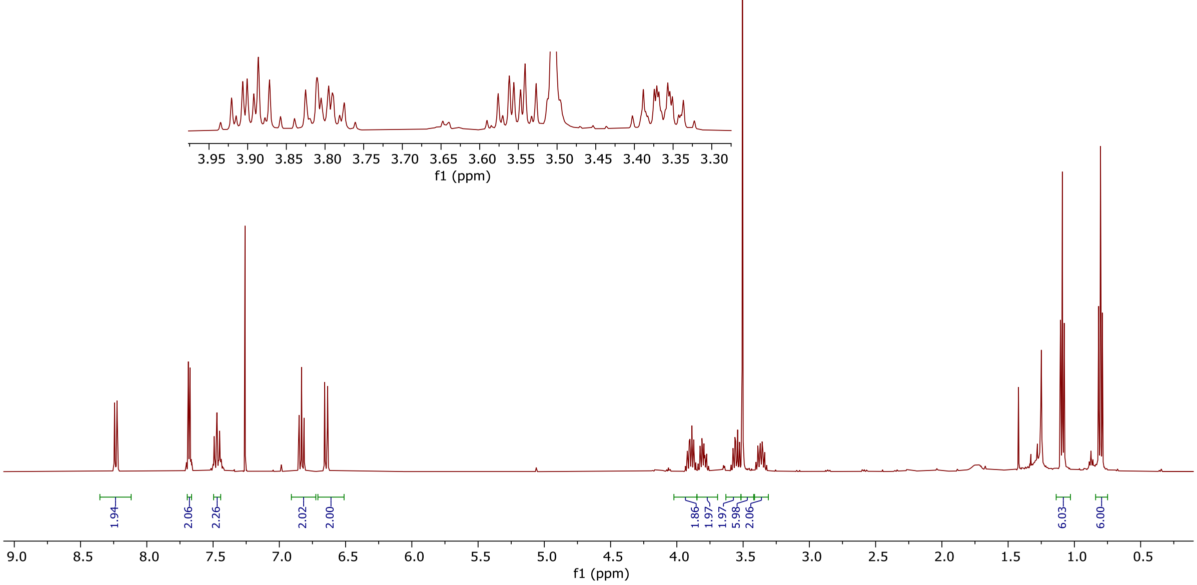

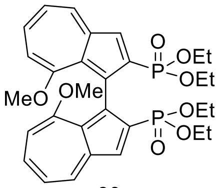

30

^{31}P-NMR, 202 MHz, $CDCl_3$

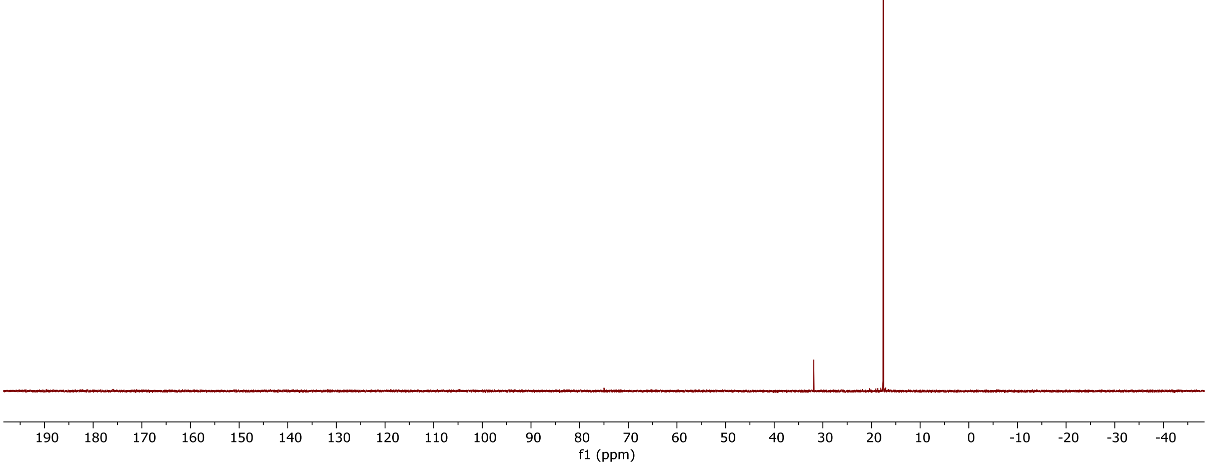

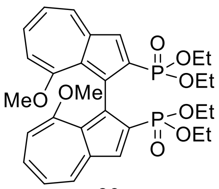

30

^{13}C-NMR, 125 MHz, $CDCl_3$

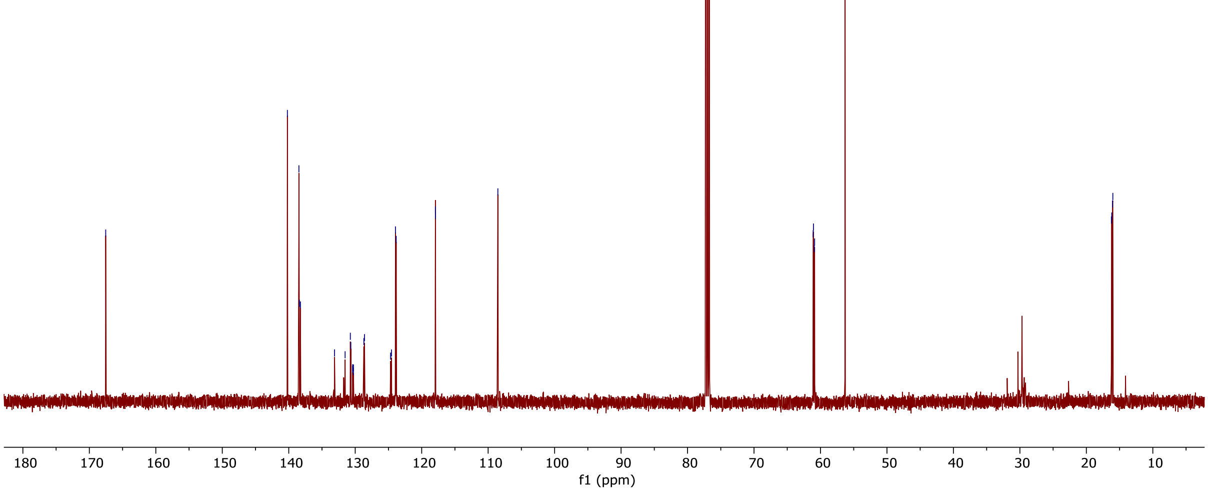

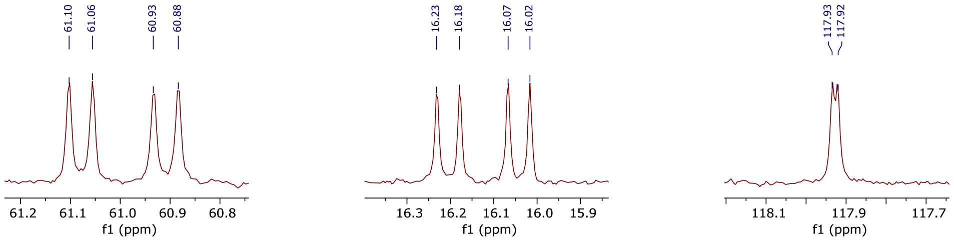

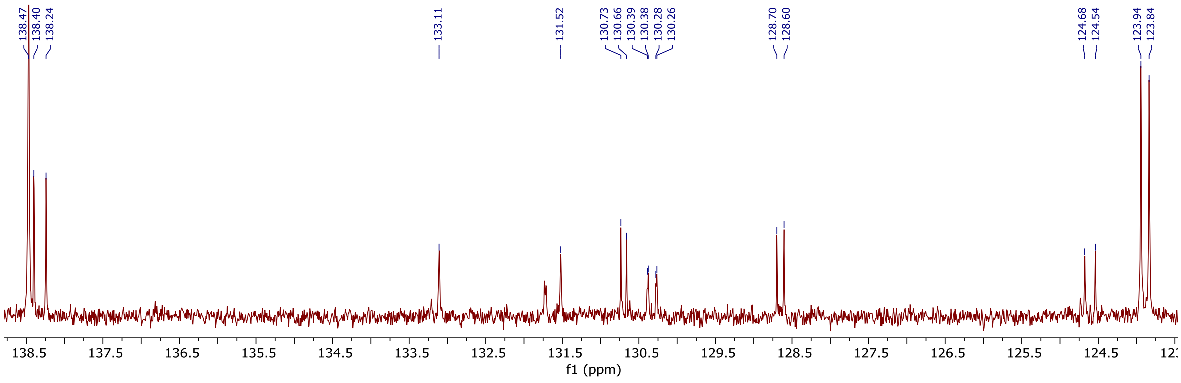

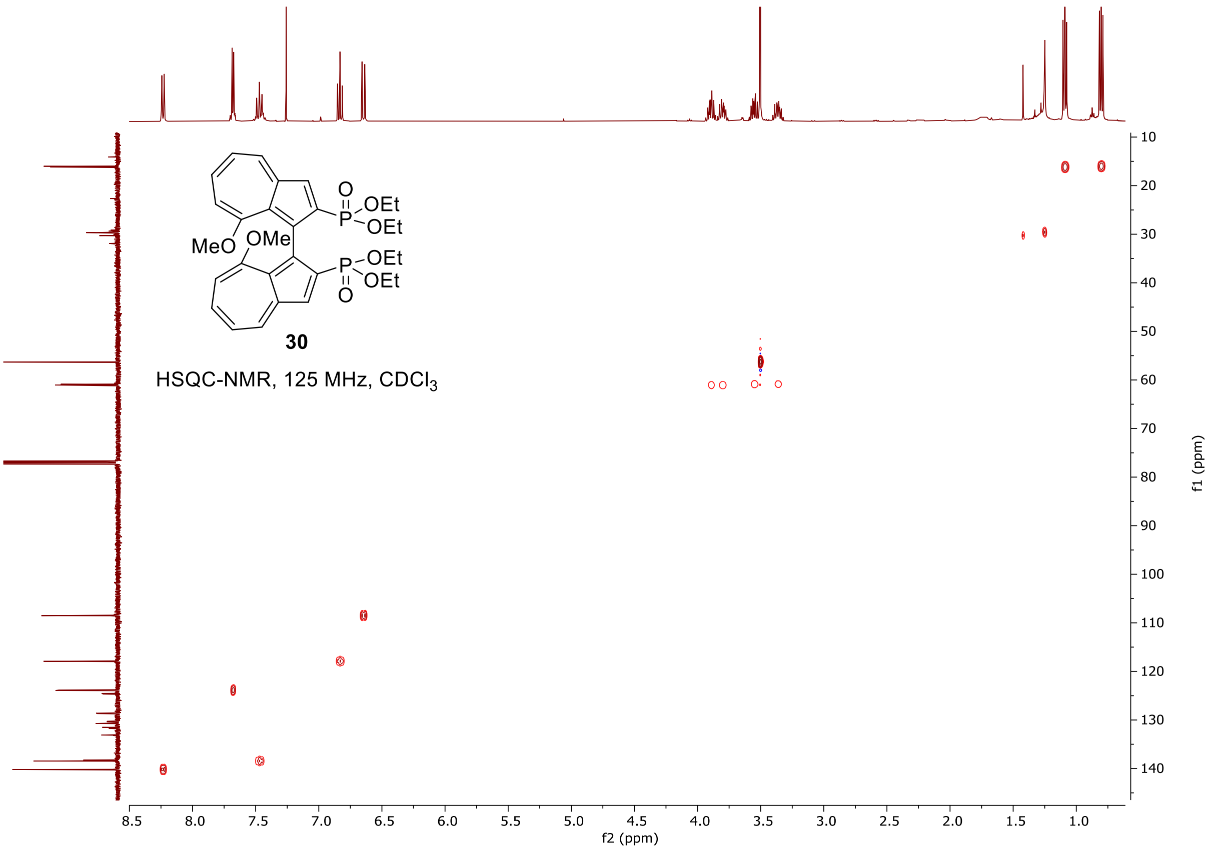

O
P
OEt
OEt
MeO
OMe
P
OEt
OEt
O
30
HSQC-NMR, 125 MHz, CDCl3
f1 (ppm)
f2 (ppm)

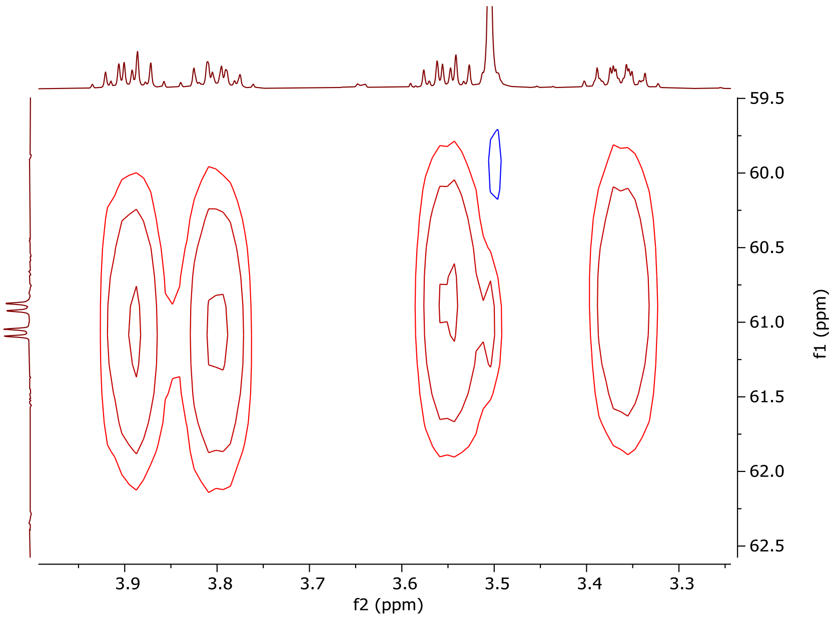

f1 (ppm)
f2 (ppm)

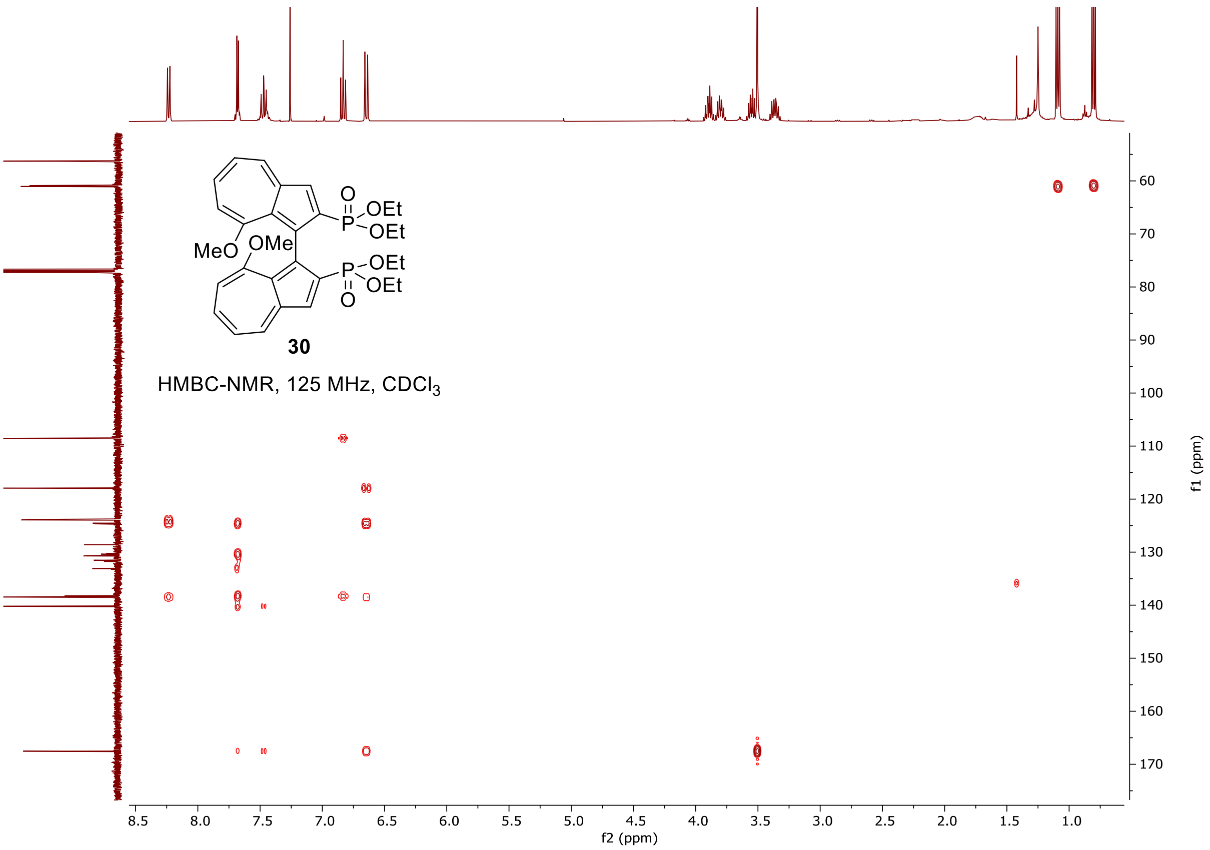
O
OEt
OEt
MeO
OMe
OEt
OEt
O
30
HMBC-NMR, 125 MHz, $CDCl_3$
f2 (ppm)
f1 (ppm)

X-ray Crystallography

Table S3. Crystal data and structure refinement for (*R*a,1*R*,2*S*,5*R*)-**22**.

Identification code	s16sel1
Empirical formula	C52.30 H66.90 O12.15
Formula weight	889.95
Temperature	150.01(10) K
Wavelength	1.54184 Å
Crystal system	Orthorhombic
Space group	$P2_12_12_1$
Unit cell dimensions	a = 12.61370(10) Å α= 90°.
b = 16.68850(10) Å	β= 90°.
c = 23.7719(2) Å	γ = 90°.
Volume	5004.07(7) $Å^3$
Z	4
Density (calculated)	1.181 Mg/m^3
Absorption coefficient	0.675 mm^{-1}
F(000)	1912
Crystal size	0.300 x 0.250 x 0.080 mm^3
Theta range for data collection	3.236 to 73.128°.
Index ranges	-15<=h<=15, -19<=k<=20, -22<=l<=29
Reflections collected	57209
Independent reflections	9957 [R(int) = 0.0304]
Completeness to theta = 67.684°	100.0 %
Absorption correction	Semi-empirical from equivalents
Max. and min. transmission	1.00000 and 0.71916
Refinement method	Full-matrix least-squares on F^2
Data / restraints / parameters	9957 / 24 / 634
Goodness-of-fit on F^2	1.027
Final R indices [I>2sigma(I)]	R1 = 0.0326, wR2 = 0.0829
R indices (all data)	R1 = 0.0351, wR2 = 0.0846
Absolute structure parameter	0.06(4)
Extinction coefficient	n/a
Largest diff. peak and hole	0.136 and -0.143 e.$Å^{-3}$

CCDC #2421193

Table S4. Crystal data and structure refinement for (S_a,1R,2S,5R)-**22**.

Identification code	s16sel11	
Empirical formula	C52 H66 O12	
Formula weight	883.04	
Temperature	150.00(10) K	
Wavelength	1.54184 Å	
Crystal system	Orthorhombic	
Space group	$P2_12_12_1$	
Unit cell dimensions	a = 12.4714(15) Å	α= 90°.
b = 17.3833(7) Å	β= 90°.	
c = 22.554(2) Å	γ = 90°.	
Volume	4889.6(8) Å^3	
Z	4	
Density (calculated)	1.200 Mg/m^3	
Absorption coefficient	0.684 mm^{-1}	
F(000)	1896	
Crystal size	0.300 x 0.050 x 0.020 mm^3	
Theta range for data collection	3.210 to 72.497°.	
Index ranges	-15<=h<=15, -14<=k<=21, -27<=l<=26	
Reflections collected	36824	
Independent reflections	9561 [R(int) = 0.0975]	
Completeness to theta = 67.684°	100.0 %	
Absorption correction	Semi-empirical from equivalents	
Max. and min. transmission	1.00000 and 0.55306	
Refinement method	Full-matrix least-squares on F^2	
Data / restraints / parameters	9561 / 137 / 638	
Goodness-of-fit on F^2	0.897	
Final R indices [I>2sigma(I)]	R1 = 0.0651, wR2 = 0.1316	
R indices (all data)	R1 = 0.1585, wR2 = 0.1682	
Absolute structure parameter	0.0(2)	
Extinction coefficient	n/a	
Largest diff. peak and hole	0.237 and -0.186 e.Å^{-3}	

CCDC #2421194

Table S5. Crystal data and structure refinement for (R_a)-**20.**

Identification code	s16sel7
Empirical formula	C30 H30 O8
Formula weight	518.54
Temperature	150.00(10) K
Wavelength	1.54184 Å
Crystal system	Orthorhombic
Space group	$P2_12_12_1$
Unit cell dimensions	a = 7.0912(3) Å	α= 90°.
b = 9.4533(4) Å	β= 90°.
c = 37.611(2) Å	γ = 90°.
Volume	2521.3(2) Å^3
Z	4
Density (calculated)	1.366 Mg/m^3
Absorption coefficient	0.817 mm^{-1}
F(000)	1096
Crystal size	0.180 x 0.050 x 0.020 mm^3
Theta range for data collection	4.824 to 68.414°.
Index ranges	-8<=h<=8, -6<=k<=11, -45<=l<=45
Reflections collected	13591
Independent reflections	4633 [R(int) = 0.0483]
Completeness to theta = 67.684°	99.8 %
Absorption correction	Semi-empirical from equivalents
Max. and min. transmission	1.00000 and 0.71798
Refinement method	Full-matrix least-squares on F^2
Data / restraints / parameters	4633 / 0 / 355
Goodness-of-fit on F^2	1.124
Final R indices [I>2sigma(I)]	R1 = 0.0643, wR2 = 0.1503
R indices (all data)	R1 = 0.0725, wR2 = 0.1545
Absolute structure parameter	-0.11(17)
Extinction coefficient	n/a
Largest diff. peak and hole	0.309 and -0.305 e.Å^{-3}

CCDC #2421195

References

[1] CrysAlisPro CrysAlisPro 1.171.38.41- CrysAlisPro 1.171.43.104a (Rigaku OD, 2015-2023)

[2] G. M. Sheldrick, Crystal structure refinement with SHELXL, *Acta Cryst.*, 2015, **C71**, 3-8.

[3] C. B. Hübschle, G. M. Sheldrick and B. Dittrich, ShelXle: a Qt graphical user interface for SHELXL, *J. Appl. Cryst.*, 2011, **44**, 1281-1284.

[4] C. F. Macrae, P. R. Edgington, P. McCabe, E. Pidcock, G. P. Shields, R. Taylor, M. Towler and J. van der Streek, Mercury: visualization and analysis of crystal structures, *J. Appl. Cryst.*, 2006, **39**, 453-457.

[5] W. von Eggers Doering and C. F. Hiskey, Reactions of halotropones and related compounds, *J. Am. Chem. Soc.*, 1952, **74**, 5688-5693.

[6] R. M. Claramunt, D. Sanz, M. Pérez-Torralba, E. Pinilla and M. R. Torres, Solid-state structure and tautomerism of 2-aminotroponimines studied by x-ray crystallography and multinuclear NMR spectroscopy, *Eur. J. Org. Chem.*, 2004, 4452-4466.

[7] T. Nozoe, T. Asao, M. Yasunami, H. Wakui, T. Suzuki and A. Masayoshi, Formation and structure of 2-diazo-2,4-azulenequinone derivatives, *J. Org. Chem.*, 1995, **60**, 5919-5924.

[8] W. Pham, R. Weissleder and C. Tung, Intermolecular [8+2] cycloaddition reactions of 2*H*-3-methoxycarbonylcyclohepta[*b*]furan-2-one with vinyl ethers: an approach to bicyclo[5.3.0]azulene derivatives, *Tetrahedron Lett.*, 2002, **43**, 19-20.

[9] T. Nozoe, H. Wakabayashi, K. Shindo, S. Ishikawa, C.-P. Wu and P.-W. Yang, A convenient, one-pot azulene synthesis from 2*H*-cyclohepta[*b*]furan-2-ones with vinyl ether and its analogues **III**. Orthoesters as a reagent, *Heterocycles*, 1991, **32**, 213-220.

[10] Z. Li, X. Liang, F. Wu and B. Wan, A convenient resolution method for 1,1′-bi-2-naphthol and 4,4′-dibromo-1,1′-spirobiindane-7,7′-diol with menthyl chloroformate in the presence of TBAB, *Tetrahedron: Asymmetry*, 2004, **15**, 665-669.

[11] M. Kalek, M. Jezowska and J. Stawinski, Preparation of arylphosphonates by palladium(0)-catalyzed cross-coupling in the presence of acetate additives: synthetic and mechanistic studies, *Adv. Synth. Catal.*, 2009, **351**, 3207-3216.
